# Supplementary material for: Dermal PapillaCell-Derived Exosomes Regulate Hair Follicle Stem Cell Proliferation via LEF1
Source: Int J Mol Sci. 2023 Feb 16;24(4):3961. doi: 10.3390/ijms24043961 (PMC9964005; doi:10.3390/ijms24043961)
Supplement: Supplementary file 1 [file ijms-24-03961-s001.zip › ijms-2164188-supplementary.pdf]

Table S1. Differentially expressed genes between HFSC+Exos and HFSC-Control groups.

Gene ID	foldChange (Exos/HFSC)	log2FoldChange	Gene symbol	Up/down-regulated
ENSOCUG00000006482	191.6352348	7.582219034	IFI44	Up
ENSOCUG00000007488	46.46360995	5.538029342	SUSD2	Up
ENSOCUG00000005445	39.39924772	5.300096178	-	Up
ENSOCUG00000021037	173.9861801	7.442828906	-	Up
ENSOCUG00000021924	77.21583789	6.270824887	-	Up
ENSOCUG00000012233	34.4990751	5.10848578	GGT5	Up
ENSOCUG00000008656	38.29247794	5.258989116	-	Up
ENSOCUG00000024378	51.60283043	5.689378295	IFI44L	Up
ENSOCUG00000023263	31.99449602	4.999751836	NDUFA4L2	Up
ENSOCUG00000006355	58.64226752	5.873868984	DDX60	Up
ENSOCUG00000034782	339.597127	8.407680443	OAS1	Up
ENSOCUG00000004501	84.34212313	6.398181434	-	Up
ENSOCUG00000000968	21.22990012	4.408025679	DES	Up
ENSOCUG00000039234	19.09950113	4.255463051	-	Up
ENSOCUG00000006146	21.77831529	4.444820451	PAX7	Up
ENSOCUG00000007472	17.69885299	4.145583961	-	Up
ENSOCUG00000000416	21.29012854	4.412112755	ARMCX2	Up
ENSOCUG00000004197	78.47647824	6.294188395	-	Up
ENSOCUG00000011128	25.81995914	4.690414812	GLI1	Up
ENSOCUG00000009797	16.67116275	4.059282825	ABCA4	Up
ENSOCUG00000012591	16.59064286	4.052297884	UCP2	Up
ENSOCUG00000002998	17.52695354	4.13150335	PKDCC	Up
ENSOCUG00000021735	14.80654335	3.888162971	-	Up
ENSOCUG00000000560	17.65625484	4.142107453	HEYL	Up
ENSOCUG00000014988	14.17842981	3.825625865	COL3A1	Up
ENSOCUG00000014931	35.80609546	5.162133301	-	Up
ENSOCUG00000007680	29.01902202	4.858926994	-	Up
ENSOCUG00000008236	13.95193948	3.802393782	LPL	Up
ENSOCUG00000014125	17.33126373	4.115304949	PGM5	Up
ENSOCUG00000015049	13.15750636	3.717814187	DTX4	Up
ENSOCUG00000010772	14.838003	3.891225032	DCHS2	Up
ENSOCUG00000001412	13.57199389	3.762560781	IGFBP6	Up
ENSOCUG00000014633	12.86721097	3.685627472	PKNOX2	Up
ENSOCUG00000006595	30.65438284	4.938021454	UBA7	Up
ENSOCUG00000022666	11.81202626	3.562184564	-	Up
ENSOCUG00000000312	13.68196474	3.774203512	IGDCC4	Up
ENSOCUG00000008557	20.19496097	4.335923453	ADCY2	Up
ENSOCUG00000013498	12.41719947	3.634267924	ADAMTS4	Up
ENSOCUG00000002473	11.7172698	3.550564547	OLFML2A	Up
ENSOCUG00000011683	14.04982537	3.812480294	ANK1	Up
ENSOCUG00000013629	17.1711079	4.101911222	COL2A1	Up

ENSOCUG00000022596	19.18451487	4.261870378	AK5	Up
ENSOCUG00000009757	11.54832349	3.529611521	-	Up
ENSOCUG00000003418	11.44731451	3.516937283	PARP14	Up
ENSOCUG00000013304	16.91249612	4.080017698	-	Up
ENSOCUG00000013049	11.07878903	3.469728291	PLXND1	Up
ENSOCUG00000027981	61.13791105	5.933995354	ISG15	Up
ENSOCUG00000000276	11.28465155	3.496289966	MOV10	Up
ENSOCUG00000007208	23.86456153	4.576797925	MSC	Up
ENSOCUG00000032132	44.2853816	5.468758645	IFIT3	Up
ENSOCUG00000015723	13.93995489	3.801153987	SLC15A3	Up
ENSOCUG00000006561	234.934087	7.876112242	-	Up
ENSOCUG00000031506	10.63669913	3.410978605	-	Up
ENSOCUG00000002699	16.41992929	4.037376009	PARP9	Up
ENSOCUG00000016568	21.1153487	4.400220167	HEY1	Up
ENSOCUG00000003982	34.92232593	5.126077745	CORO2B	Up
ENSOCUG00000027849	72.21954678	6.174317461	OAS3	Up
ENSOCUG00000025477	10.84947472	3.43955329	MYF5	Up
ENSOCUG00000006497	9.808917068	3.294093868	PXDN	Up
ENSOCUG00000029637	76.63258992	6.25988616	-	Up
ENSOCUG00000022631	15.84022205	3.985520655	ZNF536	Up
ENSOCUG00000012881	9.441307214	3.238986625	COL1A1	Up
ENSOCUG00000024112	11.31109395	3.499666561	LGALS3BP	Up
ENSOCUG00000003866	9.251726855	3.209722673	FBN1	Up
ENSOCUG00000013278	18.12255351	4.179714343	DHX58	Up
ENSOCUG00000012264	10.35759638	3.37261734	COL1A2	Up
ENSOCUG00000024734	14.12553021	3.820233116	-	Up
ENSOCUG00000027535	9.560092838	3.257024628	-	Up
ENSOCUG00000007482	9.925247595	3.311103092	CX3CL1	Up
ENSOCUG00000003313	9.226205586	3.20573744	LOX	Up
ENSOCUG00000013517	12.57997142	3.653056739	-	Up
ENSOCUG00000003371	9.813594338	3.294781636	SELENBP1	Up
ENSOCUG00000004055	16.36151696	4.032234609	WDR86	Up
ENSOCUG00000014417	14.00857089	3.808237879	-	Up
ENSOCUG00000002114	11.7052907	3.549088858	TRIM5	Up
ENSOCUG00000007432	9.779529199	3.289765013	ACACB	Up
ENSOCUG00000005785	16.86471784	4.075936276	ADGRB2	Up
ENSOCUG00000004579	8.493304823	3.086326029	NRP1	Up
ENSOCUG00000003636	9.559665409	3.256960124	TGM2	Up
ENSOCUG00000016280	13.2469109	3.727584066	CXCL10	Up
ENSOCUG00000016911	9.233778147	3.20692107	TDRD12	Up
ENSOCUG00000007449	9.494982608	3.247165358	-	Up
ENSOCUG00000011474	8.738163069	3.127330029	RNF157	Up
ENSOCUG00000029328	72.52984204	6.180502802	OASL	Up
ENSOCUG00000023498	9.358521835	3.226280676	TBX2	Up

ENSOCUG00000002562	10.71612472	3.421711372	PODN	Up
ENSOCUG00000013232	8.002938437	3.000529811	COL4A1	Up
ENSOCUG00000015823	67.41661883	6.075032368	OAS2	Up
ENSOCUG00000008781	8.437509652	3.076817247	ADAM12	Up
ENSOCUG00000023340	41.17037002	5.36353451	SIGLEC15	Up
ENSOCUG00000006002	12.47532861	3.641005913	-	Up
ENSOCUG00000024055	8.56939246	3.099192926	MCAM	Up
ENSOCUG00000016744	10.02551947	3.325605086	SPOCK2	Up
ENSOCUG00000007286	8.919463584	3.156956949	COL4A4	Up
ENSOCUG00000000092	8.014578649	3.002626676	SERPINF1	Up
ENSOCUG00000013022	7.847582926	2.972248369	-	Up
ENSOCUG00000014857	7.730439174	2.950550377	COL5A3	Up
ENSOCUG00000017566	13.83135879	3.789870989	ARNT2	Up
ENSOCUG00000005669	9.983808215	3.319590221	ADGRD1	Up
ENSOCUG00000003213	7.741180372	2.952553565	PARP12	Up
ENSOCUG00000004418	7.773864803	2.958632018	CREB3L1	Up
ENSOCUG00000027205	11.26763695	3.49411308	SIX2	Up
ENSOCUG00000015872	14.68611462	3.876380859	-	Up
ENSOCUG00000010231	9.216557946	3.204228056	CHRNE	Up
ENSOCUG00000009603	7.533358817	2.913293247	FAM114A1	Up
ENSOCUG00000004707	11.13727708	3.47732465	OLFM2	Up
ENSOCUG00000003874	18.81243103	4.233614387	-	Up
ENSOCUG00000023913	10.97609618	3.456293124	-	Up
ENSOCUG00000006105	11.29261839	3.497308134	CDHR1	Up
ENSOCUG00000015829	10.76131071	3.427781901	FGD3	Up
ENSOCUG00000025358	7.803212512	2.964068191	PROS1	Up
ENSOCUG00000038763	7.278092268	2.863560341	ATOX1	Up
ENSOCUG00000014268	7.794077431	2.962378264	SIX1	Up
ENSOCUG00000011161	11.9782005	3.582339281	LRRC17	Up
ENSOCUG00000014444	13.61203318	3.766810668	HDAC9	Up
ENSOCUG00000003187	9.032210087	3.175079043	REM1	Up
ENSOCUG00000008523	7.208784062	2.849755934	FLT1	Up
ENSOCUG00000022422	35.98446836	5.169302439	COLCA2	Up
ENSOCUG00000004881	26.0710665	4.704377696	AMOT	Up
ENSOCUG00000011779	8.064999344	3.011674416	TNFRSF19	Up
ENSOCUG00000006034	7.270375694	2.862029917	FHL1	Up
ENSOCUG00000017892	10.838623	3.438109576	ZC3HAV1	Up
ENSOCUG00000022941	13.4277906	3.747150039	DTX3L	Up
ENSOCUG00000001752	12.63297541	3.659122567	GPC3	Up
ENSOCUG00000002794	10.02404533	3.325392938	ZNF618	Up
ENSOCUG00000002800	Inf	Inf	KIF12	Up
ENSOCUG00000007504	8.371783959	3.065535082	VSTM4	Up
ENSOCUG00000013276	6.758250457	2.756649817	COL4A2	Up
ENSOCUG00000000041	9.502366392	3.248286835	NKX2-5	Up

ENSOCUG00000001607	8.666274158	3.115411877	-	Up
ENSOCUG00000006006	11.97971487	3.582521666	SLC28A1	Up
ENSOCUG00000006190	6.988037211	2.804887291	NCAM1	Up
ENSOCUG00000027149	7.674042793	2.93998681	-	Up
ENSOCUG00000017723	11.09677715	3.472068829	CAVIN2	Up
ENSOCUG00000005767	15.14070892	3.920360852	HMGCS2	Up
ENSOCUG00000013564	12.01060166	3.586236518	TRIM63	Up
ENSOCUG00000013489	17.30485998	4.113105363	-	Up
ENSOCUG00000013105	13.58737669	3.764195037	PLCG2	Up
ENSOCUG00000026441	21.85125329	4.449644123	-	Up
ENSOCUG00000025148	6.66674817	2.736983232	-	Up
ENSOCUG00000014935	6.966286736	2.800389856	-	Up
ENSOCUG00000014915	21.22917108	4.407976135	STAC2	Up
ENSOCUG00000007694	224.5560915	7.810932048	CNIH3	Up
ENSOCUG00000007190	11.99827867	3.58475554	-	Up
ENSOCUG00000003269	8.565996718	3.098621124	MYRF	Up
ENSOCUG00000000080	7.052940809	2.818224932	PLEKHA2	Up
ENSOCUG00000002553	6.735237371	2.751728792	EMILIN2	Up
ENSOCUG00000006947	10.4564447	3.386320499	PROB1	Up
ENSOCUG00000026736	7.805227484	2.96444068	SGCA	Up
ENSOCUG00000023917	7.347545612	2.87726241	EBF4	Up
ENSOCUG00000002898	7.261468395	2.860261316	FBLN7	Up
ENSOCUG00000010104	6.335724578	2.663509621	FN1	Up
ENSOCUG00000016945	6.623029556	2.727491296	ITIH5	Up
ENSOCUG00000014625	6.454001906	2.690194004	HEG1	Up
ENSOCUG00000002828	6.411010699	2.680551816	IGFBP4	Up
ENSOCUG00000001499	7.04392392	2.816379327	PLEKHA4	Up
ENSOCUG00000002988	7.830191146	2.969047526	ATP1B2	Up
ENSOCUG00000025884	6.902848223	2.787191763	RCAN2	Up
ENSOCUG00000003380	10.51632537	3.394558779	FGFR4	Up
ENSOCUG00000005094	6.690530082	2.742120518	INHBB	Up
ENSOCUG00000007319	7.708722292	2.946491756	GPX7	Up
ENSOCUG00000007728	6.522083972	2.705333016	TTYH2	Up
ENSOCUG00000015039	6.667069788	2.737052829	FNDC1	Up
ENSOCUG00000025675	7.851359923	2.972942563	EDN3	Up
ENSOCUG00000027464	17.77940792	4.152135376	SALL2	Up
ENSOCUG00000026922	6.213904437	2.635500055	ADAMTS7	Up
ENSOCUG00000009655	6.738875927	2.752507964	LAMA4	Up
ENSOCUG00000008768	25.78059425	4.688213614	-	Up
ENSOCUG00000001473	8.993448187	3.168874367	S100B	Up
ENSOCUG00000017440	6.112203376	2.611692548	ERFE	Up
ENSOCUG00000000716	7.336759335	2.875142962	EPSTI1	Up
ENSOCUG00000011415	7.488463823	2.904669796	TGFB3	Up
ENSOCUG00000006673	7.769028063	2.957734123	A4GALT	Up

ENSOCUG00000011360	6.406646261	2.679569335	SLC4A3	Up
ENSOCUG00000017305	6.184209795	2.628589263	COQ8A	Up
ENSOCUG00000014480	6.200580924	2.632403386	C1R	Up
ENSOCUG00000006145	14.57609806	3.865532665	CHRDL1	Up
ENSOCUG00000005827	6.438525453	2.686730321	-	Up
ENSOCUG00000015151	30.05403541	4.909486812	NME9	Up
ENSOCUG00000004894	7.424244368	2.892244197	PHYHD1	Up
ENSOCUG00000007492	7.291414349	2.866198688	LMOD1	Up
ENSOCUG00000011042	6.445751899	2.688348659	COL8A2	Up
ENSOCUG00000014953	6.224907253	2.638052342	SYNE3	Up
ENSOCUG00000002188	7.229476089	2.853891101	FES	Up
ENSOCUG00000011145	5.994807921	2.583713529	OBSL1	Up
ENSOCUG00000008855	5.98120839	2.580436983	-	Up
ENSOCUG00000000611	5.934294877	2.569076617	FKBP10	Up
ENSOCUG00000007685	7.632274326	2.932113027	ANGPTL2	Up
ENSOCUG00000011484	6.023342135	2.590564208	TNFAIP2	Up
ENSOCUG00000009526	6.009864071	2.587332361	VASH1	Up
ENSOCUG00000008635	6.314195044	2.658598826	-	Up
ENSOCUG00000009222	5.635781774	2.494615749	VIM	Up
ENSOCUG00000003913	6.006733596	2.58658068	TRIM14	Up
ENSOCUG00000012337	6.117025233	2.612830227	COL21A1	Up
ENSOCUG00000002131	6.998090296	2.80696128	PSMB8	Up
ENSOCUG00000027804	14.74666957	3.882317264	SOBP	Up
ENSOCUG00000001035	5.585217057	2.481613349	ADAMTS2	Up
ENSOCUG00000006516	6.135081365	2.617082478	ZEB1	Up
ENSOCUG00000002339	18.02794876	4.17216335	IRF7	Up
ENSOCUG00000004472	5.407503065	2.434962578	PLPP1	Up
ENSOCUG00000012217	8.228525273	3.040633892	APCDD1	Up
ENSOCUG00000022014	5.524844743	2.465933923	NOTCH3	Up
ENSOCUG00000001844	12.82215559	3.680566915	PYCR1	Up
ENSOCUG00000000409	8.397292115	3.069924175	-	Up
ENSOCUG00000005247	5.499027594	2.459176526	FSTL1	Up
ENSOCUG00000007753	6.402616616	2.678661625	THY1	Up
ENSOCUG00000015163	9.544292518	3.25463826	NYNRIN	Up
ENSOCUG00000005141	16.94935877	4.083158789	-	Up
ENSOCUG00000039017	5.995075794	2.583777993	METTL27	Up
ENSOCUG00000027605	15.89607032	3.990598254	ISL1	Up
ENSOCUG00000025772	5.787739031	2.532999872	DLG4	Up
ENSOCUG00000005845	6.211367288	2.634910879	-	Up
ENSOCUG00000029649	11.5894112	3.534735367	HEY2	Up
ENSOCUG00000004484	9.816427418	3.295198067	GLRB	Up
ENSOCUG00000022122	33.8262849	5.080072831	ZNF467	Up
ENSOCUG00000011746	5.773885217	2.529542428	NR4A1	Up
ENSOCUG00000006382	5.992978439	2.583273183	ZCCHC24	Up

ENSOCUG00000021885	10.04054657	3.327765901	-	Up
ENSOCUG00000012685	5.521594264	2.46508488	PITX2	Up
ENSOCUG00000000224	7.600228415	2.926042778	-	Up
ENSOCUG00000026233	52.77545226	5.721795132	PLAAT2	Up
ENSOCUG00000015136	6.227711096	2.638702019	NIBAN1	Up
ENSOCUG00000027718	7.082542966	2.824267449	PRSS56	Up
ENSOCUG00000013540	6.045954801	2.595970194	EPHB2	Up
ENSOCUG00000025830	5.712087559	2.514018094	CAPN5	Up
ENSOCUG00000016390	7.918779271	2.985278047	CCN4	Up
ENSOCUG00000014794	10.07075028	3.332099265	ATP2A3	Up
ENSOCUG00000013403	6.711783718	2.746696227	L3HYPDH	Up
ENSOCUG00000013696	5.308702852	2.408359391	BICC1	Up
ENSOCUG00000001536	14.97449442	3.904435389	-	Up
ENSOCUG00000032044	5.232200976	2.387417958	-	Up
ENSOCUG00000026029	5.181326814	2.373321585	-	Up
ENSOCUG00000001754	5.377112812	2.42683174	FMNL3	Up
ENSOCUG00000007631	11.05508237	3.46663787	GPM6A	Up
ENSOCUG00000014596	5.121472164	2.356558572	LOXL2	Up
ENSOCUG00000008288	10.28193962	3.36204054	ECM2	Up
ENSOCUG00000023670	8.628649521	3.109134779	RAMP2	Up
ENSOCUG00000001711	8.018435744	3.00332082	TRO	Up
ENSOCUG00000011158	10.08308988	3.333865903	FAM131B	Up
ENSOCUG00000017761	12.97171974	3.697297854	TCF21	Up
ENSOCUG00000026479	5.508942886	2.461775506	SERPING1	Up
ENSOCUG00000027972	9.193671512	3.20064112	-	Up
ENSOCUG00000010972	5.076407587	2.343807908	-	Up
ENSOCUG00000006596	6.288606558	2.652740377	CHST1	Up
ENSOCUG00000027792	6.799033397	2.765329656	TMEM52	Up
ENSOCUG00000032150	9.163725037	3.19593417	FDCSP	Up
ENSOCUG00000022774	11.39335438	3.510120656	-	Up
ENSOCUG00000012279	7.107910706	2.829425557	ARMH4	Up
ENSOCUG00000001920	5.808722847	2.538220996	GPRC5B	Up
ENSOCUG00000010507	8.169900855	3.030318571	NPR3	Up
ENSOCUG00000006310	9.439161298	3.238658677	PPM1J	Up
ENSOCUG00000022125	6.913579355	2.789432828	IL34	Up
ENSOCUG00000000810	5.147323963	2.363822586	PRICKLE2	Up
ENSOCUG00000006982	5.436215005	2.442602517	-	Up
ENSOCUG00000012661	5.758945232	2.525804602	PTPRU	Up
ENSOCUG00000013720	5.547131105	2.471741824	CLYBL	Up
ENSOCUG00000031924	17.37112978	4.118619682	-	Up
ENSOCUG00000009699	5.509523125	2.461927452	KIF5C	Up
ENSOCUG00000027729	8.112284949	3.020108329	-	Up
ENSOCUG00000004623	8.021211647	3.00382018	GALNT16	Up
ENSOCUG00000032540	8.285825062	3.050645361	-	Up

ENSOCUG00000013367	4.871769313	2.284445821	COL11A1	Up
ENSOCUG00000007857	5.594644384	2.484046431	LRRK2	Up
ENSOCUG00000009306	5.596029498	2.484403567	LARP6	Up
ENSOCUG00000021185	7.484476957	2.903901499	FNDC5	Up
ENSOCUG00000014468	5.738083321	2.520568917	C1S	Up
ENSOCUG00000029611	10.64148524	3.411627618	IL7	Up
ENSOCUG00000026159	8.983677687	3.167306168	KIAA1755	Up
ENSOCUG00000022907	14.72709084	3.880400566	RORC	Up
ENSOCUG00000037302	6.749951085	2.754877047	GPR4	Up
ENSOCUG00000003273	5.180113769	2.372983784	ITGA2B	Up
ENSOCUG00000008181	5.70896448	2.513229086	-	Up
ENSOCUG00000003680	6.567278663	2.715295673	GSTA4	Up
ENSOCUG00000003165	5.366149634	2.423887284	SCUBE2	Up
ENSOCUG00000011733	6.381454358	2.673885257	AKAP5	Up
ENSOCUG00000003298	4.94568824	2.3061713	-	Up
ENSOCUG00000010618	6.905777973	2.787803951	ANK2	Up
ENSOCUG00000015469	5.824716616	2.542187861	CTSO	Up
ENSOCUG00000025123	8.612511954	3.10643408	GCNT4	Up
ENSOCUG00000023882	5.133576363	2.359964246	GRIK5	Up
ENSOCUG00000002733	5.254420748	2.393531729	CACNA1G	Up
ENSOCUG00000014676	4.715013672	2.237261954	LAMB1	Up
ENSOCUG00000039461	Inf	Inf	FAM110D	Up
ENSOCUG00000002052	5.181227184	2.373293844	LRP4	Up
ENSOCUG00000012691	5.779602115	2.530970177	GJC1	Up
ENSOCUG00000029688	40.86865769	5.352922955	HERC6	Up
ENSOCUG00000022385	4.749852011	2.247882565	-	Up
ENSOCUG00000000131	5.396220933	2.431949417	NXPE3	Up
ENSOCUG00000010955	5.388711388	2.42994032	HSPG2	Up
ENSOCUG00000010681	5.290656169	2.403446662	PAPLN	Up
ENSOCUG00000005631	5.689287939	2.508248099	-	Up
ENSOCUG00000017504	8.906845819	3.154914621	SYT9	Up
ENSOCUG00000012087	8.616380627	3.107081982	EFHC1	Up
ENSOCUG00000004590	5.045116042	2.334887453	-	Up
ENSOCUG00000024308	5.063144707	2.340033718	MMP11	Up
ENSOCUG00000038415	5.513540568	2.462979056	-	Up
ENSOCUG00000015805	8.304713778	3.053930446	NOX5	Up
ENSOCUG00000014702	13.13419844	3.715256252	-	Up
ENSOCUG00000039007	11.96018341	3.580167608	LENEP	Up
ENSOCUG00000003924	10.71664729	3.421781723	-	Up
ENSOCUG00000016347	13.54572687	3.759765907	PHOSPHO1	Up
ENSOCUG00000025341	11.10454436	3.473078293	ADSS1	Up
ENSOCUG00000010740	5.724712617	2.517203271	TARS3	Up
ENSOCUG00000027672	6.907131685	2.788086729	PPFIA4	Up
ENSOCUG00000017667	90.88867532	6.506028641	ATP1A2	Up

ENSOCUG00000005513	4.46796886	2.159619131	FKBP5	Up
ENSOCUG00000001553	4.707560936	2.23497977	LIPA	Up
ENSOCUG00000000638	4.699239813	2.232427394	GLI2	Up
ENSOCUG00000009543	8.1632491	3.029143482	KCNMA1	Up
ENSOCUG00000008985	5.62399012	2.49159406	EBF1	Up
ENSOCUG00000008754	7.037086473	2.814978241	CAMKV	Up
ENSOCUG00000016321	5.734676545	2.519712116	GLT8D2	Up
ENSOCUG00000029555	9.866990319	3.302610093	-	Up
ENSOCUG00000002973	5.005875852	2.323622512	PTCH1	Up
ENSOCUG00000015882	5.768884805	2.528292456	EPDR1	Up
ENSOCUG00000014205	5.147115615	2.363764189	AKAP6	Up
ENSOCUG00000016632	5.093675584	2.348707077	PDE7B	Up
ENSOCUG00000029735	6.758947135	2.75679853	-	Up
ENSOCUG00000016007	4.509494789	2.172965814	NID1	Up
ENSOCUG00000025757	7.438905887	2.895090446	TMEM200B	Up
ENSOCUG00000004447	7.013693855	2.810174459	ALPL	Up
ENSOCUG00000002455	4.369067267	2.127325318	STAT1	Up
ENSOCUG00000008915	6.201446628	2.632604796	EBF3	Up
ENSOCUG00000011916	4.684747334	2.22797124	SNTA1	Up
ENSOCUG00000000008	Inf	Inf	CCIN	Up
ENSOCUG00000014993	4.703763795	2.233815615	DENND2A	Up
ENSOCUG00000006729	7.392528694	2.886067938	GRK3	Up
ENSOCUG00000001519	4.631868001	2.21159414	RTN4RL1	Up
ENSOCUG00000023406	6.216397999	2.636078874	PPP1R14A	Up
ENSOCUG00000002139	15.33559913	3.938812626	-	Up
ENSOCUG00000008356	67.58181984	6.078563295	-	Up
ENSOCUG00000014113	4.879418973	2.286709366	-	Up
ENSOCUG00000021405	7.877331951	2.977707072	MYOD1	Up
ENSOCUG00000013369	4.490233627	2.166790511	-	Up
ENSOCUG00000017198	5.63949592	2.495566215	TUB	Up
ENSOCUG00000013552	7.227643242	2.853525296	KCNN3	Up
ENSOCUG00000010666	4.857711433	2.280276791	-	Up
ENSOCUG00000003764	4.210070019	2.073844228	-	Up
ENSOCUG00000017133	8.311948677	3.055186746	GDPD2	Up
ENSOCUG00000006288	5.137714715	2.361126783	NINL	Up
ENSOCUG00000002736	4.977185919	2.315330279	SEMA6C	Up
ENSOCUG00000038788	5.029205787	2.330330587	-	Up
ENSOCUG00000037248	5.135530617	2.360513347	GPC6	Up
ENSOCUG00000014957	8.827282691	3.1419694	FRMD3	Up
ENSOCUG00000035699	10.18849654	3.348869272	-	Up
ENSOCUG00000005991	4.683240575	2.22750715	ALPK3	Up
ENSOCUG00000030778	6.334147881	2.66315055	-	Up
ENSOCUG00000009017	12.25419681	3.615204023	OGN	Up
ENSOCUG00000027132	11.59438696	3.535354635	-	Up

ENSOCUG00000001522	15.96967728	3.997263254	-	Up
ENSOCUG00000000227	4.610591303	2.204951787	ASAP3	Up
ENSOCUG00000015278	16.22840316	4.020449144	PIPOX	Up
ENSOCUG00000006251	4.818545456	2.268597715	ADAM2	Up
ENSOCUG00000006408	4.370845054	2.127912236	DISP1	Up
ENSOCUG00000017377	9.419052694	3.23558197	MATN2	Up
ENSOCUG00000009702	5.043715625	2.334486936	FMOD	Up
ENSOCUG00000014661	4.554000983	2.187134603	NAAA	Up
ENSOCUG00000010959	10.43511046	3.383373967	ZIC4	Up
ENSOCUG00000015575	4.472960604	2.161230052	GABBR1	Up
ENSOCUG00000013248	5.280513652	2.400678272	SLC25A43	Up
ENSOCUG00000008892	7.158486966	2.839654688	RAPSN	Up
ENSOCUG00000012610	4.418540225	2.143569818	CDON	Up
ENSOCUG00000005760	4.905219462	2.294317685	TMEM63C	Up
ENSOCUG00000005033	10.45808074	3.386546208	DGAT2L6	Up
ENSOCUG00000022067	4.712584743	2.236518562	DLX4	Up
ENSOCUG00000000581	4.589832125	2.198441388	NAGLU	Up
ENSOCUG00000007916	15.91812084	3.992598128	GCNT1	Up
ENSOCUG00000023147	4.599838918	2.20158334	PDLIM4	Up
ENSOCUG00000004215	4.558850752	2.188670179	FGF11	Up
ENSOCUG00000000974	8.569359033	3.099187298	-	Up
ENSOCUG00000015932	6.724560002	2.749439873	-	Up
ENSOCUG00000023898	6.510286204	2.702720968	CCDC8	Up
ENSOCUG00000026781	5.955967392	2.574335855	F5	Up
ENSOCUG00000005225	5.279167661	2.400310485	LURAP1	Up
ENSOCUG00000035940	6.502581815	2.701012646	-	Up
ENSOCUG00000006151	4.199389821	2.070179717	PDIA5	Up
ENSOCUG00000013463	6.285414073	2.65200779	MELTF	Up
ENSOCUG00000003928	4.715301097	2.237349898	SEPTIN6	Up
ENSOCUG00000009113	7.390531929	2.885678205	PDE1C	Up
ENSOCUG00000025973	10.74985091	3.426244746	POPDC3	Up
ENSOCUG00000006872	5.53932003	2.469708892	EMILIN1	Up
ENSOCUG00000011016	4.34359857	2.118890777	-	Up
ENSOCUG00000005073	35.46634461	5.148378739	-	Up
ENSOCUG00000023629	4.425599299	2.145872833	TPCN1	Up
ENSOCUG00000015652	4.799798923	2.262973969	RECK	Up
ENSOCUG00000003161	6.161359225	2.623248651	NRIP3	Up
ENSOCUG00000001701	5.559011046	2.474828249	FOXF1	Up
ENSOCUG00000010592	4.080922777	2.028895411	BMP1	Up
ENSOCUG00000002690	4.131147032	2.046542408	ACSF2	Up
ENSOCUG00000027790	5.617031648	2.489807931	NEURL1B	Up
ENSOCUG00000014602	4.11014328	2.039188687	ITGB5	Up
ENSOCUG00000007470	4.394466798	2.135688127	ZBTB16	Up
ENSOCUG00000001481	9.407239974	3.233771507	RIPOR2	Up

ENSOCUG00000030705	11.410689	3.512314001	GPR162	Up
ENSOCUG00000022094	4.322261587	2.111786388	C1QTNF5	Up
ENSOCUG00000024221	98.32062608	6.619422197	INMT	Up
ENSOCUG00000008172	4.702061822	2.233293507	-	Up
ENSOCUG00000015240	7.845227725	2.971815325	SLC2A4	Up
ENSOCUG00000025680	4.472252471	2.161001635	LRRC75A	Up
ENSOCUG00000000076	4.345834266	2.119633156	SLC43A2	Up
ENSOCUG00000017203	6.608582262	2.724340804	GNAO1	Up
ENSOCUG00000038704	6.02301438	2.590485703	-	Up
ENSOCUG00000009218	5.708074093	2.513004062	PDE10A	Up
ENSOCUG00000014174	4.196058014	2.069034624	ZNF395	Up
ENSOCUG00000027817	34.30546287	5.100366427	PPP1R16B	Up
ENSOCUG00000003528	4.78092846	2.257290818	ATOH8	Up
ENSOCUG00000010508	4.04066607	2.014593129	IDH2	Up
ENSOCUG00000013467	6.59594176	2.72157866	RNF150	Up
ENSOCUG00000002435	4.318432253	2.110507657	-	Up
ENSOCUG00000005096	5.75482979	2.524773259	HUNK	Up
ENSOCUG00000005324	4.100023845	2.0356323	MAP1A	Up
ENSOCUG00000009276	4.46798598	2.159624659	MYBPC1	Up
ENSOCUG00000010718	3.943784581	1.97958075	TIMP1	Up
ENSOCUG00000016660	9.201843673	3.201922947	SFRP2	Up
ENSOCUG00000002740	5.057255926	2.338354789	RPS6KA2	Up
ENSOCUG00000000857	4.342769331	2.118615325	FUT10	Up
ENSOCUG00000021287	5.135305007	2.360449967	HSPB6	Up
ENSOCUG00000001039	4.36651318	2.126481695	SYNGAP1	Up
ENSOCUG00000025762	4.012981677	2.004674569	PGPEP1	Up
ENSOCUG00000024108	5.483535094	2.455106262	-	Up
ENSOCUG00000003606	4.080163027	2.028626798	KIF21B	Up
ENSOCUG00000009512	5.683451831	2.506767413	PLA1A	Up
ENSOCUG00000010563	3.983830893	1.994156409	TAPBP	Up
ENSOCUG00000022364	4.833865323	2.273177279	-	Up
ENSOCUG00000002537	9.201465124	3.201863596	ROBO1	Up
ENSOCUG00000009428	4.477062709	2.162552526	RAB6B	Up
ENSOCUG00000017803	16.66519127	4.05876597	-	Up
ENSOCUG00000008424	4.054284532	2.019447341	-	Up
ENSOCUG00000025111	72.04564994	6.170839419	GALNT15	Up
ENSOCUG00000007327	4.236329123	2.082814677	CNTNAP1	Up
ENSOCUG00000016774	7.227064043	2.853409679	ECHDC2	Up
ENSOCUG00000029119	5.25255869	2.393020377	ZNF454	Up
ENSOCUG00000033095	3.935516918	1.976553142	-	Up
ENSOCUG00000002857	3.955382919	1.983817368	PSME2	Up
ENSOCUG00000021480	8.725834598	3.125293127	FOXS1	Up
ENSOCUG00000009858	4.204965365	2.072093917	KLHL30	Up
ENSOCUG00000004347	10.16356787	3.345335036	SCN4A	Up

ENSOCUG00000013125	4.84862885	2.277576823	ACSS1	Up
ENSOCUG00000000440	49.30901805	5.623779618	CYP7B1	Up
ENSOCUG00000013689	3.961789471	1.986152218	LAPTM4B	Up
ENSOCUG00000038328	10.75765572	3.427291819	-	Up
ENSOCUG00000017251	3.715241811	1.893456113	GBP1	Up
ENSOCUG00000000470	4.078962723	2.028202323	-	Up
ENSOCUG00000021203	5.077042653	2.34398838	DNPH1	Up
ENSOCUG00000000805	4.436922654	2.149559404	FKBP14	Up
ENSOCUG00000029603	4.367416066	2.126779977	ZNF74	Up
ENSOCUG00000021648	6.328029152	2.661756246	KCNK12	Up
ENSOCUG00000017676	6.119643475	2.613447605	-	Up
ENSOCUG00000002662	4.027258613	2.009798119	-	Up
ENSOCUG00000001616	4.81706239	2.26815361	PCSK5	Up
ENSOCUG00000002095	4.344745597	2.119271704	NR1H3	Up
ENSOCUG00000014973	3.878326726	1.955434347	GALK1	Up
ENSOCUG00000009692	4.036615223	2.013146073	-	Up
ENSOCUG00000003685	4.698470209	2.232191101	ZNF423	Up
ENSOCUG00000001305	4.373647788	2.128837044	PHETA2	Up
ENSOCUG00000012903	5.322764355	2.412175698	SIM2	Up
ENSOCUG00000004979	3.800984922	1.926373302	KDELR3	Up
ENSOCUG00000022415	Inf	Inf	FAM162B	Up
ENSOCUG00000009825	4.201658326	2.070958849	BPHL	Up
ENSOCUG00000005792	3.881537802	1.956628338	ARL3	Up
ENSOCUG00000005897	3.905880278	1.965647729	B4GALT2	Up
ENSOCUG00000001429	5.290125862	2.403302047	MAOA	Up
ENSOCUG00000027040	4.326807413	2.113302908	LOXL3	Up
ENSOCUG00000012783	4.58652763	2.197402331	DAGLA	Up
ENSOCUG00000002818	5.679458484	2.50575338	PIMREG	Up
ENSOCUG00000012556	7.635400947	2.932703917	NFATC2	Up
ENSOCUG00000027243	3.885781563	1.958204803	-	Up
ENSOCUG00000002869	4.929563571	2.301459926	REC8	Up
ENSOCUG00000023860	25.22597199	4.656837954	ADRA2B	Up
ENSOCUG00000009431	4.408105446	2.140158735	NCALD	Up
ENSOCUG00000012799	8.82836223	3.142145825	SLC17A7	Up
ENSOCUG00000013251	5.027367877	2.329803262	STAC3	Up
ENSOCUG00000026819	5.777851721	2.53053318	-	Up
ENSOCUG00000004912	4.234238885	2.082102665	GNAZ	Up
ENSOCUG00000008553	7.754081564	2.954955911	PRUNE2	Up
ENSOCUG00000003091	3.794586739	1.923942773	ADCY7	Up
ENSOCUG00000025091	9.228235284	3.206054788	AARD	Up
ENSOCUG00000005539	3.756361713	1.909335992	NLGN2	Up
ENSOCUG00000004456	3.633747008	1.861457979	MAGED2	Up
ENSOCUG00000015419	4.567574608	2.191428294	NEIL2	Up
ENSOCUG00000026185	7.059504648	2.819566956	RBM43	Up

ENSOCUG00000012738	8.568739782	3.099083041	JAZF1	Up
ENSOCUG00000021438	4.650373401	2.217346562	ADGRE5	Up
ENSOCUG00000007605	4.292053488	2.101668055	CCDC28B	Up
ENSOCUG00000012883	5.418639363	2.437930632	-	Up
ENSOCUG00000024751	3.939431147	1.97798732	-	Up
ENSOCUG00000015089	4.293931695	2.102299242	TAMALIN	Up
ENSOCUG00000026532	13.69101265	3.775157254	SCARA5	Up
ENSOCUG00000012654	4.413559651	2.141942696	PREX1	Up
ENSOCUG00000012157	9.144207741	3.192858179	RSAD2	Up
ENSOCUG00000012309	4.992071891	2.319638711	SGCE	Up
ENSOCUG00000017871	4.937617476	2.303815073	ACTN2	Up
ENSOCUG00000026658	4.044206728	2.015856746	ADAMTS10	Up
ENSOCUG00000026764	3.653122511	1.869130137	-	Up
ENSOCUG00000008439	3.743201367	1.904272661	EGR1	Up
ENSOCUG00000013676	3.625281584	1.858093057	GSN	Up
ENSOCUG00000024298	3.759469692	1.910529171	NINJ1	Up
ENSOCUG00000001802	3.947388041	1.980898348	COPZ2	Up
ENSOCUG00000010709	6.00641012	2.586502986	SPAG4	Up
ENSOCUG00000016002	3.606374048	1.850549039	DPYSL2	Up
ENSOCUG00000014499	9.507263259	3.24903011	CLSTN3	Up
ENSOCUG00000026515	45.61229908	5.511350986	CXCL11	Up
ENSOCUG00000013771	3.968422182	1.988565515	UNC119	Up
ENSOCUG00000006636	4.212012118	2.074509587	PECR	Up
ENSOCUG00000003399	3.759280613	1.91045661	HMGN3	Up
ENSOCUG00000016897	3.978869277	1.992358501	SLC22A17	Up
ENSOCUG00000003519	4.689407082	2.229405523	ARHGAP44	Up
ENSOCUG00000026208	3.733805766	1.90064688	-	Up
ENSOCUG00000015838	6.142675124	2.618867084	COL9A2	Up
ENSOCUG00000001913	4.493507358	2.167841965	LDHD	Up
ENSOCUG00000015430	6.010750187	2.587545061	TRIM72	Up
ENSOCUG00000015547	4.443499102	2.151696197	LIMCH1	Up
ENSOCUG00000010632	6.758861624	2.756780278	NPAS3	Up
ENSOCUG00000004997	5.507924521	2.461508789	MST1	Up
ENSOCUG00000005115	3.841689107	1.941740772	FAP	Up
ENSOCUG00000004222	36.33505919	5.183290351	SLC22A3	Up
ENSOCUG00000008579	3.711421517	1.891971861	VASH2	Up
ENSOCUG00000014820	5.615386198	2.489385247	CYBRD1	Up
ENSOCUG00000012259	7.679735705	2.941056662	CMKLR1	Up
ENSOCUG00000007394	3.706811775	1.89017886	ADCY6	Up
ENSOCUG00000027941	8.117483826	3.021032605	KCNMB4	Up
ENSOCUG00000003347	3.636354986	1.862493044	SEMA4C	Up
ENSOCUG00000004160	6.770464421	2.759254799	FAM107A	Up
ENSOCUG00000009667	8.308636943	3.054611818	GABRE	Up
ENSOCUG00000028025	4.150892981	2.053421737	-	Up

ENSOCUG00000011871	3.870399409	1.952482454	KIAA1549	Up
ENSOCUG00000012459	3.662002749	1.872632874	FHL3	Up
ENSOCUG00000029364	4.012754937	2.004593052	IP6K3	Up
ENSOCUG00000001479	3.54227029	1.8246743	MAGED1	Up
ENSOCUG00000003594	3.477587167	1.798086677	OLFML2B	Up
ENSOCUG00000026348	11.29627703	3.49777547	KCNIP2	Up
ENSOCUG00000001337	3.738604395	1.902499819	PARM1	Up
ENSOCUG00000002777	3.680312495	1.87982827	-	Up
ENSOCUG00000012088	5.0560357	2.33800665	EMX2	Up
ENSOCUG00000000861	3.964502825	1.987139954	CD82	Up
ENSOCUG00000008029	20.78983658	4.377806513	ADRA1D	Up
ENSOCUG00000013289	8.954052858	3.162540835	PACRG	Up
ENSOCUG00000005561	3.665547281	1.874028615	TMEM9	Up
ENSOCUG00000000153	3.756386049	1.909345338	AKR7L	Up
ENSOCUG00000007829	10.10749886	3.337354136	SLC17A9	Up
ENSOCUG00000008011	20.46464152	4.35506149	ABCA8	Up
ENSOCUG00000012404	4.27838123	2.097065041	TMEM38A	Up
ENSOCUG00000009515	4.665352189	2.221985995	POPDC2	Up
ENSOCUG00000010870	5.881308193	2.556137093	CLMN	Up
ENSOCUG00000006716	4.003467244	2.001250002	GPR173	Up
ENSOCUG00000024196	4.431118389	2.147670873	-	Up
ENSOCUG00000006784	3.530263927	1.819776045	SH3PXD2B	Up
ENSOCUG00000009722	3.588319181	1.843308224	AHCYL1	Up
ENSOCUG00000003174	3.54691474	1.826564651	FARP1	Up
ENSOCUG00000027078	4.874687666	2.285309785	-	Up
ENSOCUG00000023000	3.664508303	1.873619633	ARHGEF19	Up
ENSOCUG00000014122	3.474642093	1.79686438	TPM1	Up
ENSOCUG00000000536	10.46470154	3.387459261	IRF8	Up
ENSOCUG00000007489	3.785914279	1.920641746	SHISA4	Up
ENSOCUG00000011207	3.992480049	1.997285198	MAGEH1	Up
ENSOCUG00000029623	6.875605576	2.781486786	ATP2B3	Up
ENSOCUG00000002748	3.592204165	1.844869349	S100A13	Up
ENSOCUG00000024492	4.955559776	2.309048032	ETV7	Up
ENSOCUG00000015145	4.408131285	2.140167192	NFATC4	Up
ENSOCUG00000025067	6.627698819	2.728508044	TMEM217	Up
ENSOCUG00000016686	3.513195328	1.81278379	-	Up
ENSOCUG00000022645	Inf	Inf	-	Up
ENSOCUG00000025012	5.695764597	2.509889522	ANGPT4	Up
ENSOCUG00000009739	3.457510072	1.789733454	P4HA1	Up
ENSOCUG00000013482	6.805111332	2.766618765	-	Up
ENSOCUG00000011353	4.537478841	2.181890916	INHA	Up
ENSOCUG00000008587	4.425484917	2.145835545	TNFSF4	Up
ENSOCUG00000004728	3.592971285	1.845177405	SHFL	Up
ENSOCUG00000006678	9.866704179	3.302568255	JAM2	Up

ENSOCUG00000015162	7.12933778	2.833768076	MFAP4	Up
ENSOCUG00000011745	3.52131914	1.816115986	HSPA2	Up
ENSOCUG00000016341	14.7360126	3.881274295	RAB37	Up
ENSOCUG00000010379	4.183525527	2.064719238	FREM1	Up
ENSOCUG00000012326	3.83340804	1.938627571	IL18BP	Up
ENSOCUG00000000310	27.66967076	4.790233573	IGDCC3	Up
ENSOCUG00000001500	3.531911502	1.820449194	-	Up
ENSOCUG00000013420	4.340666067	2.117916438	ADGRA2	Up
ENSOCUG00000025952	3.881106175	1.956467901	CELSR3	Up
ENSOCUG00000012557	16.67131232	4.059295769	SOX6	Up
ENSOCUG00000013427	4.557937492	2.188381139	DPEP2	Up
ENSOCUG00000013078	3.893444677	1.961047127	FAXDC2	Up
ENSOCUG00000008543	6.121749304	2.613943965	TNN	Up
ENSOCUG00000002945	6.026489865	2.591317948	MEOX1	Up
ENSOCUG00000015344	3.62063902	1.856244346	HEBP1	Up
ENSOCUG00000009340	3.535499151	1.821913913	ARAP3	Up
ENSOCUG00000022575	14.98224281	3.905181703	-	Up
ENSOCUG00000000901	11.73888284	3.553223212	SEZ6L2	Up
ENSOCUG00000016229	3.740032025	1.903050624	RABEPK	Up
ENSOCUG00000001799	3.467255296	1.793794067	EPB41L2	Up
ENSOCUG00000025121	3.620016266	1.85599618	ALDH6A1	Up
ENSOCUG00000008433	5.355468621	2.421012821	REEP2	Up
ENSOCUG00000005330	3.653594086	1.86931636	ACADSB	Up
ENSOCUG00000004542	10.89957237	3.446199629	RIMBP2	Up
ENSOCUG00000004656	4.45848123	2.156552344	DOK1	Up
ENSOCUG00000005929	3.486943194	1.801962861	MRAS	Up
ENSOCUG00000026154	3.853387879	1.946127414	PLPPR2	Up
ENSOCUG00000006539	3.341518057	1.74050367	-	Up
ENSOCUG00000017242	3.434768856	1.780213016	PDGFC	Up
ENSOCUG00000013259	3.400184141	1.765612879	-	Up
ENSOCUG00000011593	4.161829441	2.057217842	DCP1B	Up
ENSOCUG00000027251	6.305331282	2.656572172	C1orf54	Up
ENSOCUG00000013802	3.908201918	1.966505006	ALDOC	Up
ENSOCUG00000015527	3.650110702	1.867940219	PYGL	Up
ENSOCUG00000009150	24.89002752	4.637495921	TSKS	Up
ENSOCUG00000003250	3.600383875	1.848150736	NDN	Up
ENSOCUG00000004023	3.62229401	1.85690365	G0S2	Up
ENSOCUG00000026677	3.388877023	1.760807285	-	Up
ENSOCUG00000000960	4.77910472	2.25674038	SDK2	Up
ENSOCUG00000001999	3.271195242	1.70981787	GOLM1	Up
ENSOCUG00000003217	5.701748422	2.511404385	COL23A1	Up
ENSOCUG00000022267	3.596838058	1.846729206	DDAH2	Up
ENSOCUG00000027617	6.004502856	2.586044803	-	Up
ENSOCUG00000026164	3.742433391	1.903976639	CAMK1	Up

ENSOCUG00000002200	3.916418918	1.969535091	HDDC3	Up
ENSOCUG00000005206	4.006881213	2.002479741	SEC16B	Up
ENSOCUG00000009918	3.34642445	1.742620444	SCARA3	Up
ENSOCUG00000005428	4.10945027	2.038945414	ALOX5	Up
ENSOCUG00000021801	3.524075347	1.81724477	EML2	Up
ENSOCUG00000008182	3.573409751	1.837301353	CCDC136	Up
ENSOCUG00000010001	5.212660752	2.38201997	HSF4	Up
ENSOCUG00000020995	3.570913906	1.836293351	HSD17B8	Up
ENSOCUG00000011137	3.434530622	1.780112948	FBLN1	Up
ENSOCUG00000021262	3.544706255	1.825666078	USP35	Up
ENSOCUG00000010678	3.770768838	1.914858711	NOG	Up
ENSOCUG00000015784	5.232322007	2.38745133	FYN	Up
ENSOCUG00000014108	3.351918598	1.744987113	-	Up
ENSOCUG00000024150	9.291607736	3.215928249	SYN1	Up
ENSOCUG00000016689	3.503229675	1.808685576	-	Up
ENSOCUG00000014246	3.982482457	1.993668007	SORD	Up
ENSOCUG00000009574	3.444349781	1.784231659	-	Up
ENSOCUG00000002313	4.161191183	2.056996574	SEC14L2	Up
ENSOCUG00000033404	Inf	Inf	-	Up
ENSOCUG00000011097	3.458153152	1.790001763	BMP4	Up
ENSOCUG00000017117	3.295849807	1.7206505	B2M	Up
ENSOCUG00000004005	3.401293518	1.76608351	-	Up
ENSOCUG00000006651	5.815202801	2.539829506	-	Up
ENSOCUG00000010192	17.62145331	4.139261009	CDH5	Up
ENSOCUG00000027671	4.219586398	2.077101594	-	Up
ENSOCUG00000004048	Inf	Inf	WNT3A	Up
ENSOCUG00000002859	3.543144168	1.825030169	ADAMTS5	Up
ENSOCUG00000027610	5.731405887	2.518889069	SOX11	Up
ENSOCUG00000002320	3.421210944	1.77450706	PIK3IP1	Up
ENSOCUG00000016929	3.919229259	1.970569967	APBB1	Up
ENSOCUG00000024541	8.848228644	3.145388666	CHRNG	Up
ENSOCUG00000007503	5.285039621	2.401914287	-	Up
ENSOCUG00000010861	4.257056022	2.089856076	PEX11G	Up
ENSOCUG00000025635	3.466136944	1.793328655	CAMKK2	Up
ENSOCUG00000025473	3.405158912	1.767722128	-	Up
ENSOCUG00000007068	19.91074321	4.315475168	CNBD2	Up
ENSOCUG00000017588	3.686503412	1.882253092	KLHDC8B	Up
ENSOCUG00000017195	4.561620356	2.189546382	SEL1L3	Up
ENSOCUG00000016205	4.40189179	2.13812368	HOMER2	Up
ENSOCUG00000023919	3.826611081	1.936067279	-	Up
ENSOCUG00000014862	3.417393305	1.772896296	BIN1	Up
ENSOCUG00000026120	5.271619129	2.39824614	FOLH1	Up
ENSOCUG00000017885	6.93307861	2.793496119	MUSK	Up
ENSOCUG00000010338	3.488089718	1.802437148	-	Up

ENSOCUG00000001894	3.311647298	1.727549029	LFNG	Up
ENSOCUG00000002020	6.213851156	2.635487684	LGI3	Up
ENSOCUG00000014693	38.42634412	5.26402382	PGR	Up
ENSOCUG00000000479	3.366657734	1.751317059	-	Up
ENSOCUG00000014694	3.550947748	1.828204131	BCAS4	Up
ENSOCUG00000023956	5.450524964	2.446395189	-	Up
ENSOCUG00000005628	4.01861858	2.006699653	JAM3	Up
ENSOCUG00000010449	3.195850545	1.676199942	ADORA2B	Up
ENSOCUG00000008355	3.444240352	1.784185823	ARSA	Up
ENSOCUG00000029709	4.235099289	2.082395793	AKR7A3	Up
ENSOCUG00000008443	3.784344398	1.920043389	DCLK2	Up
ENSOCUG00000016704	4.454142826	2.15514782	GXYLT2	Up
ENSOCUG00000010187	3.376822442	1.755666323	CA9	Up
ENSOCUG00000026669	3.528602488	1.819096913	IAH1	Up
ENSOCUG00000008341	5.85487262	2.549637783	-	Up
ENSOCUG00000037644	Inf	Inf	PRCD	Up
ENSOCUG00000001297	3.512048682	1.812312843	PLEKHG5	Up
ENSOCUG00000014063	4.325493412	2.112864711	-	Up
ENSOCUG00000010126	3.129471483	1.64591903	ST3GAL5	Up
ENSOCUG00000011686	4.096534459	2.034403951	SLC38A3	Up
ENSOCUG00000015619	5.245839398	2.39117364	CALB2	Up
ENSOCUG00000006109	6.079604196	2.603977402	CNRIP1	Up
ENSOCUG00000013047	3.217927585	1.686131861	LAMC1	Up
ENSOCUG00000008144	3.450488083	1.78680045	CHCHD6	Up
ENSOCUG00000014272	10.1170875	3.338722123	IL19	Up
ENSOCUG00000023110	3.437962382	1.781553759	-	Up
ENSOCUG00000004768	3.801831272	1.926694505	MYORG	Up
ENSOCUG00000004995	4.339420077	2.117502253	PYROXD2	Up
ENSOCUG00000003676	44.26656332	5.468145468	SLIT1	Up
ENSOCUG00000015275	3.343506176	1.741361782	-	Up
ENSOCUG00000025186	5.8395498	2.545857149	-	Up
ENSOCUG00000030349	3.920621168	1.971082247	-	Up
ENSOCUG00000008865	10.78825168	3.431389178	THSD7A	Up
ENSOCUG00000016231	7.620161153	2.929821508	SLC16A5	Up
ENSOCUG00000014345	11.27971398	3.495658581	NHSL2	Up
ENSOCUG00000014239	4.69420188	2.230879888	TLCD4	Up
ENSOCUG00000003846	5.924667201	2.566734117	TRAM1L1	Up
ENSOCUG00000025624	11.21297968	3.487097798	GRIN2D	Up
ENSOCUG00000010984	3.601492945	1.848595078	-	Up
ENSOCUG00000033151	6.916933261	2.790132537	AOC3	Up
ENSOCUG00000007799	3.360069563	1.748491101	HOGA1	Up
ENSOCUG00000017680	3.447781574	1.785668379	INKA2	Up
ENSOCUG00000008627	4.603609455	2.202765448	SNCAIP	Up
ENSOCUG00000003573	3.252887855	1.701721085	TTYH3	Up

ENSOCUG00000005422	3.427972972	1.777355735	SNAI1	Up
ENSOCUG00000011697	19.19990906	4.263027573	VCAM1	Up
ENSOCUG00000008193	5.099512728	2.3503594	ACTA2	Up
ENSOCUG00000002349	4.519710119	2.176230245	CFAP45	Up
ENSOCUG00000013436	3.613773299	1.853506005	NHS	Up
ENSOCUG00000022649	5.13594167	2.360628818	APLN	Up
ENSOCUG00000008591	4.109077352	2.038814489	ELAPOR1	Up
ENSOCUG00000016140	20.68598635	4.370581844	TBXAS1	Up
ENSOCUG00000009504	5.047578033	2.335591309	CD80	Up
ENSOCUG00000024763	3.265962579	1.707508261	TREX1	Up
ENSOCUG00000005737	3.178014651	1.668125776	LGMN	Up
ENSOCUG00000001543	12.0044441	3.585496692	FAM13C	Up
ENSOCUG00000010668	3.149444363	1.655097325	FKBP9	Up
ENSOCUG00000001457	Inf	Inf	MYF6	Up
ENSOCUG00000026600	Inf	Inf	ASIC4	Up
ENSOCUG00000003712	5.325054185	2.412796205	TCEAL9	Up
ENSOCUG00000000157	4.389582257	2.134083649	-	Up
ENSOCUG00000013452	24.54123302	4.617135831	PRTFDC1	Up
ENSOCUG00000004091	3.266128004	1.707581333	EPB41L1	Up
ENSOCUG00000004115	3.275451719	1.711693883	FCSK	Up
ENSOCUG00000008485	3.243223278	1.697428349	-	Up
ENSOCUG00000010729	4.381742159	2.131504592	RASL12	Up
ENSOCUG00000008675	12.68492119	3.66504265	-	Up
ENSOCUG00000024398	3.175732842	1.667089551	IFNAR2	Up
ENSOCUG00000001828	3.163315037	1.661437242	CERK	Up
ENSOCUG00000006357	12.92030139	3.691567819	ADAMTS12	Up
ENSOCUG00000001107	9.200923304	3.201778641	ARHGAP4	Up
ENSOCUG00000015769	3.633666639	1.86142607	RADIL	Up
ENSOCUG00000003801	3.22140098	1.687688249	C1QTNF1	Up
ENSOCUG00000011085	11.70947511	3.549604502	CTSS	Up
ENSOCUG00000010063	3.243236971	1.69743444	LRRC49	Up
ENSOCUG00000009792	3.182309511	1.670074159	NUMBL	Up
ENSOCUG00000015113	3.125694211	1.644176645	MTSS2	Up
ENSOCUG00000011300	3.328108763	1.734702582	CYP4V2	Up
ENSOCUG00000023727	3.596190277	1.846469357	LCAT	Up
ENSOCUG00000002688	4.970335828	2.313343333	ATP2A1	Up
ENSOCUG00000008307	7.714197587	2.947516099	-	Up
ENSOCUG00000000111	3.724627694	1.897096224	COL4A3	Up
ENSOCUG00000002606	5.983480495	2.580984921	HES7	Up
ENSOCUG00000013587	3.675561364	1.877964608	IL10RA	Up
ENSOCUG00000025401	4.066457727	2.023772616	PPP1R35	Up
ENSOCUG00000023764	3.887248595	1.958749373	CCDC80	Up
ENSOCUG00000006107	5.713671809	2.514418171	GRAMD2A	Up
ENSOCUG00000006938	17.78568276	4.152644453	CSDC2	Up

ENSOCUG00000026915	5.281962695	2.401074112	PTGIR	Up
ENSOCUG00000000596	3.219610931	1.686886359	PLAAT3	Up
ENSOCUG00000024679	3.225791019	1.689652977	FKBP7	Up
ENSOCUG00000016506	3.325053029	1.733377349	PLXNB3	Up
ENSOCUG00000001725	4.45982188	2.156986092	PDGFRB	Up
ENSOCUG00000026630	5.016368684	2.326643385	-	Up
ENSOCUG00000008163	3.225681004	1.689603774	TAPBPL	Up
ENSOCUG00000011141	3.379398046	1.75676629	RET	Up
ENSOCUG00000000435	3.994651828	1.998069764	DAPK2	Up
ENSOCUG00000008065	3.181416414	1.669669218	NBL1	Up
ENSOCUG00000001836	3.213520452	1.684154654	ADGRL1	Up
ENSOCUG00000006534	11.78394424	3.558750604	-	Up
ENSOCUG00000001897	3.248048268	1.699573072	HEXD	Up
ENSOCUG00000014363	3.48191061	1.799879166	PCNX2	Up
ENSOCUG00000010062	4.123646623	2.043920706	DYSF	Up
ENSOCUG00000002613	6.228850653	2.638965982	VCAN	Up
ENSOCUG00000013097	3.223424976	1.688594406	C14orf93	Up
ENSOCUG00000005816	3.11923167	1.641190708	SORBS2	Up
ENSOCUG00000005347	4.921582363	2.299122238	PIF1	Up
ENSOCUG00000002344	3.120956957	1.64198846	SEMA3B	Up
ENSOCUG00000017907	10.18333412	3.348138086	TMEM37	Up
ENSOCUG00000013393	4.0547361	2.019608021	ZC4H2	Up
ENSOCUG00000006085	9.823539656	3.296242956	BBOX1	Up
ENSOCUG00000007510	3.083508772	1.62457295	LRIG1	Up
ENSOCUG00000011961	16.6744111	4.059563905	TMEM35A	Up
ENSOCUG00000002177	4.229462604	2.080474366	-	Up
ENSOCUG00000021601	3.116525547	1.639938539	SCARB1	Up
ENSOCUG00000001759	3.047389637	1.607573974	CDCA7	Up
ENSOCUG00000002478	22.19239918	4.471993738	-	Up
ENSOCUG00000012053	3.092855417	1.628939393	PPIC	Up
ENSOCUG00000009621	8.068995481	3.012389082	HSF2BP	Up
ENSOCUG00000011549	2.998235156	1.584113541	SPATS2L	Up
ENSOCUG00000015020	4.032458084	2.011659537	COL5A2	Up
ENSOCUG00000016236	5.487228188	2.456077572	COL15A1	Up
ENSOCUG00000027853	7.8253089	2.968147703	PGAM2	Up
ENSOCUG00000031804	5.988685697	2.582239418	-	Up
ENSOCUG00000007870	6.798154442	2.765143138	SCN5A	Up
ENSOCUG00000003118	4.495991802	2.168639405	SLC12A5	Up
ENSOCUG00000033850	7.482623989	2.90354428	-	Up
ENSOCUG00000022123	11.13623515	3.477189675	-	Up
ENSOCUG00000001748	3.518104093	1.81479817	TMTC1	Up
ENSOCUG00000025803	6.308153106	2.657217676	-	Up
ENSOCUG00000027106	3.683189249	1.880955528	HAS2	Up
ENSOCUG00000024732	3.421531571	1.774642259	TMEM143	Up

ENSOCUG00000016552	3.411470346	1.770393676	TSPAN5	Up
ENSOCUG00000013739	3.263527952	1.706432396	FNDC4	Up
ENSOCUG00000015623	3.064670052	1.615731759	PLXNA3	Up
ENSOCUG00000030668	5.075223022	2.343471221	-	Up
ENSOCUG00000002483	3.62893412	1.859545866	NKIRAS1	Up
ENSOCUG00000025690	Inf	Inf	ART5	Up
ENSOCUG00000013131	5.784019249	2.532072353	MYOM2	Up
ENSOCUG00000000637	3.720792165	1.895609807	MYL9	Up
ENSOCUG00000012443	8.181515999	3.032368193	FAM189A1	Up
ENSOCUG00000025172	5.152818107	2.365361667	KCNK3	Up
ENSOCUG00000006028	6.167203515	2.624616455	NEURL3	Up
ENSOCUG00000004165	2.954565605	1.562946034	IRF1	Up
ENSOCUG00000010565	11.72189987	3.551134514	-	Up
ENSOCUG00000006015	4.304077072	2.105703912	HLX	Up
ENSOCUG00000006133	4.406049171	2.139485595	PLA2G6	Up
ENSOCUG00000010061	10.56013687	3.400556629	PLEKHG4	Up
ENSOCUG00000011267	4.286463183	2.099787752	-	Up
ENSOCUG00000015301	3.55747528	1.830853732	SCN1B	Up
ENSOCUG00000007959	3.266637607	1.707806414	PARD3B	Up
ENSOCUG00000003227	60.07919802	5.90879365	-	Up
ENSOCUG00000009441	3.05362151	1.610521254	DZIP1	Up
ENSOCUG00000017194	23.44369099	4.551127822	-	Up
ENSOCUG00000024945	Inf	Inf	-	Up
ENSOCUG00000003348	3.237954157	1.69508256	DMAC2L	Up
ENSOCUG00000009521	3.065796845	1.6162621	TCEAL8	Up
ENSOCUG00000011332	8.786769833	3.135332903	PACSIN1	Up
ENSOCUG00000014263	8.39949457	3.070302518	BATF2	Up
ENSOCUG00000028226	11.45011868	3.517290646	PABPC4L	Up
ENSOCUG00000024772	3.027770023	1.598255628	-	Up
ENSOCUG00000025655	3.226818262	1.690112326	GPR161	Up
ENSOCUG00000020994	3.051131751	1.609344478	ST6GALNAC4	Up
ENSOCUG00000006120	27.33637365	4.772749967	RGR	Up
ENSOCUG00000003749	5.715164652	2.514795063	PIANP	Up
ENSOCUG00000007445	20.11379839	4.330113648	-	Up
ENSOCUG00000029638	7.998729382	2.999770842	ANKRD34A	Up
ENSOCUG00000004107	3.066337552	1.616516522	ST3GAL2	Up
ENSOCUG00000003063	3.075364124	1.620757236	ALDH5A1	Up
ENSOCUG00000004288	3.299317375	1.722167563	LIMD2	Up
ENSOCUG00000011556	3.149305151	1.655033554	KCTD18	Up
ENSOCUG00000017565	4.940675355	2.304708261	KCP	Up
ENSOCUG00000026917	3.10453861	1.634378874	-	Up
ENSOCUG00000005101	2.940817759	1.556217384	LAMB2	Up
ENSOCUG00000012094	3.361392925	1.749059194	TRAM2	Up
ENSOCUG00000024962	3.240711491	1.696310588	-	Up

ENSOCUG00000004377	4.781756601	2.257540697	-	Up
ENSOCUG00000021745	3.59666297	1.846658976	LHFPL2	Up
ENSOCUG00000014497	9.282261344	3.214476318	EFHD1	Up
ENSOCUG00000002572	3.279229175	1.713356731	LRRC3	Up
ENSOCUG00000015498	4.22217608	2.077986746	EFCAB11	Up
ENSOCUG00000008651	8.812377808	3.139531348	ITM2A	Up
ENSOCUG00000012683	5.093052169	2.348530495	ADAM33	Up
ENSOCUG00000038697	21.59306554	4.43249617	-	Up
ENSOCUG00000009793	3.056074073	1.611679512	RUSF1	Up
ENSOCUG00000024791	2.971776902	1.571325814	SLC25A1	Up
ENSOCUG00000002852	3.251192648	1.700969045	EMC9	Up
ENSOCUG00000012396	2.987511995	1.578944506	ZMYM3	Up
ENSOCUG00000000911	3.213882408	1.684317144	TBC1D16	Up
ENSOCUG00000031846	10.73055362	3.423652606	-	Up
ENSOCUG00000009285	41.07593837	5.360221628	C11orf53	Up
ENSOCUG00000004928	4.542902303	2.18361428	TGFBI	Up
ENSOCUG00000003018	3.851680947	1.945488203	LIX1L	Up
ENSOCUG00000007886	4.078188957	2.027928622	CYRIA	Up
ENSOCUG00000013577	3.324511812	1.733142504	-	Up
ENSOCUG00000004334	4.310962189	2.108009909	TMEM255A	Up
ENSOCUG00000008966	2.886607994	1.529375201	P4HA2	Up
ENSOCUG00000011228	2.942113748	1.556853025	PDE4DIP	Up
ENSOCUG00000007720	4.761404013	2.251387049	QPRT	Up
ENSOCUG00000022357	3.206861971	1.681162259	PDK2	Up
ENSOCUG00000000191	9.597615428	3.262676006	YPEL4	Up
ENSOCUG00000021126	3.032820572	1.600660148	-	Up
ENSOCUG00000007629	2.936985578	1.554336182	P3H4	Up
ENSOCUG00000016402	5.266905182	2.396955487	CARMIL3	Up
ENSOCUG00000021867	4.458440968	2.156539315	UBE2L6	Up
ENSOCUG00000034678	3.470835749	1.795283094	ARMCX6	Up
ENSOCUG00000014622	2.979640544	1.575138298	-	Up
ENSOCUG00000014102	3.02609989	1.597459611	JCAD	Up
ENSOCUG00000023383	3.1829959	1.670385299	-	Up
ENSOCUG00000013987	3.411865627	1.770560829	FGF1	Up
ENSOCUG00000008749	3.050970076	1.60926803	TCF7L1	Up
ENSOCUG00000027152	4.231881707	2.081299301	-	Up
ENSOCUG00000026304	8.772625612	3.1330087	ECRG4	Up
ENSOCUG00000009839	3.176833332	1.667589403	HDAC11	Up
ENSOCUG00000014724	3.11735735	1.640323544	CMTM7	Up
ENSOCUG00000010579	5.002570118	2.322669484	ABAT	Up
ENSOCUG00000027207	3.42150562	1.774631317	FANCE	Up
ENSOCUG00000023346	4.885158057	2.288405241	ISG20	Up
ENSOCUG00000011863	3.077581609	1.621797114	HDDC2	Up
ENSOCUG00000007913	3.058229377	1.612696617	ZNF862	Up

ENSOCUG00000002323	3.096091156	1.630447949	PATZ1	Up
ENSOCUG00000005982	3.000266665	1.585090734	B4GALT5	Up
ENSOCUG00000014121	Inf	Inf	B4GALNT1	Up
ENSOCUG00000024383	2.874270686	1.523195935	MIF	Up
ENSOCUG00000003633	3.025272424	1.597065062	GFRA1	Up
ENSOCUG00000006104	9.639199576	3.268913352	CFAP300	Up
ENSOCUG00000013478	2.878300309	1.525217124	HTRA1	Up
ENSOCUG00000005758	3.061917158	1.61443525	ARL4C	Up
ENSOCUG00000001241	3.996580185	1.998766035	MFSD4A	Up
ENSOCUG00000026440	2.987338575	1.578860757	-	Up
ENSOCUG00000011881	3.45574248	1.788995713	RGS7	Up
ENSOCUG00000008670	5.978200953	2.579711393	SSTR1	Up
ENSOCUG00000014873	2.893111868	1.532622109	-	Up
ENSOCUG00000001254	3.108978298	1.636440546	CLTCL1	Up
ENSOCUG00000029212	3.134703875	1.648329163	-	Up
ENSOCUG00000003523	8.917761517	3.156681619	SYT6	Up
ENSOCUG00000006763	2.97638908	1.573563131	-	Up
ENSOCUG00000014939	3.182613754	1.67021208	PCED1A	Up
ENSOCUG00000021091	3.068648584	1.617603441	SLC5A2	Up
ENSOCUG00000024974	3.207534869	1.681464949	NT5C	Up
ENSOCUG00000003299	3.483851894	1.800683293	DMRT2	Up
ENSOCUG00000007808	3.030549508	1.599579411	CAVIN3	Up
ENSOCUG00000010987	5.782359212	2.531658235	TMEM26	Up
ENSOCUG00000033836	3.215241934	1.684927299	HSD17B14	Up
ENSOCUG00000017533	3.033853332	1.601151342	CLN6	Up
ENSOCUG00000015812	2.968594648	1.569780111	PRPSAP2	Up
ENSOCUG00000002692	5.35908861	2.421987671	GRIK4	Up
ENSOCUG00000012530	4.362884943	2.125282428	NPNT	Up
ENSOCUG00000001137	3.128517913	1.645479365	LHFPL6	Up
ENSOCUG00000028091	4.151113432	2.053498355	THNSL2	Up
ENSOCUG00000016259	5.791417937	2.533916612	DDIT4L	Up
ENSOCUG00000014083	3.025937564	1.59738222	LZTS1	Up
ENSOCUG00000014657	3.175751507	1.66709803	EFEMP1	Up
ENSOCUG00000013016	5.011857154	2.325345296	MAPK4	Up
ENSOCUG00000000798	3.6628223	1.872955711	H6PD	Up
ENSOCUG00000005192	4.418165649	2.14344751	TLCD5	Up
ENSOCUG00000000148	3.788965975	1.921804185	CDO1	Up
ENSOCUG00000022982	3.084147011	1.624871535	D2HGDH	Up
ENSOCUG00000013025	3.339033471	1.739430555	NAGS	Up
ENSOCUG00000024302	4.01045916	2.003767421	LGALS1	Up
ENSOCUG00000008772	35.78374296	5.161232395	FANK1	Up
ENSOCUG00000000259	4.356678048	2.123228504	-	Up
ENSOCUG00000012995	4.215180628	2.075594455	MERTK	Up
ENSOCUG00000009440	3.016267603	1.59276443	LRP3	Up

ENSOCUG00000008107	Inf	Inf	-	Up
ENSOCUG00000022428	3.877500033	1.955126793	POLM	Up
ENSOCUG00000025617	2.86237925	1.517214834	CDH13	Up
ENSOCUG00000007855	3.039200773	1.603691984	PXYLP1	Up
ENSOCUG00000011364	3.529145833	1.819319047	-	Up
ENSOCUG00000010446	2.965786502	1.568414746	ATG16L2	Up
ENSOCUG00000022291	5.508540136	2.461670029	-	Up
ENSOCUG00000009508	2.77434226	1.472145778	ADPRH	Up
ENSOCUG00000010198	3.208066223	1.681703923	Tpm2	Up
ENSOCUG00000016065	5.81470382	2.539705708	PITX3	Up
ENSOCUG00000002861	2.792545843	1.481580965	-	Up
ENSOCUG00000012645	3.142278095	1.651810866	MECR	Up
ENSOCUG00000007479	3.38477681	1.759060707	PDE3A	Up
ENSOCUG00000002118	3.182122942	1.669989576	GPR176	Up
ENSOCUG00000002254	2.749423773	1.459129289	DNMBP	Up
ENSOCUG00000010075	3.460028879	1.790784079	TRABD2A	Up
ENSOCUG00000021527	5.081125501	2.345148098	-	Up
ENSOCUG00000002760	3.360864493	1.748832376	HIP1	Up
ENSOCUG00000033676	2.848967629	1.510439229	SELENOM	Up
ENSOCUG00000028002	3.020278406	1.594681542	MDP1	Up
ENSOCUG00000002220	2.902899012	1.537494383	P2RX4	Up
ENSOCUG00000017737	20.31624468	4.344561849	P2RX2	Up
ENSOCUG00000005589	4.900863252	2.293035892	ZAN	Up
ENSOCUG00000008644	4.861560934	2.281419604	-	Up
ENSOCUG00000037987	3.984388184	1.99435821	-	Up
ENSOCUG00000005593	2.768418212	1.469061901	FXYD6	Up
ENSOCUG00000012397	2.774614545	1.472287363	TSC22D3	Up
ENSOCUG00000023580	4.104643376	2.037256882	ITPKA	Up
ENSOCUG00000017372	3.968990198	1.988771999	KLHL29	Up
ENSOCUG00000015477	7.514883048	2.909750652	-	Up
ENSOCUG00000026783	2.90779441	1.53992527	PCGF2	Up
ENSOCUG00000005457	3.102456166	1.633410827	SLC27A3	Up
ENSOCUG00000011193	2.906851651	1.539457447	SYTL4	Up
ENSOCUG00000030256	9.104044083	3.186507543	TET1	Up
ENSOCUG00000005145	3.534657743	1.821570527	EEPD1	Up
ENSOCUG00000002624	Inf	Inf	NRK	Up
ENSOCUG00000017891	2.794246035	1.482459056	SLC29A1	Up
ENSOCUG00000022140	4.172750531	2.060998671	SAMD14	Up
ENSOCUG00000030033	3.308365991	1.726118843	-	Up
ENSOCUG00000000612	18.45105945	4.205631753	CD101	Up
ENSOCUG00000025048	6.657627785	2.735008214	CXCR5	Up
ENSOCUG00000003436	4.541824857	2.183272074	SPTBN4	Up
ENSOCUG00000024266	3.78233517	1.919277213	PLEKHH2	Up
ENSOCUG00000008662	3.152187394	1.656353304	KHK	Up

ENSOCUG00000003499	3.056636725	1.611945101	FADS2	Up
ENSOCUG00000023813	2.924847915	1.54836161	-	Up
ENSOCUG00000016264	10.6887348	3.41801919	ENPEP	Up
ENSOCUG00000022621	4.979678266	2.316052534	KLHL33	Up
ENSOCUG00000005985	2.74092735	1.454664089	CLU	Up
ENSOCUG00000000249	4.231843302	2.081286208	SMARCD3	Up
ENSOCUG00000005650	3.583480489	1.841361499	FRAT1	Up
ENSOCUG00000034490	3.326000094	1.733788209	NREP	Up
ENSOCUG00000025954	7.218194831	2.851638084	INPP5D	Up
ENSOCUG00000003991	2.930845307	1.551316823	DZIP1L	Up
ENSOCUG00000009427	3.381108044	1.757496119	LAMA2	Up
ENSOCUG00000000431	4.754664119	2.249343429	CRABP1	Up
ENSOCUG00000022861	5.032982734	2.331413649	FBXO24	Up
ENSOCUG00000026037	3.379339171	1.756741155	GNB1L	Up
ENSOCUG00000016208	3.02556223	1.597203258	NAXE	Up
ENSOCUG00000015071	4.734162741	2.243109301	ALDH1L1	Up
ENSOCUG00000004122	2.778605809	1.474361179	NIPSNAP1	Up
ENSOCUG00000004710	2.782065493	1.476156383	-	Up
ENSOCUG00000013280	2.837015414	1.504373989	ARHGEF3	Up
ENSOCUG00000015845	2.787851065	1.47915349	RHOBTB3	Up
ENSOCUG00000012908	3.751005849	1.907277513	DNM1	Up
ENSOCUG00000008690	2.948599387	1.560029823	TMCO4	Up
ENSOCUG00000025890	3.756702284	1.909466788	-	Up
ENSOCUG00000001761	9.125564564	3.189913816	-	Up
ENSOCUG00000026610	10.5696866	3.401860695	TMEM100	Up
ENSOCUG00000016671	3.158972887	1.659455555	VANGL2	Up
ENSOCUG00000031627	2.92862678	1.55022435	-	Up
ENSOCUG00000016361	4.431623624	2.147835359	CD302	Up
ENSOCUG00000000371	3.832709475	1.938364644	TTC38	Up
ENSOCUG00000017020	2.842328736	1.507073422	SRD5A1	Up
ENSOCUG00000002250	3.24197691	1.696873816	GGT7	Up
ENSOCUG00000005088	2.759641602	1.464480914	-	Up
ENSOCUG00000005005	3.113145236	1.638372884	RFX2	Up
ENSOCUG00000002848	8.920436465	3.157114301	-	Up
ENSOCUG00000005660	3.058550293	1.612847999	PDGFA	Up
ENSOCUG00000009435	4.086576435	2.030892719	XKR5	Up
ENSOCUG00000011941	3.446350107	1.785069269	HVCN1	Up
ENSOCUG00000000109	3.457758454	1.789837091	ADAMTS9	Up
ENSOCUG00000007211	4.30184592	2.104955853	-	Up
ENSOCUG00000029210	2.992135235	1.581175382	AP5B1	Up
ENSOCUG00000033970	7.67728734	2.940596645	-	Up
ENSOCUG00000010761	6.987051646	2.804683804	NPY	Up
ENSOCUG00000000349	3.57617327	1.838416639	TRIM46	Up
ENSOCUG00000008272	4.569978164	2.192187272	SRPX	Up

ENSOCUG00000008000	3.318013866	1.730319915	B9D2	Up
ENSOCUG00000008953	4.171500755	2.060566507	SYNPO2	Up
ENSOCUG00000016445	3.640169365	1.864005576	-	Up
ENSOCUG00000021153	6.59350425	2.721045418	MAP3K7CL	Up
ENSOCUG00000014520	2.592652719	1.374428973	CAV1	Up
ENSOCUG00000011893	2.81921227	1.495292108	GNB5	Up
ENSOCUG00000006343	6.677703683	2.739352076	KLF9	Up
ENSOCUG00000025224	2.726765899	1.447190846	LIMK1	Up
ENSOCUG00000013700	2.725981403	1.44677572	CTSF	Up
ENSOCUG00000008183	3.443199017	1.783749571	HS6ST2	Up
ENSOCUG00000016170	2.735412305	1.451758305	PYGO2	Up
ENSOCUG00000013520	2.862606287	1.517329261	NR2F2	Up
ENSOCUG00000026468	3.023561212	1.596248787	BDKRB1	Up
ENSOCUG00000007382	3.986643442	1.995174579	INHBE	Up
ENSOCUG00000005228	4.695656122	2.231326759	NKD1	Up
ENSOCUG00000000553	3.736637803	1.901740728	TCEAL1	Up
ENSOCUG00000005747	4.235035147	2.082373943	COL16A1	Up
ENSOCUG00000000459	2.797532706	1.484154998	-	Up
ENSOCUG00000022727	3.031764003	1.600157457	-	Up
ENSOCUG00000012806	3.664078297	1.873450333	RGS14	Up
ENSOCUG00000014010	15.69132599	3.971895367	OR51E1	Up
ENSOCUG00000021051	18.37568169	4.199725866	RIPPLY3	Up
ENSOCUG00000010550	3.096898341	1.630824026	B3GALT4	Up
ENSOCUG00000002846	2.67154155	1.417672455	PSME1	Up
ENSOCUG00000010721	11.74870124	3.554429377	LRRC71	Up
ENSOCUG00000022195	Inf	Inf	-	Up
ENSOCUG00000012120	6.2545312	2.644901753	SPARC	Up
ENSOCUG00000017325	4.451893948	2.154419226	KCNAB3	Up
ENSOCUG00000023031	2.908191374	1.540122209	-	Up
ENSOCUG00000009849	2.759104494	1.464200096	GLT8D1	Up
ENSOCUG00000009421	28.65509124	4.840719585	-	Up
ENSOCUG00000016636	2.920024536	1.545980492	TMEM8B	Up
ENSOCUG00000006306	2.732754548	1.450355885	PINK1	Up
ENSOCUG00000014637	2.70843506	1.4374595	-	Up
ENSOCUG00000012456	2.758420535	1.46384242	ZNF362	Up
ENSOCUG00000014492	2.782046212	1.476146385	IRAK3	Up
ENSOCUG00000017857	3.752639713	1.907905785	BICD1	Up
ENSOCUG00000007900	2.878216453	1.525175093	RCOR2	Up
ENSOCUG00000027662	3.63449352	1.861754333	C16orf71	Up
ENSOCUG00000026867	2.869415302	1.52075679	LTK	Up
ENSOCUG00000012081	2.870195527	1.521149022	CACNB1	Up
ENSOCUG00000003626	11.83562129	3.565063534	KIAA2012	Up
ENSOCUG00000026221	3.783474367	1.919711671	-	Up
ENSOCUG00000001144	2.635409338	1.398027062	SLC6A6	Up

ENSOCUG00000024729	3.502445053	1.808362418	HSPB3	Up
ENSOCUG00000006524	3.039966839	1.604055586	BMF	Up
ENSOCUG00000000114	4.208989006	2.073473741	DUSP2	Up
ENSOCUG00000002875	16.831433	4.073086105	STOX1	Up
ENSOCUG00000012841	5.262926754	2.395865316	-	Up
ENSOCUG00000001963	2.908993313	1.54051998	TNS1	Up
ENSOCUG00000025982	2.645457443	1.403517209	-	Up
ENSOCUG00000013207	2.700460247	1.433205311	POU6F1	Up
ENSOCUG00000012353	8.344057993	3.060749185	SDSL	Up
ENSOCUG00000005605	2.957713549	1.564482336	LDLRAD4	Up
ENSOCUG00000005365	2.766812336	1.468224794	-	Up
ENSOCUG00000002563	2.834878613	1.503286962	-	Up
ENSOCUG00000016500	2.694614625	1.430078958	-	Up
ENSOCUG00000017021	6.593114929	2.72096023	-	Up
ENSOCUG00000010783	4.140515876	2.049810528	STARD8	Up
ENSOCUG00000034268	4.09081878	2.032389628	-	Up
ENSOCUG00000025336	2.621095887	1.390170132	PCBP4	Up
ENSOCUG00000022093	3.45149886	1.787223008	ACAN	Up
ENSOCUG00000006503	2.622783625	1.391098793	TBC1D2B	Up
ENSOCUG00000009277	3.003443525	1.586617537	RND2	Up
ENSOCUG00000013208	2.826913211	1.499227591	PRXL2C	Up
ENSOCUG00000026916	2.746969358	1.457840817	GTF2IRD1	Up
ENSOCUG00000000404	2.601178365	1.37916533	CABIN1	Up
ENSOCUG00000006348	2.921246394	1.546584048	PSMB10	Up
ENSOCUG00000016069	2.625008614	1.392322157	RNF144A	Up
ENSOCUG00000005798	4.974075383	2.314428372	GALNT14	Up
ENSOCUG00000027757	2.638734374	1.39984613	CMTM3	Up
ENSOCUG00000011879	6.587248026	2.719675872	KLHL31	Up
ENSOCUG00000011355	2.598689788	1.377784426	SCPEP1	Up
ENSOCUG00000009728	2.641001217	1.401084966	THRA	Up
ENSOCUG00000010021	4.063772898	2.02281978	CMPK2	Up
ENSOCUG00000023927	4.476230132	2.16228421	-	Up
ENSOCUG00000001933	2.76550516	1.467543034	PTPRJ	Up
ENSOCUG00000036279	3.152110992	1.656318336	-	Up
ENSOCUG00000003185	2.569856946	1.361688052	CSPG4	Up
ENSOCUG00000006316	2.659883156	1.411362872	PLEKHO1	Up
ENSOCUG00000021257	8.409227198	3.071973224	MATN3	Up
ENSOCUG00000012750	3.06262413	1.614768319	BRSK1	Up
ENSOCUG00000001465	3.815367319	1.931821957	PAPPA	Up
ENSOCUG00000012721	2.581790745	1.368372074	DAB2	Up
ENSOCUG00000014110	2.883808571	1.527975401	-	Up
ENSOCUG00000023020	2.812941492	1.492079546	-	Up
ENSOCUG00000021002	2.69329777	1.429373742	LCN2	Up
ENSOCUG00000011942	3.907597725	1.966281954	FLNC	Up

ENSOCUG00000003215	4.15032289	2.053223581	SPRY4	Up
ENSOCUG00000022585	3.037399232	1.602836547	PRKD1	Up
ENSOCUG00000011825	2.714207276	1.4405309	ZDHHC1	Up
ENSOCUG00000011582	3.312564971	1.727948751	MPP2	Up
ENSOCUG00000014001	Inf	Inf	CYYR1	Up
ENSOCUG00000007624	11.32553845	3.501507736	PRKCG	Up
ENSOCUG00000027310	2.52729125	1.337591933	SLC16A3	Up
ENSOCUG00000003905	2.709909571	1.43824471	CCN1	Up
ENSOCUG00000012465	2.633659935	1.397069073	TSPAN31	Up
ENSOCUG00000005447	2.558741177	1.355434223	DENND2B	Up
ENSOCUG00000021283	3.679291918	1.879428145	-	Up
ENSOCUG00000000865	2.927016632	1.549430943	ARSL	Up
ENSOCUG00000014601	2.817748387	1.494542791	-	Up
ENSOCUG00000011970	3.357705856	1.747475852	-	Up
ENSOCUG00000008769	3.138025269	1.64985697	PCDHAC2	Up
ENSOCUG00000009108	2.747807277	1.458280821	-	Up
ENSOCUG00000037070	6.014751866	2.588505221	-	Up
ENSOCUG00000017267	2.691950271	1.428651759	SLC37A4	Up
ENSOCUG00000012700	2.771124188	1.470471367	CORO6	Up
ENSOCUG00000016223	2.704076816	1.435136136	-	Up
ENSOCUG00000021012	3.167830702	1.663495236	-	Up
ENSOCUG00000008198	2.892301585	1.532217992	-	Up
ENSOCUG00000000804	3.07892785	1.622428061	SCRN1	Up
ENSOCUG00000002065	2.547327504	1.348984456	TCAF1	Up
ENSOCUG00000010232	3.956005584	1.984044463	ANTXR1	Up
ENSOCUG00000007657	2.745584131	1.457113119	FAM174A	Up
ENSOCUG00000024073	2.66428467	1.413748238	-	Up
ENSOCUG00000003689	3.390995759	1.76170898	NID2	Up
ENSOCUG00000016624	2.519358748	1.333056572	MYH10	Up
ENSOCUG00000023456	4.682800992	2.227371728	-	Up
ENSOCUG00000001428	2.628433955	1.394203485	PDE4A	Up
ENSOCUG00000038840	2.646094204	1.403864424	-	Up
ENSOCUG00000024530	5.373088254	2.425751535	APOC1	Up
ENSOCUG00000010547	3.416557989	1.772543614	HLF	Up
ENSOCUG00000021420	5.21001637	2.381287906	-	Up
ENSOCUG00000032092	4.789962917	2.260014487	-	Up
ENSOCUG00000005927	2.668834612	1.416209904	ACSM5	Up
ENSOCUG00000009174	14.53599859	3.86155828	CDHR4	Up
ENSOCUG00000002066	2.971416109	1.57115065	C11orf49	Up
ENSOCUG00000010871	3.391673934	1.76199748	DNALI1	Up
ENSOCUG00000000236	2.792582523	1.481599914	RGMA	Up
ENSOCUG00000012486	3.143949135	1.652577877	CYP27B1	Up
ENSOCUG00000008744	2.687850582	1.42645294	TMEM205	Up
ENSOCUG00000014089	2.621472708	1.390377526	PLD1	Up

ENSOCUG00000009881	2.923805394	1.54784729	EML1	Up
ENSOCUG00000014920	2.637408016	1.399120779	SPRY1	Up
ENSOCUG00000002287	3.245766661	1.698559288	-	Up
ENSOCUG00000024007	4.664080512	2.221592693	WNT6	Up
ENSOCUG00000009447	2.824871226	1.498185103	OSBPL7	Up
ENSOCUG00000027846	2.937681613	1.554678045	DNAJB5	Up
ENSOCUG00000014618	2.700923166	1.4334526	RGS10	Up
ENSOCUG00000004455	5.387603526	2.429643687	GUCY1A1	Up
ENSOCUG00000022079	9.599051357	3.262891836	ANKRD55	Up
ENSOCUG00000015713	2.602691031	1.380004058	TMEM132A	Up
ENSOCUG00000009751	5.755206843	2.52486778	HCK	Up
ENSOCUG00000029419	3.249336892	1.70014533	-	Up
ENSOCUG00000001360	4.764151585	2.252219317	GAB3	Up
ENSOCUG00000004558	3.324145927	1.732983716	PCDHB5	Up
ENSOCUG00000034254	4.209773043	2.073742457	ZSCAN2	Up
ENSOCUG00000027233	3.759403171	1.910503643	-	Up
ENSOCUG00000037568	31.07405842	4.957638772	-	Up
ENSOCUG00000007401	2.57737478	1.365902337	CACNB3	Up
ENSOCUG00000024203	3.120530342	1.641791239	SLC2A10	Up
ENSOCUG00000005640	3.649763918	1.867803147	-	Up
ENSOCUG00000002281	3.520177099	1.815648012	CYP2U1	Up
ENSOCUG00000014331	4.251676646	2.088031879	-	Up
ENSOCUG00000009989	2.741897211	1.455174488	AZIN2	Up
ENSOCUG00000026210	2.548355725	1.349566678	ORAI3	Up
ENSOCUG00000001436	3.327289027	1.734347192	MAOB	Up
ENSOCUG00000010739	10.52007867	3.395073588	SSPN	Up
ENSOCUG00000037591	2.505738347	1.325235774	-	Up
ENSOCUG00000009677	2.482617789	1.311862169	MYADM	Up
ENSOCUG00000012213	3.432617533	1.779309119	DYNC1I1	Up
ENSOCUG00000026428	2.723186309	1.445295689	GHDC	Up
ENSOCUG00000006320	2.512072007	1.328877819	SMARCA2	Up
ENSOCUG00000005548	4.811739678	2.266558593	AMIGO1	Up
ENSOCUG00000006727	3.95821828	1.984851174	NRSN2	Up
ENSOCUG00000014527	2.705714125	1.436009418	CTSC	Up
ENSOCUG00000021637	2.546214409	1.34835391	-	Up
ENSOCUG00000024025	2.912319805	1.542168788	CSKMT	Up
ENSOCUG00000015269	2.932314413	1.552039803	ADAP2	Up
ENSOCUG00000016289	4.160399878	2.0567222	HPGD	Up
ENSOCUG00000010809	2.817113322	1.494217599	CTC1	Up
ENSOCUG00000017793	4.643241525	2.215132327	-	Up
ENSOCUG00000008718	2.645278438	1.403419586	TMEM231	Up
ENSOCUG00000021984	10.93391731	3.450738465	C1QL4	Up
ENSOCUG00000023782	2.561747347	1.357128196	-	Up
ENSOCUG00000006073	2.519182702	1.332955756	-	Up

ENSOCUG00000010930	3.321494738	1.731832629	-	Up
ENSOCUG00000015952	Inf	Inf	TIMP4	Up
ENSOCUG00000035124	4.360716628	2.124565243	-	Up
ENSOCUG00000001723	4.311128261	2.108065485	ZFHX4	Up
ENSOCUG00000016171	2.871510002	1.521809587	CDH23	Up
ENSOCUG00000005109	3.869074707	1.951988586	-	Up
ENSOCUG00000023958	3.522401085	1.816559194	IL1B	Up
ENSOCUG00000007710	17.62016022	4.139155138	SCTR	Up
ENSOCUG00000013015	2.604487327	1.380999417	IFT122	Up
ENSOCUG00000011771	2.59597729	1.376277763	DENND4B	Up
ENSOCUG00000011919	3.855740255	1.947007867	SEMA3A	Up
ENSOCUG00000004294	18.0605791	4.174772248	NKX3-1	Up
ENSOCUG00000008707	3.683936664	1.881248258	RTL5	Up
ENSOCUG00000008776	Inf	Inf	FBXO43	Up
ENSOCUG00000032921	3.160571882	1.660185627	-	Up
ENSOCUG00000005696	2.900409336	1.536256523	-	Up
ENSOCUG00000011092	3.183462881	1.670596943	FLT4	Up
ENSOCUG00000002289	2.496304202	1.319793753	-	Up
ENSOCUG00000014904	2.44753075	1.291326985	EPAS1	Up
ENSOCUG00000004841	3.560487601	1.832074829	DKK3	Up
ENSOCUG00000004426	2.834405702	1.503046273	SOGA1	Up
ENSOCUG00000027124	2.492005822	1.317307439	RABAC1	Up
ENSOCUG00000005063	2.452876019	1.294474314	MXD4	Up
ENSOCUG00000003993	6.856451142	2.777462039	RFTN2	Up
ENSOCUG00000001937	2.43891209	1.286237758	ARHGEF2	Up
ENSOCUG00000029037	3.57360295	1.837379351	-	Up
ENSOCUG00000022849	2.554599689	1.353097236	COPG2	Up
ENSOCUG00000006352	3.10834266	1.636145554	FOXRED2	Up
ENSOCUG00000012203	4.024984631	2.008983274	SGSM1	Up
ENSOCUG00000000256	3.260382998	1.705041448	PTPDC1	Up
ENSOCUG00000006955	2.580606826	1.367710353	DNAJC18	Up
ENSOCUG00000004155	8.597215632	3.103869492	ACOX2	Up
ENSOCUG00000036162	3.836058849	1.939624853	-	Up
ENSOCUG00000006599	2.545259114	1.347812534	TAF10	Up
ENSOCUG00000012178	2.602201482	1.37973267	SIMC1	Up
ENSOCUG00000006299	2.697207302	1.431466409	ARVCF	Up
ENSOCUG00000006022	2.757857458	1.463547892	MINDY4	Up
ENSOCUG00000026025	4.227303184	2.079737587	CCDC65	Up
ENSOCUG00000008200	4.470993738	2.160595525	ALOX5AP	Up
ENSOCUG00000025564	3.049157247	1.608410553	PROCA1	Up
ENSOCUG00000003773	3.661323114	1.872365098	DTNA	Up
ENSOCUG00000007393	2.571386684	1.362546579	-	Up
ENSOCUG00000012485	2.503625684	1.324018882	-	Up
ENSOCUG00000012046	2.638858245	1.399913854	SNX24	Up

ENSOCUG00000007084	3.041200277	1.604640828	CHRD	Up
ENSOCUG00000002450	Inf	Inf	GRM4	Up
ENSOCUG00000009911	2.798027669	1.484410229	SLC9A9	Up
ENSOCUG00000014435	3.333844827	1.737186956	CFAP126	Up
ENSOCUG00000002215	15.40600046	3.945420469	BEX5	Up
ENSOCUG00000022794	2.782950061	1.47661502	F2R	Up
ENSOCUG00000001467	2.433698831	1.283150646	DLC1	Up
ENSOCUG00000021680	2.694444516	1.429987879	NUDT16	Up
ENSOCUG00000024222	3.263747214	1.706529321	-	Up
ENSOCUG00000035998	28.89365864	4.852680991	-	Up
ENSOCUG00000023508	2.505329961	1.325000624	-	Up
ENSOCUG00000016145	3.935305255	1.976475547	IL17B	Up
ENSOCUG00000008253	3.731580694	1.899786884	TFPI	Up
ENSOCUG00000013815	2.656537205	1.409546918	SEMA6A	Up
ENSOCUG00000022539	12.65305352	3.661413683	-	Up
ENSOCUG00000003485	3.177024069	1.66767602	-	Up
ENSOCUG00000010895	2.452310648	1.294141745	SVIL	Up
ENSOCUG00000005824	2.443257812	1.288806105	GLB1	Up
ENSOCUG00000001578	2.437927658	1.285655317	CUL7	Up
ENSOCUG00000002999	15.65691831	3.968728375	-	Up
ENSOCUG00000021938	3.706823942	1.890183595	-	Up
ENSOCUG00000003454	17.25010227	4.10853301	PRDM6	Up
ENSOCUG00000005284	2.384496817	1.253684857	IGSF3	Up
ENSOCUG00000003561	2.769072216	1.469402679	ZNF2	Up
ENSOCUG00000004970	2.493769361	1.318328042	ITPKB	Up
ENSOCUG00000012325	2.43040928	1.281199283	CDK5RAP3	Up
ENSOCUG00000017663	2.87731696	1.524724154	CLIP2	Up
ENSOCUG00000005534	2.712782233	1.439773241	-	Up
ENSOCUG00000029021	2.94937306	1.560408317	-	Up
ENSOCUG00000021444	3.714290216	1.893086545	-	Up
ENSOCUG00000008913	3.022392637	1.595691092	RPS6KA5	Up
ENSOCUG00000002809	2.606888057	1.382328633	PTPRG	Up
ENSOCUG00000014023	2.483943668	1.312632456	CDCA7L	Up
ENSOCUG00000000933	2.615858269	1.387284376	TMEM42	Up
ENSOCUG00000013992	2.445637104	1.290210345	TSPAN15	Up
ENSOCUG00000038441	3.037834825	1.603043429	-	Up
ENSOCUG00000001810	2.428953951	1.280335139	TPST2	Up
ENSOCUG00000017494	4.078549371	2.028056116	-	Up
ENSOCUG00000017822	3.476062215	1.797453904	-	Up
ENSOCUG00000013916	9.79909644	3.292648727	PRKCB	Up
ENSOCUG00000008832	4.235547099	2.082548332	CCDC113	Up
ENSOCUG00000023520	5.031192423	2.330900368	FLRT1	Up
ENSOCUG00000006969	6.566046695	2.71502501	DCN	Up
ENSOCUG00000023350	3.109731088	1.63678983	-	Up

ENSOCUG00000012115	2.464707026	1.301416167	AXIN2	Up
ENSOCUG00000015415	4.028489113	2.010238857	TDRP	Up
ENSOCUG00000026898	3.777847635	1.917564517	AKR1C26	Up
ENSOCUG00000002790	2.405143435	1.266122935	PYGB	Up
ENSOCUG00000027244	3.46922828	1.794614775	-	Up
ENSOCUG00000037754	2.664373002	1.413796069	HSD3B7	Up
ENSOCUG00000023579	3.10954492	1.636703458	-	Up
ENSOCUG00000009838	3.421357799	1.774568986	FIGN	Up
ENSOCUG00000011370	3.263432244	1.706390086	-	Up
ENSOCUG00000030939	2.629539937	1.394810408	-	Up
ENSOCUG00000008301	2.477994043	1.309172719	B4GALT4	Up
ENSOCUG00000003276	27.42216289	4.777270461	PAX1	Up
ENSOCUG00000039132	7.146272709	2.83719097	-	Up
ENSOCUG00000002701	8.922033311	3.157372534	CHADL	Up
ENSOCUG00000008909	8.549573309	3.09585242	-	Up
ENSOCUG00000005876	2.663616858	1.413386576	-	Up
ENSOCUG00000027932	2.432603279	1.282501058	STX10	Up
ENSOCUG00000011925	2.483378638	1.312304244	OSBPL10	Up
ENSOCUG00000012132	2.371502599	1.245801449	CHST2	Up
ENSOCUG00000015949	14.99603164	3.90650887	CRB1	Up
ENSOCUG00000004592	2.418031112	1.273832807	XYLT2	Up
ENSOCUG00000000659	4.05275694	2.018903654	NGF	Up
ENSOCUG00000004863	4.489137749	2.166438366	IQSEC3	Up
ENSOCUG00000000978	3.821633783	1.934189535	LOXL1	Up
ENSOCUG00000002522	4.253706336	2.088720437	PTGFR	Up
ENSOCUG00000023871	4.651657735	2.217744948	-	Up
ENSOCUG00000023973	3.831119636	1.937766078	-	Up
ENSOCUG00000028133	4.21675584	2.076133488	-	Up
ENSOCUG00000033345	3.65556002	1.870092439	-	Up
ENSOCUG00000030728	8.639483414	3.110945051	-	Up
ENSOCUG00000007013	2.702354678	1.434217038	SLC16A13	Up
ENSOCUG00000000487	2.641232519	1.401211314	OGFOD2	Up
ENSOCUG00000001407	6.917027696	2.790152233	MAGI2	Up
ENSOCUG00000033876	2.871225176	1.521666478	-	Up
ENSOCUG00000001047	4.069117492	2.024715938	SMARCA1	Up
ENSOCUG00000010937	2.525568177	1.336607987	SOCS2	Up
ENSOCUG00000009149	2.451575649	1.29370928	ECHDC3	Up
ENSOCUG00000022486	2.393197196	1.258939278	-	Up
ENSOCUG00000012140	Inf	Inf	-	Up
ENSOCUG00000000091	4.227555062	2.079823545	SERPINF2	Up
ENSOCUG00000006950	2.981606425	1.576089833	CECR2	Up
ENSOCUG00000015625	2.398389044	1.262065698	CUX1	Up
ENSOCUG00000016477	4.108415871	2.038582225	SELP	Up
ENSOCUG00000039248	3.283736718	1.71533846	ARMCX4	Up

ENSOCUG00000013088	2.387052383	1.255230226	-	Up
ENSOCUG00000007433	3.035544344	1.601955249	CHRNB1	Up
ENSOCUG00000023802	3.063724777	1.615286702	-	Up
ENSOCUG00000005211	4.968700012	2.312868441	PDGFRL	Up
ENSOCUG00000025713	2.460958061	1.299220072	ASGR1	Up
ENSOCUG00000023299	4.012132665	2.004369311	-	Up
ENSOCUG00000028148	2.432488747	1.282433131	PACS2	Up
ENSOCUG00000016560	3.318801493	1.73066234	ACKR4	Up
ENSOCUG00000010097	3.419853196	1.773934396	-	Up
ENSOCUG00000037528	5.300174844	2.406039952	-	Up
ENSOCUG00000024110	2.408754978	1.268287648	PALD1	Up
ENSOCUG00000006691	2.421641781	1.275985472	GSDME	Up
ENSOCUG00000031273	3.473386705	1.79634304	GPSM3	Up
ENSOCUG00000003641	3.538672362	1.823208192	LHX8	Up
ENSOCUG00000000655	2.60556186	1.381594507	HHAT	Up
ENSOCUG00000003108	2.993213594	1.581695232	TMEM44	Up
ENSOCUG00000008226	7.789808892	2.961587935	FBN2	Up
ENSOCUG00000001618	5.263008316	2.395887675	HGF	Up
ENSOCUG00000003387	2.844366427	1.508107333	KATNAL1	Up
ENSOCUG00000011018	2.667207826	1.415330242	-	Up
ENSOCUG00000017338	4.468570935	2.159813526	EDIL3	Up
ENSOCUG00000035778	3.439348243	1.7821352	-	Up
ENSOCUG00000003473	2.979938127	1.575282376	VIPR2	Up
ENSOCUG00000009332	6.625900944	2.728116636	MCOLN3	Up
ENSOCUG00000031663	4.216548257	2.076062466	-	Up
ENSOCUG00000024509	Inf	Inf	-	Up
ENSOCUG00000023789	2.41617008	1.272722013	-	Up
ENSOCUG00000024395	3.977952267	1.992025965	-	Up
ENSOCUG00000014861	2.394510335	1.259730662	MAPRE3	Up
ENSOCUG00000025335	4.937794485	2.303866792	SYNPO2L	Up
ENSOCUG00000022625	4.073508999	2.026272095	BANF2	Up
ENSOCUG00000024871	2.575125487	1.364642737	-	Up
ENSOCUG00000021270	2.356527432	1.236662476	PARP3	Up
ENSOCUG00000003059	2.466065734	1.302211256	CTBS	Up
ENSOCUG00000004150	2.35043135	1.232925544	FGFR1	Up
ENSOCUG00000015436	2.856265425	1.514130051	ADAM22	Up
ENSOCUG00000006957	2.7067961	1.436586215	IL1R1	Up
ENSOCUG00000005579	2.721590378	1.444449946	-	Up
ENSOCUG00000002897	2.38599676	1.254592084	CUL9	Up
ENSOCUG00000000754	2.560419304	1.356380091	CYP39A1	Up
ENSOCUG00000014241	3.08591694	1.625699231	RWDD3	Up
ENSOCUG00000007960	2.950475848	1.560947649	PLOD1	Up
ENSOCUG00000000608	2.316004789	1.211638236	PTGFRN	Up
ENSOCUG00000014231	3.529232991	1.819354676	TPBGL	Up

ENSOCUG00000012380	2.465467939	1.301861492	NPEPL1	Up
ENSOCUG00000015732	2.420534126	1.275325434	GASK1B	Up
ENSOCUG00000025543	4.6785464	2.226060362	-	Up
ENSOCUG00000002791	2.82487943	1.498189292	-	Up
ENSOCUG00000025317	20.55709615	4.361564582	PTP4A3	Up
ENSOCUG00000007048	2.404502898	1.265738665	KSR1	Up
ENSOCUG00000008704	2.525931522	1.336815528	SPATA6	Up
ENSOCUG00000012038	2.429114152	1.280430288	SLCO3A1	Up
ENSOCUG00000001613	2.459240574	1.298212873	AMER1	Up
ENSOCUG00000002530	4.084065557	2.030006024	PTGS1	Up
ENSOCUG00000014709	8.451524066	3.079211527	SLC7A4	Up
ENSOCUG00000000049	5.815584993	2.539924321	MASP1	Up
ENSOCUG00000027880	2.919771396	1.545855417	ZEB2	Up
ENSOCUG00000013643	2.276252678	1.186660714	PDLIM1	Up
ENSOCUG00000000318	2.39100544	1.257617412	-	Up
ENSOCUG00000012339	15.85114255	3.986514928	PEG3	Up
ENSOCUG00000012400	2.587331507	1.371464914	PTH1R	Up
ENSOCUG00000026191	4.478700634	2.163080236	-	Up
ENSOCUG00000016314	3.516169104	1.814004456	TMEM119	Up
ENSOCUG00000001923	3.920845646	1.971164848	CXCR4	Up
ENSOCUG00000039606	9.120440485	3.189103503	-	Up
ENSOCUG00000015875	2.737855092	1.45304609	RFTN1	Up
ENSOCUG00000006999	3.94291197	1.979261501	C2	Up
ENSOCUG00000029051	2.524140648	1.335792301	-	Up
ENSOCUG00000013134	2.932116486	1.55194242	ST8SIA1	Up
ENSOCUG00000031603	2.433198693	1.282854134	-	Up
ENSOCUG00000022244	2.651618345	1.406873139	TRPT1	Up
ENSOCUG00000011196	3.07814481	1.622061104	LAG3	Up
ENSOCUG00000005689	2.462560316	1.300159061	-	Up
ENSOCUG00000009457	2.486332892	1.31401947	SCRN2	Up
ENSOCUG00000003038	2.573175325	1.36354976	CRYZ	Up
ENSOCUG00000016172	2.347697548	1.231246559	STXBP1	Up
ENSOCUG00000029190	Inf	Inf	PLAC8	Up
ENSOCUG00000007997	2.483393972	1.312313152	-	Up
ENSOCUG00000030250	2.384340209	1.253590101	-	Up
ENSOCUG00000009580	3.322908321	1.73244649	WNT10B	Up
ENSOCUG00000001822	6.376950568	2.672866698	KIF17	Up
ENSOCUG00000027943	4.3408056	2.117962814	C19orf38	Up
ENSOCUG00000012592	3.04494435	1.606415861	ASB5	Up
ENSOCUG00000011960	3.151318779	1.655955701	ECE1	Up
ENSOCUG00000014271	2.317693475	1.212689776	HADH	Up
ENSOCUG00000003448	2.407535139	1.267556855	DENND6B	Up
ENSOCUG00000006212	2.391494952	1.257912746	POLR3GL	Up
ENSOCUG00000005032	4.487845687	2.16602307	-	Up

ENSOCUG00000008330	2.526369841	1.337065854	-	Up
ENSOCUG00000004686	2.469748545	1.304364163	TENM4	Up
ENSOCUG00000015054	15.18159171	3.924251153	UNC5C	Up
ENSOCUG00000001830	7.470828902	2.901268322	DOK6	Up
ENSOCUG00000016783	3.346824773	1.742793019	DTWD1	Up
ENSOCUG00000023063	Inf	Inf	-	Up
ENSOCUG00000010039	2.299438231	1.201281444	FHOD1	Up
ENSOCUG00000027803	7.139051279	2.835732365	SH3GL3	Up
ENSOCUG00000026694	2.888999854	1.530570131	-	Up
ENSOCUG00000009011	2.472535033	1.305990962	-	Up
ENSOCUG00000017019	3.425836003	1.77645609	CDH11	Up
ENSOCUG00000009563	2.377653723	1.249538619	TMEM263	Up
ENSOCUG00000016056	2.311417828	1.208778075	SGSM2	Up
ENSOCUG00000011229	2.280873756	1.189586597	RNF114	Up
ENSOCUG00000016271	2.319091693	1.213559863	NT5DC3	Up
ENSOCUG00000005937	2.615736068	1.387216978	SCD5	Up
ENSOCUG00000005922	2.368196681	1.243788903	ASRGL1	Up
ENSOCUG00000002368	2.302803421	1.203391261	GGCX	Up
ENSOCUG00000022309	2.334979265	1.223409739	-	Up
ENSOCUG00000017068	2.25303784	1.171871544	ZNF608	Up
ENSOCUG00000009404	5.817680062	2.540443959	KIF19	Up
ENSOCUG00000007786	2.799750896	1.485298471	-	Up
ENSOCUG00000005997	8.771254263	3.132783158	MEIOB	Up
ENSOCUG00000011468	14.17137853	3.824908199	RDH8	Up
ENSOCUG00000003384	6.916485052	2.790039048	PRKAG3	Up
ENSOCUG00000010349	2.309767926	1.207747904	HIBADH	Up
ENSOCUG00000014924	25.57583274	4.676709309	TMEM130	Up
ENSOCUG00000006248	2.947142677	1.559316906	-	Up
ENSOCUG00000002496	3.097704573	1.631199562	-	Up
ENSOCUG00000003498	2.605727915	1.381686448	SPEF1	Up
ENSOCUG00000021251	3.331837851	1.736318191	USP49	Up
ENSOCUG00000009914	2.552180585	1.351730414	ARHGEF40	Up
ENSOCUG00000000237	2.486612009	1.314181419	TRIM45	Up
ENSOCUG00000010326	2.289368839	1.194949913	RTL10	Up
ENSOCUG00000021272	2.436872503	1.285030771	TANGO2	Up
ENSOCUG00000001851	2.274686352	1.185667631	NCKAP5L	Up
ENSOCUG00000009570	3.204806346	1.680237183	TTC28	Up
ENSOCUG00000014513	2.589911898	1.372903022	IL27RA	Up
ENSOCUG00000005204	2.267245551	1.180940649	RGS4	Up
ENSOCUG00000001661	2.219461294	1.150209549	PDIA4	Up
ENSOCUG00000023116	2.325260688	1.217392468	AGO1	Up
ENSOCUG00000007943	2.274268761	1.185402754	-	Up
ENSOCUG00000016944	5.423842914	2.439315396	BVES	Up
ENSOCUG00000002336	2.305944203	1.205357605	BST1	Up

ENSOCUG00000004773	5.593137237	2.48365773	MACROH2A2	Up
ENSOCUG00000004959	9.432561336	3.237649576	HHIP	Up
ENSOCUG00000027484	4.166602323	2.05887141	PPP1R3E	Up
ENSOCUG00000023055	2.402572616	1.264580036	TXNDC5	Up
ENSOCUG00000035995	2.512940887	1.329376735	-	Up
ENSOCUG00000007547	3.766313184	1.913152971	GPR135	Up
ENSOCUG00000024457	3.120246238	1.641659886	CLIP3	Up
ENSOCUG00000002157	2.260299842	1.176514167	NEO1	Up
ENSOCUG00000004914	3.17632567	1.66735884	RAB36	Up
ENSOCUG00000012007	4.246932712	2.086421251	PRR5L	Up
ENSOCUG00000003341	2.393147191	1.258909133	-	Up
ENSOCUG00000017607	2.496536299	1.319927883	NMT2	Up
ENSOCUG00000000255	3.124852317	1.643788008	ARHGEF6	Up
ENSOCUG00000016703	2.31433324	1.210596612	TRERF1	Up
ENSOCUG00000011841	2.257046412	1.174436085	LIG1	Up
ENSOCUG00000021711	25.83915084	4.691486754	PPP1R1B	Up
ENSOCUG00000017922	2.297957308	1.200351995	ABCD1	Up
ENSOCUG00000005668	2.307637605	1.206416679	TRAF1	Up
ENSOCUG00000006025	8.443317131	3.077809903	-	Up
ENSOCUG00000010835	2.437569555	1.285443386	NOL4L	Up
ENSOCUG00000017585	3.816875215	1.932392021	OTULINL	Up
ENSOCUG00000016548	3.169372278	1.66419713	ROR2	Up
ENSOCUG00000005788	3.033883902	1.601165879	SLC22A7	Up
ENSOCUG00000012304	13.05763556	3.706821776	-	Up
ENSOCUG00000021079	2.259384031	1.175929509	-	Up
ENSOCUG00000016179	4.695813863	2.231375223	THEMIS2	Up
ENSOCUG00000011801	6.862216446	2.778674632	RNF165	Up
ENSOCUG00000023464	2.367186254	1.243173224	SOX13	Up
ENSOCUG00000023203	9.803801416	3.293341261	ADGRA1	Up
ENSOCUG00000011690	2.320323666	1.214326064	SLC25A28	Up
ENSOCUG00000003525	2.365830938	1.242346982	-	Up
ENSOCUG00000016426	3.248516142	1.699780874	AGER	Up
ENSOCUG00000001046	4.059548206	2.021319176	IBSP	Up
ENSOCUG00000003984	6.075570797	2.603019956	SLC8A3	Up
ENSOCUG00000007830	2.745520594	1.457079733	IL31RA	Up
ENSOCUG00000012701	9.421935092	3.236023393	TLR8	Up
ENSOCUG00000011775	3.895602548	1.961846493	RIMS2	Up
ENSOCUG00000035695	Inf	Inf	-	Up
ENSOCUG00000009072	2.748529175	1.458659793	-	Up
ENSOCUG00000005519	3.142808909	1.652054555	ARMC12	Up
ENSOCUG00000013958	2.296175304	1.19923279	MMAB	Up
ENSOCUG00000013360	2.432308276	1.28232609	ZNF346	Up
ENSOCUG00000015065	2.579553014	1.367121096	NFKBIL1	Up
ENSOCUG00000012747	2.285840611	1.192724809	PRKAR2B	Up

ENSOCUG00000012904	2.30896441	1.207245936	LAYN	Up
ENSOCUG00000015267	2.281224199	1.189808242	GPX8	Up
ENSOCUG00000017370	2.229845947	1.156944042	FRMD4A	Up
ENSOCUG00000005790	4.998350646	2.321452113	FAM81A	Up
ENSOCUG00000002587	3.461322812	1.791323497	TSHZ2	Up
ENSOCUG00000003860	2.290692675	1.195783916	PEX6	Up
ENSOCUG00000013461	2.278343125	1.187985037	CRYL1	Up
ENSOCUG00000000094	3.503272451	1.808703191	-	Up
ENSOCUG00000026351	2.696973965	1.431341595	-	Up
ENSOCUG00000026503	2.666762549	1.415089372	-	Up
ENSOCUG00000014795	2.342224482	1.227879353	IGF1R	Up
ENSOCUG00000023997	2.511580889	1.32859574	-	Up
ENSOCUG00000005584	2.38320176	1.252901094	-	Up
ENSOCUG00000001591	2.260548968	1.17667317	ZNF516	Up
ENSOCUG00000004630	2.270505236	1.183013364	AKNA	Up
ENSOCUG00000013137	2.768440094	1.469073304	CETP	Up
ENSOCUG00000003182	2.307990786	1.206637465	TMEM140	Up
ENSOCUG00000002090	4.562595298	2.189854692	RASSF2	Up
ENSOCUG00000010551	3.504858399	1.809356159	SLC7A3	Up
ENSOCUG00000005602	2.942016207	1.556805194	P3H1	Up
ENSOCUG00000014771	2.53230054	1.340448638	TBKBP1	Up
ENSOCUG00000012912	2.37527292	1.248093289	-	Up
ENSOCUG00000011681	2.19383254	1.133453406	GNAI2	Up
ENSOCUG00000010238	2.695527742	1.430567758	PIEZO2	Up
ENSOCUG00000006439	2.231920023	1.158285331	ZHX3	Up
ENSOCUG00000012160	2.195686048	1.134671784	PRRC1	Up
ENSOCUG00000026676	2.759712349	1.464517899	-	Up
ENSOCUG00000010827	2.265023529	1.179526037	BOC	Up
ENSOCUG00000016942	3.163843153	1.66167808	ALDH1A2	Up
ENSOCUG00000022152	2.223883425	1.153081164	RFC2	Up
ENSOCUG00000005275	2.333424987	1.222449089	NPRL2	Up
ENSOCUG00000004145	2.300382454	1.201873739	ATP7B	Up
ENSOCUG00000017751	3.513271248	1.812814966	-	Up
ENSOCUG00000012388	7.928390131	2.987027955	EMILIN3	Up
ENSOCUG00000007313	2.222543092	1.152211391	CREB3L2	Up
ENSOCUG00000003251	10.6976314	3.419219495	ZNF426	Up
ENSOCUG00000001555	3.480939645	1.7994768	ACE	Up
ENSOCUG00000000025	2.296406402	1.199377983	SLCO2A1	Up
ENSOCUG00000029231	2.203092482	1.139530058	-	Up
ENSOCUG00000034046	2.315873395	1.211556386	-	Up
ENSOCUG00000004298	Inf	Inf	NT5C1A	Up
ENSOCUG00000022623	2.234667009	1.160059869	CISD3	Up
ENSOCUG00000008544	2.163802982	1.113569145	ADAR	Up
ENSOCUG00000011487	5.111239401	2.353673166	DKK1	Up

ENSOCUG00000008956	3.445246598	1.784607249	PCTP	Up
ENSOCUG00000013782	2.23106302	1.157731266	PHKA2	Up
ENSOCUG00000017830	2.214631894	1.147066921	NPRL3	Up
ENSOCUG00000000919	2.196157431	1.134981477	-	Up
ENSOCUG00000009507	Inf	Inf	ADAMTS16	Up
ENSOCUG00000007437	2.228846377	1.156297182	ZBTB4	Up
ENSOCUG00000013230	2.296789	1.199618326	MTCL1	Up
ENSOCUG00000001335	3.935348917	1.976491554	-	Up
ENSOCUG00000007245	2.211817573	1.145232399	SIRT5	Up
ENSOCUG00000002541	2.198935106	1.136805028	BNIP3	Up
ENSOCUG00000010384	7.521975052	2.911111521	IL16	Up
ENSOCUG00000030576	24.21105917	4.597594289	-	Up
ENSOCUG00000011739	2.408615188	1.26820392	RUNX2	Up
ENSOCUG00000006943	2.148044734	1.103024039	UNC5B	Up
ENSOCUG00000004664	3.963362677	1.986724991	M1AP	Up
ENSOCUG00000007964	2.498672367	1.321161743	TBC1D4	Up
ENSOCUG00000009809	4.948359728	2.306950383	-	Up
ENSOCUG00000006404	5.195822908	2.377352259	SLC6A7	Up
ENSOCUG00000034585	5.378072348	2.427089163	MTRNR2L10	Up
ENSOCUG00000023626	4.270618115	2.094444896	-	Up
ENSOCUG00000035337	2.887358476	1.529750235	-	Up
ENSOCUG00000024841	14.1922296	3.827029349	LRRC74B	Up
ENSOCUG00000006958	3.656210982	1.870349323	STING1	Up
ENSOCUG00000017890	3.567997545	1.835114623	MDGA1	Up
ENSOCUG00000012514	3.01093352	1.590210854	XRRA1	Up
ENSOCUG00000001515	Inf	Inf	CCL19	Up
ENSOCUG00000002876	2.762136056	1.465784385	TBCEL-TECT A	Up
ENSOCUG00000007719	3.820752496	1.933856804	PDE3B	Up
ENSOCUG00000014994	2.235255393	1.160439679	UNK	Up
ENSOCUG00000010911	2.696768715	1.431231796	PELI3	Up
ENSOCUG00000025997	2.516900474	1.331648169	-	Up
ENSOCUG00000014833	5.773913353	2.529549458	PLA2R1	Up
ENSOCUG00000009767	2.186079091	1.128345598	DTNBP1	Up
ENSOCUG00000002218	2.310815324	1.208401967	-	Up
ENSOCUG00000014660	8.643036503	3.111538254	CNKSR2	Up
ENSOCUG00000014689	96.80303866	6.596980429	IGF1	Up
ENSOCUG00000012565	2.293950113	1.197834017	FUT11	Up
ENSOCUG00000010032	2.285761119	1.192674638	THBS3	Up
ENSOCUG00000007741	2.168094297	1.116427505	CRTAP	Up
ENSOCUG00000010260	2.229990129	1.157037324	RASSF4	Up
ENSOCUG00000026607	2.775682875	1.472842748	-	Up
ENSOCUG00000035406	2.800163808	1.485511226	PRAF2	Up
ENSOCUG00000005097	2.189285618	1.130460184	BRD3	Up

ENSOCUG00000003921	2.496306348	1.319794993	GLIPR1	Up
ENSOCUG00000010271	20.48560162	4.356538357	STX1B	Up
ENSOCUG00000029426	2.160314492	1.111241351	-	Up
ENSOCUG00000023342	2.174586295	1.120740961	RTN2	Up
ENSOCUG00000015314	Inf	Inf	-	Up
ENSOCUG00000002183	2.176448499	1.121975882	PML	Up
ENSOCUG00000023355	9.060310899	3.179560556	MYO7A	Up
ENSOCUG00000005036	2.390336057	1.25721346	-	Up
ENSOCUG00000011473	3.591081899	1.844418556	PRKG1	Up
ENSOCUG00000012919	3.089537534	1.627390901	DIXDC1	Up
ENSOCUG00000005128	3.30477187	1.724550685	PPP1R9A	Up
ENSOCUG00000039678	3.607785356	1.851113508	-	Up
ENSOCUG00000008132	2.153069911	1.106395165	-	Up
ENSOCUG00000004990	2.371819793	1.2459944	APEH	Up
ENSOCUG00000001235	3.349203259	1.743817934	ADAM19	Up
ENSOCUG00000007017	3.250362327	1.700600548	SLC16A11	Up
ENSOCUG00000007880	2.21711616	1.148684358	NIPSNAP3A	Up
ENSOCUG00000005158	2.530169118	1.339233819	-	Up
ENSOCUG00000025595	2.433994356	1.283325823	RHBDD3	Up
ENSOCUG00000010540	3.978882148	1.992363168	-	Up
ENSOCUG00000003157	2.226387116	1.154704465	DCBLD1	Up
ENSOCUG00000001030	3.843430964	1.942394755	GEM	Up
ENSOCUG00000006386	2.107558896	1.075572948	ATP10A	Up
ENSOCUG00000034394	2.233309259	1.159183043	-	Up
ENSOCUG00000002318	2.158998765	1.110362418	NUAK2	Up
ENSOCUG00000021497	2.244473582	1.166377116	-	Up
ENSOCUG00000011359	2.280309255	1.189229496	ACAD10	Up
ENSOCUG00000029716	2.135566816	1.094619036	ARHGEF17	Up
ENSOCUG00000026956	23.92206916	4.580270277	GDF7	Up
ENSOCUG00000010201	2.149950079	1.104303161	NEK9	Up
ENSOCUG00000010558	2.155867999	1.108268846	PCCB	Up
ENSOCUG00000001750	2.610341796	1.384238725	PRPF40B	Up
ENSOCUG00000013036	7.047378348	2.817086669	RHO	Up
ENSOCUG00000015051	4.036790502	2.013208717	PPP1R3C	Up
ENSOCUG00000001524	2.494229452	1.318594189	-	Up
ENSOCUG00000025219	3.128956037	1.645681388	OAF	Up
ENSOCUG00000005892	2.855003465	1.513492497	ARHGAP33	Up
ENSOCUG00000023066	2.155911942	1.108298252	STAT5A	Up
ENSOCUG00000010026	2.230190132	1.15716671	-	Up
ENSOCUG00000010023	2.168724207	1.1168466	ABHD14B	Up
ENSOCUG00000015336	8.317829492	3.056207111	DNAI1	Up
ENSOCUG00000024508	2.19309872	1.132970755	-	Up
ENSOCUG00000027921	2.390863873	1.25753199	-	Up
ENSOCUG00000015752	3.350527892	1.744388417	ACSS3	Up

ENSOCUG00000029321	2.212644394	1.145771607	MPV17	Up
ENSOCUG00000021633	2.64259236	1.401953895	LIMS2	Up
ENSOCUG00000014685	2.158477706	1.110014192	FBXW8	Up
ENSOCUG00000004947	2.424811074	1.277872346	SLC45A2	Up
ENSOCUG00000006639	3.085822224	1.62565495	ENOX1	Up
ENSOCUG00000027228	22.42157047	4.486815427	SMTNL1	Up
ENSOCUG00000011921	6.498565523	2.700121296	FAM216A	Up
ENSOCUG00000016799	2.196238147	1.1350345	CYB5R1	Up
ENSOCUG00000010654	2.303990142	1.204134544	ARHGAP24	Up
ENSOCUG00000027034	Inf	Inf	-	Up
ENSOCUG00000008062	2.18168224	1.125440989	-	Up
ENSOCUG00000016823	2.374936814	1.24788913	COBL	Up
ENSOCUG00000003017	2.586542703	1.37102501	ERICH3	Up
ENSOCUG00000021414	4.947840035	2.306798858	RIIAD1	Up
ENSOCUG00000013030	2.223026668	1.152525255	TMEM101	Up
ENSOCUG00000004080	2.279427683	1.188671639	RASL10B	Up
ENSOCUG00000007820	2.194229138	1.133714191	ERAP1	Up
ENSOCUG00000001704	Inf	Inf	LRRC30	Up
ENSOCUG00000015922	3.188033412	1.67266675	RNASEL	Up
ENSOCUG00000015702	2.351129362	1.23335392	EPOR	Up
ENSOCUG00000012643	2.139640372	1.097368331	OSMR	Up
ENSOCUG00000011389	2.625497233	1.392590675	-	Up
ENSOCUG00000006661	2.176469815	1.121990012	TRIM47	Up
ENSOCUG00000007709	2.250637863	1.17033394	IFT22	Up
ENSOCUG00000016068	4.400160682	2.137556208	SERPINE1	Up
ENSOCUG00000024268	2.162447676	1.112665225	IFT140	Up
ENSOCUG00000026152	2.547312809	1.348976133	DBN1	Up
ENSOCUG00000027852	2.185551208	1.127997181	IQSEC2	Up
ENSOCUG00000009804	2.147490297	1.102651613	STX2	Up
ENSOCUG00000010695	2.177877812	1.122923015	BABAM2	Up
ENSOCUG00000005220	2.158981917	1.11035116	POMGNT1	Up
ENSOCUG00000006186	4.40140259	2.137963339	CPXM1	Up
ENSOCUG00000013824	2.154306121	1.107223268	KIFAP3	Up
ENSOCUG00000006228	2.564059345	1.358429654	SUSD4	Up
ENSOCUG00000007453	3.590514381	1.844190541	SMAD9	Up
ENSOCUG00000002153	2.22219855	1.151987725	ATAT1	Up
ENSOCUG00000013453	3.360680576	1.748753425	HMCN1	Up
ENSOCUG00000038690	3.456919191	1.789486879	IRGC	Up
ENSOCUG00000010299	2.096032984	1.06766142	CSRP1	Up
ENSOCUG00000001655	40.2436399	5.330688893	-	Up
ENSOCUG00000010077	Inf	Inf	KCTD19	Up
ENSOCUG00000012663	2.65742565	1.410029328	MSTN	Up
ENSOCUG00000002413	4.311821551	2.108297472	HOXD4	Up
ENSOCUG00000005540	12.31109541	3.62188723	CAPN6	Up

ENSOCUG00000026756	2.159480343	1.110684184	ALDH16A1	Up
ENSOCUG00000025354	14.82440379	3.889902178	-	Up
ENSOCUG00000011626	3.909207535	1.966876178	CAMK1D	Up
ENSOCUG00000004792	2.27297553	1.184582153	TATDN3	Up
ENSOCUG00000026268	3.569995367	1.835922202	ADIPOQ	Up
ENSOCUG00000009811	2.417970177	1.27379645	ZNFX1	Up
ENSOCUG00000025758	2.15627706	1.108542562	-	Up
ENSOCUG00000033016	2.365980004	1.242437881	-	Up
ENSOCUG00000012937	2.421995895	1.27619642	PRKACB	Up
ENSOCUG00000000763	4.20967751	2.073709717	PLA2G7	Up
ENSOCUG00000025312	2.892925412	1.532529127	-	Up
ENSOCUG00000014466	2.165470717	1.114680663	-	Up
ENSOCUG00000021573	2.149214933	1.103809768	DNASE2	Up
ENSOCUG00000022248	9.580586929	3.260114041	RUFY4	Up
ENSOCUG00000037257	2.118453575	1.083011513	PAQR7	Up
ENSOCUG00000027575	3.7195292	1.895120024	KCNH2	Up
ENSOCUG00000004935	3.746024251	1.905360239	CYP24A1	Up
ENSOCUG00000014035	2.489823097	1.316043242	SH3BGRL	Up
ENSOCUG00000015613	2.105101932	1.073890092	TPST1	Up
ENSOCUG00000005189	2.095703232	1.067434434	-	Up
ENSOCUG00000011260	2.270142795	1.182783048	DNAJC27	Up
ENSOCUG00000015244	3.370381803	1.752912032	-	Up
ENSOCUG00000006656	3.72269201	1.896346262	DAAM2	Up
ENSOCUG00000014570	4.533132775	2.180508418	FRRS1L	Up
ENSOCUG00000003589	2.658579281	1.41065549	ENO2	Up
ENSOCUG00000009110	38.99877697	5.285356975	PPP1R17	Up
ENSOCUG00000035146	2.205133885	1.140866252	-	Up
ENSOCUG00000027604	2.135005268	1.09423963	MLLT6	Up
ENSOCUG00000001675	2.109827933	1.077125345	PHF21A	Up
ENSOCUG00000007140	2.079728629	1.056395292	ABL1	Up
ENSOCUG00000022021	4.313804403	2.108960763	IL22RA2	Up
ENSOCUG00000005930	3.989335393	1.996148419	ZFPM2	Up
ENSOCUG00000006952	3.009359398	1.589456413	SPATA24	Up
ENSOCUG00000027965	3.461338152	1.791329891	CYP46A1	Up
ENSOCUG00000023029	3.106252966	1.635175324	CLEC2L	Up
ENSOCUG00000013176	2.092209718	1.065027471	SMO	Up
ENSOCUG00000029390	2.610273011	1.384200707	PTGES3L	Up
ENSOCUG00000001724	3.257168234	1.703618238	PRRX2	Up
ENSOCUG00000011021	4.097010637	2.034571638	MYBPH	Up
ENSOCUG00000008561	3.795759549	1.924388605	-	Up
ENSOCUG00000011457	2.130395242	1.091121111	FZD7	Up
ENSOCUG00000017352	2.057309027	1.040758517	-	Up
ENSOCUG00000026356	Inf	Inf	-	Up
ENSOCUG00000013315	5.809442097	2.538399623	-	Up

ENSOCUG00000012391	2.074589423	1.052825845	PRPS1	Up
ENSOCUG00000007223	2.283319682	1.191132862	EIF2AK2	Up
ENSOCUG00000029223	2.817027678	1.494173739	ZNF699	Up
ENSOCUG00000001863	2.47680038	1.308477598	VAT1L	Up
ENSOCUG00000010919	9.436522285	3.23825527	PABPC5	Up
ENSOCUG00000003142	2.589451523	1.37264655	AR	Up
ENSOCUG00000033705	2.280187318	1.189152347	-	Up
ENSOCUG00000024985	2.177550635	1.122706267	RNF215	Up
ENSOCUG00000003425	2.101045825	1.071107628	GLG1	Up
ENSOCUG00000007731	2.190792856	1.131453081	PDZRN3	Up
ENSOCUG00000021137	2.785707609	1.478043839	SFRP5	Up
ENSOCUG00000009436	7.241021333	2.856193201	CNGA2	Up
ENSOCUG00000009400	Inf	Inf	DNAI2	Up
ENSOCUG00000037275	15.38564059	3.943512607	-	Up
ENSOCUG00000021275	2.210338092	1.14426706	ENDOG	Up
ENSOCUG00000024688	2.127027561	1.088838727	DNMT3A	Up
ENSOCUG00000011938	2.106959553	1.075162619	TCTN1	Up
ENSOCUG00000021918	2.118581574	1.08309868	-	Up
ENSOCUG00000022990	18.82572346	4.234633403	-	Up
ENSOCUG00000004825	3.177616693	1.667945107	DRD1	Up
ENSOCUG00000010891	2.157505044	1.109363932	-	Up
ENSOCUG00000010557	2.131108086	1.091603766	MANBA	Up
ENSOCUG00000014464	5.258476579	2.3946449	GAS2	Up
ENSOCUG00000002617	2.425670922	1.278383841	YDJC	Up
ENSOCUG00000006121	3.205986722	1.680768451	APLF	Up
ENSOCUG00000012991	2.02683145	1.01922612	PDIA6	Up
ENSOCUG00000006606	3.022684055	1.59583019	DCHS1	Up
ENSOCUG00000027538	7.547276729	2.915956173	-	Up
ENSOCUG00000029346	4.648625166	2.216804101	CALY	Up
ENSOCUG00000009029	2.610415204	1.384279295	ENKD1	Up
ENSOCUG00000038679	2.102316459	1.071979852	-	Up
ENSOCUG00000004582	4.546305488	2.184694629	DMRT3	Up
ENSOCUG00000013831	9.35298539	3.225426934	TMOD1	Up
ENSOCUG00000016547	4.551502124	2.186342754	CCDC89	Up
ENSOCUG00000003806	21.53930496	4.428899792	KY	Up
ENSOCUG00000033686	Inf	Inf	-	Up
ENSOCUG00000002134	2.24190838	1.164727321	EMP3	Up
ENSOCUG00000023638	4.926489242	2.300559907	KCNIP3	Up
ENSOCUG00000022137	2.224763856	1.153652211	PEMT	Up
ENSOCUG00000012674	2.807396166	1.489232664	CACNA1B	Up
ENSOCUG00000013870	2.585515254	1.370451817	MOK	Up
ENSOCUG00000026203	2.660661093	1.411784756	C1QL1	Up
ENSOCUG00000001454	3.529323827	1.819391808	NES	Up
ENSOCUG00000001727	2.171659604	1.118797986	SLC19A1	Up

ENSOCUG00000011611	3.935406777	1.976512765	SLC2A9	Up
ENSOCUG00000025234	2.075034166	1.053135091	-	Up
ENSOCUG00000032659	2.274792011	1.185734643	-	Up
ENSOCUG00000020990	5.951304974	2.57320605	-	Up
ENSOCUG00000008091	2.132680224	1.092667663	SLC2A8	Up
ENSOCUG00000000635	5.011428286	2.325221839	EYA4	Up
ENSOCUG00000021313	3.956684302	1.984291959	C11orf96	Up
ENSOCUG00000039281	2.363992679	1.241225567	-	Up
ENSOCUG00000011564	2.108827066	1.076440792	RFX5	Up
ENSOCUG00000009635	2.0854467	1.06035644	-	Up
ENSOCUG00000016283	2.998976361	1.58447015	RPL3L	Up
ENSOCUG00000012533	2.013172079	1.009470494	-	Up
ENSOCUG00000003431	5.377263156	2.426872077	BMP6	Up
ENSOCUG00000031548	2.486405812	1.314061781	-	Up
ENSOCUG00000031409	3.335032533	1.737700835	PCOLCE	Up
ENSOCUG00000004495	2.294499161	1.198179279	PYCARD	Up
ENSOCUG00000006333	2.127148572	1.088920802	-	Up
ENSOCUG00000038149	4.080993302	2.028920343	SMIM17	Up
ENSOCUG00000004124	2.607581119	1.382712134	TMEM117	Up
ENSOCUG00000023448	2.112349961	1.078848871	GSTM2	Up
ENSOCUG00000016759	2.125216193	1.087609611	CBR3	Up
ENSOCUG00000016324	2.39985693	1.262948401	CIT	Up
ENSOCUG00000003818	2.067821401	1.048111584	TRIM44	Up
ENSOCUG00000003112	5.15778722	2.366752257	STRC	Up
ENSOCUG00000014610	2.072687408	1.051502553	ABCC4	Up
ENSOCUG00000012137	2.100407644	1.070669351	-	Up
ENSOCUG00000005575	3.273913977	1.711016415	FLOT2	Up
ENSOCUG00000020977	2.178000808	1.123004489	-	Up
ENSOCUG00000021436	Inf	Inf	-	Up
ENSOCUG00000000171	4.081679096	2.029162761	GREM2	Up
ENSOCUG00000024365	2.11895106	1.083350267	ARF5	Up
ENSOCUG00000022823	2.021204111	1.015215019	ID3	Up
ENSOCUG00000015416	2.095700447	1.067432517	ZNF689	Up
ENSOCUG00000010677	5.816731756	2.540208774	INSRR	Up
ENSOCUG00000025258	2.063153161	1.044850926	RPL13A	Up
ENSOCUG00000027869	2.179705767	1.124133402	-	Up
ENSOCUG00000010173	2.528696587	1.338393941	PHLDB1	Up
ENSOCUG00000005870	2.286616763	1.19321459	ST3GAL3	Up
ENSOCUG00000016155	2.096608754	1.068057667	-	Up
ENSOCUG00000005369	2.004904725	1.00353368	CERS4	Up
ENSOCUG00000006893	7.588432396	2.923801887	MARCO	Up
ENSOCUG00000017432	21.57884462	4.431545717	SELL	Up
ENSOCUG00000004877	7.22351784	2.852701598	ZNF385B	Up
ENSOCUG00000006752	2.576615818	1.365477443	MEST	Up

ENSOCUG00000015706	2.065575794	1.046543999	-	Up
ENSOCUG00000011901	4.191710469	2.06753907	EN1	Up
ENSOCUG00000024399	2.380851552	1.25147767	NAB2	Up
ENSOCUG00000005078	2.053223217	1.037890479	STAT5B	Up
ENSOCUG00000021276	3.680812895	1.880024416	MRAP	Up
ENSOCUG00000012677	4.542523097	2.18349385	ENTPD1	Up
ENSOCUG00000023671	2.352538552	1.234218365	FAM131A	Up
ENSOCUG00000025664	2.087662209	1.061888298	C17orf49	Up
ENSOCUG00000025033	2.816942758	1.494130247	KIAA0895L	Up
ENSOCUG00000008578	3.586806175	1.842699787	DYNLRB2	Up
ENSOCUG00000012528	2.063459609	1.045065198	ST3GAL4	Up
ENSOCUG00000003892	3.124317124	1.643540897	-	Up
ENSOCUG00000024717	2.599952552	1.378485295	-	Up
ENSOCUG00000010933	21.35149717	4.41626533	POU3F3	Up
ENSOCUG00000036875	11.49715456	3.523204947	-	Up
ENSOCUG00000000247	2.038101093	1.027225614	CHPF2	Up
ENSOCUG00000022952	2.030682902	1.021964976	NUCB1	Up
ENSOCUG00000007587	Inf	Inf	-	Up
ENSOCUG00000017943	2.829792096	1.500696063	LCP1	Up
ENSOCUG00000027712	2.009536486	1.006862772	-	Up
ENSOCUG00000021400	2.341064024	1.22716439	-	Up
ENSOCUG00000004401	2.584064533	1.3696421	MPDZ	Up
ENSOCUG00000014216	2.054060824	1.038478903	CARMIL1	Up
ENSOCUG00000023105	2.410516614	1.269342373	RFLNB	Up
ENSOCUG00000004900	2.019279913	1.013840912	PALLD	Up
ENSOCUG00000011284	2.910658091	1.541345379	SLC7A2	Up
ENSOCUG00000021629	2.248624525	1.16904278	RASIP1	Up
ENSOCUG00000004554	4.077332941	2.027625767	-	Up
ENSOCUG00000008134	2.679656514	1.422048084	CHN1	Up
ENSOCUG00000002504	2.214152202	1.146754397	WWOX	Up
ENSOCUG00000023161	2.577252289	1.36583377	RYR1	Up
ENSOCUG00000023490	2.034414167	1.024613414	PRICKLE1	Up
ENSOCUG00000024100	7.723062338	2.949173016	PPM1N	Up
ENSOCUG00000004764	2.692088479	1.428725827	LGI2	Up
ENSOCUG00000011625	2.039123853	1.027949405	TTC12	Up
ENSOCUG00000007603	2.511424246	1.328505758	CHST15	Up
ENSOCUG00000006962	2.457631854	1.29726882	ALDH1L2	Up
ENSOCUG00000035389	2.008070515	1.005809932	-	Up
ENSOCUG00000002233	2.30037612	1.201869767	HSH2D	Up
ENSOCUG00000005351	2.087929154	1.06207276	DENND1A	Up
ENSOCUG00000017594	12.09111326	3.595875178	-	Up
ENSOCUG00000015888	2.05032174	1.035850318	FBXO31	Up
ENSOCUG00000010436	2.43613316	1.284592993	LEF1	Up
ENSOCUG00000015559	3.521228256	1.81607875	DRC1	Up

ENSOCUG00000008023	5.333400244	2.415055599	ABCC9	Up
ENSOCUG00000033568	9.102519975	3.186266002	SMIM1	Up
ENSOCUG00000006286	2.032271173	1.023092919	-	Up
ENSOCUG00000000280	20.57541062	4.362849317	TAFA3	Up
ENSOCUG00000030304	6.778316856	2.760927078	LINC00672	Up
ENSOCUG00000036758	2.47675092	1.308448788	OTUB2	Up
ENSOCUG00000015384	2.125590568	1.087863731	OXSM	Up
ENSOCUG00000005331	2.035413187	1.02532169	TMEM94	Up
ENSOCUG00000023484	2.962543304	1.566836242	-	Up
ENSOCUG00000022479	3.457290275	1.789641738	-	Up
ENSOCUG00000020935	2.009431652	1.006787507	MTCH1	Up
ENSOCUG00000025816	24.58584501	4.619756036	-	Up
ENSOCUG00000023753	2.282704694	1.190744235	-	Up
ENSOCUG00000004543	2.076621154	1.054238044	AIFM2	Up
ENSOCUG00000012313	3.14564096	1.653354013	-	Up
ENSOCUG00000027716	10.76191332	3.427862687	MSI1	Up
ENSOCUG00000013823	2.076577745	1.054207886	CCS	Up
ENSOCUG00000017496	Inf	Inf	VSX2	Up
ENSOCUG00000035545	Inf	Inf	SEBOX	Up
ENSOCUG00000021373	Inf	Inf	-	Up
ENSOCUG00000016747	2.021124164	1.015157954	PSMF1	Up
ENSOCUG00000003332	Inf	Inf	-	Up
ENSOCUG00000021533	4.089328438	2.031863939	-	Up
ENSOCUG00000007675	Inf	Inf	NETO1	Up
ENSOCUG00000014730	3.765975615	1.913023658	-	Up
ENSOCUG00000002991	2.049137366	1.0350167	CLCC1	Up
ENSOCUG00000037050	2.974145281	1.572475122	-	Up
ENSOCUG00000023874	2.076500138	1.054153968	-	Up
ENSOCUG00000027740	2.058282996	1.041441354	-	Up
ENSOCUG00000000564	2.019689048	1.014133192	CPNE2	Up
ENSOCUG00000005091	2.090600529	1.063917419	SERPINB9	Up
ENSOCUG00000014304	2.044956975	1.03207049	CRTC3	Up
ENSOCUG00000017408	2.055398588	1.039418192	-	Up
ENSOCUG00000025029	3.973986273	1.990586889	CSF1	Up
ENSOCUG00000001203	2.136383331	1.095170533	HIVEP3	Up
ENSOCUG00000027239	8.170449833	3.03041551	-	Up
ENSOCUG00000002135	2.053990081	1.038429215	TAP1	Up
ENSOCUG00000010380	2.028194735	1.020196178	-	Up
ENSOCUG00000039353	2.086362667	1.06098996	-	Up
ENSOCUG00000002994	2.994359818	1.582247593	CCDC3	Up
ENSOCUG00000011077	3.20220628	1.679066246	ITIH3	Up
ENSOCUG00000026402	Inf	Inf	-	Up
ENSOCUG00000022938	Inf	Inf	TRARG1	Up
ENSOCUG00000015935	2.126904454	1.088755226	TCF7	Up

ENSOCUG00000000098	2.961461588	1.566309373	SLC16A10	Up
ENSOCUG00000015548	2.001020488	1.000735939	PEAK1	Up
ENSOCUG00000006388	2.645629283	1.403610919	-	Up
ENSOCUG00000008118	3.08507836	1.625307134	LYPD6	Up
ENSOCUG00000002611	2.021544061	1.015457649	PELI2	Up
ENSOCUG00000039585	3.626948711	1.858756344	PRRT4	Up
ENSOCUG00000009743	2.033027565	1.023629776	MTMR4	Up
ENSOCUG00000026189	4.159825122	2.056522879	DUSP29	Up
ENSOCUG00000000446	2.185792154	1.128156222	BCAT2	Up
ENSOCUG00000021344	2.130622501	1.091275002	-	Up
ENSOCUG00000008758	2.036051931	1.025774359	TEAD2	Up
ENSOCUG00000010424	2.313350656	1.209983965	TNNI1	Up
ENSOCUG00000027279	6.22436791	2.637927338	CHRND	Up
ENSOCUG00000000539	2.447843249	1.291511176	KCNMB1	Up
ENSOCUG00000005379	2.07659181	1.054217657	ANXA6	Up
ENSOCUG00000007521	2.022463268	1.016113501	-	Up
ENSOCUG00000016613	0.0043741	-7.836798236	RNF222	Down
ENSOCUG00000014705	0.011869444	-6.39660379	NEU2	Down
ENSOCUG00000008547	0.011341824	-6.462203486	-	Down
ENSOCUG00000012440	0.041605745	-4.587073439	PRRG4	Down
ENSOCUG00000015830	0.035751633	-4.805847058	ASPRV1	Down
ENSOCUG00000013357	0.036802572	-4.764049577	GRHL3	Down
ENSOCUG00000015200	0.020395706	-5.615590766	C1orf68	Down
ENSOCUG00000027003	0.036878054	-4.761093644	-	Down
ENSOCUG00000033213	0.008519578	-6.875002301	-	Down
ENSOCUG00000017782	0.041133358	-4.60354732	ACE2	Down
ENSOCUG00000026830	0.04505802	-4.472072287	-	Down
ENSOCUG00000000632	0.050195424	-4.31630034	CLDN23	Down
ENSOCUG00000030919	0.03059275	-5.030666409	-	Down
ENSOCUG00000007495	0.007845106	-6.993991262	RNASE1	Down
ENSOCUG00000034595	0.036282487	-4.784582857	-	Down
ENSOCUG00000029278	0.084171009	-3.570532778	KRT78	Down
ENSOCUG00000027934	0.065423967	-3.934036959	-	Down
ENSOCUG00000005107	0.097369668	-3.360383765	HOPX	Down
ENSOCUG00000002710	0.063358312	-3.980322284	CASP14	Down
ENSOCUG00000007574	0.099914312	-3.323164843	KLK7	Down
ENSOCUG00000023532	0.027270834	-5.196497383	-	Down
ENSOCUG00000023491	0.100862318	-3.309540814	C6orf132	Down
ENSOCUG00000017320	0.075810948	-3.721449983	RNF227	Down
ENSOCUG00000002305	0.103659675	-3.270073321	ZNF750	Down
ENSOCUG00000029139	0.005968791	-7.388345623	CPA4	Down
ENSOCUG00000025918	0.079904527	-3.645578956	H2AC19	Down
ENSOCUG00000012345	0.118086216	-3.082087527	PPIF	Down
ENSOCUG00000002222	0.029636973	-5.076458062	-	Down

ENSOCUG00000017569	0.056797297	-4.138033917	SLC5A1	Down
ENSOCUG00000012034	0.12567723	-2.992204811	RBM47	Down
ENSOCUG00000006494	0.036326689	-4.782826327	CNFN	Down
ENSOCUG00000029634	0.125003834	-2.999955754	ALOXE3	Down
ENSOCUG00000007958	0.042840352	-4.544885841	LY6G6C	Down
ENSOCUG00000017247	0.043639742	-4.518213614	CYP4F22	Down
ENSOCUG00000026146	0.13227543	-2.918382986	-	Down
ENSOCUG00000025990	0.085884512	-3.541458199	PRSS27	Down
ENSOCUG00000004348	0.120199771	-3.056493944	-	Down
ENSOCUG00000017717	0.14397091	-2.796150752	GRHL1	Down
ENSOCUG00000026273	0.055210227	-4.178920658	SERPINA3	Down
ENSOCUG00000029925	0.030255616	-5.046653246	-	Down
ENSOCUG00000001183	0.063968407	-3.966496642	SH3GL2	Down
ENSOCUG00000016959	0.112860244	-3.147390717	CRYBG2	Down
ENSOCUG00000010917	0.154782625	-2.691684563	SBSN	Down
ENSOCUG00000011634	0.018969294	-5.720190227	SMPD3	Down
ENSOCUG00000009682	0.029660549	-5.075310873	GSDMA	Down
ENSOCUG00000022882	0.111555027	-3.164172573	NEURL2	Down
ENSOCUG00000012899	0.150045821	-2.736524958	-	Down
ENSOCUG00000005661	0.024730163	-5.337584423	XKRX	Down
ENSOCUG00000004655	0.068521543	-3.867298553	-	Down
ENSOCUG00000015864	0.148622603	-2.750274555	ABLIM1	Down
ENSOCUG00000008805	0.153817275	-2.700710558	CDH1	Down
ENSOCUG00000001858	0.058719785	-4.090009498	ICOS	Down
ENSOCUG00000013920	0.064464654	-3.955347833	KRT80	Down
ENSOCUG00000010998	0.038628983	-4.694172497	-	Down
ENSOCUG00000001988	0	-Inf	-	Down
ENSOCUG00000022981	0.07942892	-3.654191806	ATP6V1C2	Down
ENSOCUG00000021689	0.077326934	-3.692885176	KLK5	Down
ENSOCUG00000001242	0.158803638	-2.654684133	ESRP2	Down
ENSOCUG00000007555	0.020644549	-5.598095287	PGLYRP2	Down
ENSOCUG00000015993	0.138762057	-2.849314962	PARD6B	Down
ENSOCUG00000004502	0.137995198	-2.857310027	-	Down
ENSOCUG00000025640	0.044991959	-4.474188999	-	Down
ENSOCUG00000029538	0.114965029	-3.12073302	FLVCR2	Down
ENSOCUG00000010469	0.158729103	-2.65536142	SLC22A23	Down
ENSOCUG00000005043	0.129378262	-2.950332852	LIPH	Down
ENSOCUG00000002636	0.14340309	-2.801851981	FAM160A1	Down
ENSOCUG00000022280	0.11029491	-3.180561879	TMEM45B	Down
ENSOCUG00000022565	0.055457223	-4.172480817	-	Down
ENSOCUG00000004964	0.117910886	-3.084231177	OCLN	Down
ENSOCUG00000005959	0.027392524	-5.190073986	ALOX12B	Down
ENSOCUG00000038237	0.082925481	-3.59204072	-	Down
ENSOCUG00000027227	0.02245522	-5.476805322	H2BC3	Down

ENSOCUG00000012131	0.046608503	-4.423263023	SULT2B1	Down
ENSOCUG00000014346	0.182119166	-2.45704534	C1orf116	Down
ENSOCUG00000005907	0.018097145	-5.788094057	-	Down
ENSOCUG00000000740	0.177208714	-2.496478545	CTSA	Down
ENSOCUG00000025530	0.093812578	-3.414074817	C5AR2	Down
ENSOCUG00000031173	0.132017702	-2.921196703	-	Down
ENSOCUG00000001870	0.040401449	-4.629449165	FAM180A	Down
ENSOCUG00000032235	0.021470551	-5.541496946	LCE1B	Down
ENSOCUG00000015787	0.114895478	-3.121606076	BFSP1	Down
ENSOCUG00000017322	0.187260689	-2.416880025	-	Down
ENSOCUG00000010358	0.081257552	-3.621354296	CLDN4	Down
ENSOCUG00000005577	0.159865934	-2.645065553	C15orf62	Down
ENSOCUG00000006280	0.038502376	-4.698908718	CDSN	Down
ENSOCUG00000015828	0.189353578	-2.400845409	MPZL3	Down
ENSOCUG00000011541	0.07899426	-3.662108356	ILDR1	Down
ENSOCUG00000008623	0.091392669	-3.451777752	-	Down
ENSOCUG00000002367	0.194927416	-2.358991079	NECTIN4	Down
ENSOCUG00000012195	0.033444607	-4.902082597	SPATA25	Down
ENSOCUG00000015248	0.147076514	-2.765361205	SHISA2	Down
ENSOCUG00000029636	0.153716551	-2.701655579	TGFA	Down
ENSOCUG00000025743	0.036647916	-4.770125028	MAL2	Down
ENSOCUG00000003870	0	-Inf	FGF19	Down
ENSOCUG00000009799	0.160171129	-2.642313968	ZBTB43	Down
ENSOCUG00000021700	0.187618786	-2.414123805	-	Down
ENSOCUG00000016923	0.19683175	-2.344965143	PCDH1	Down
ENSOCUG00000004704	0.191392138	-2.385396525	BNIPL	Down
ENSOCUG00000013957	0.192170627	-2.379540258	LGALSL	Down
ENSOCUG00000026869	0.102370863	-3.288122945	OVOL1	Down
ENSOCUG00000002660	0.183310041	-2.44764228	CALHM5	Down
ENSOCUG00000011446	0.015277206	-6.032475477	LHFPL5	Down
ENSOCUG00000014955	0.067833142	-3.88186587	ANXA9	Down
ENSOCUG00000003544	0.178842127	-2.483241488	MAL	Down
ENSOCUG00000004617	0.20138571	-2.311966779	ANKRD22	Down
ENSOCUG00000002864	0.100807153	-3.310330086	BLNK	Down
ENSOCUG00000001405	0.189574771	-2.399161115	ABTB2	Down
ENSOCUG00000006223	0.114531192	-3.126187529	EPCAM	Down
ENSOCUG00000023928	0.047444405	-4.397618222	-	Down
ENSOCUG00000012411	0.14631872	-2.772813732	UHMK1	Down
ENSOCUG00000014245	0.202893095	-2.301208325	METRNL	Down
ENSOCUG00000037791	0.013621563	-6.197963923	SPINK7	Down
ENSOCUG00000022899	0.220870521	-2.178727216	LLGL2	Down
ENSOCUG00000017054	0.077721126	-3.685549396	TEX33	Down
ENSOCUG00000011525	0.092353747	-3.436685701	PPL	Down
ENSOCUG00000015660	0.089384936	-3.483824474	-	Down

ENSOCUG00000026310	0.220307777	-2.182407672	CASZ1	Down
ENSOCUG00000021306	0	-Inf	-	Down
ENSOCUG00000001098	0.032071383	-4.962569639	DTHD1	Down
ENSOCUG00000011277	0.01232816	-6.341898662	-	Down
ENSOCUG00000000592	0.200856968	-2.315759583	RDH12	Down
ENSOCUG00000016758	0.029572103	-5.079619356	-	Down
ENSOCUG00000020941	0.083035753	-3.590123541	-	Down
ENSOCUG00000008673	0.078927789	-3.663322856	ATG9B	Down
ENSOCUG00000008639	0.058327521	-4.09967944	BNIP5	Down
ENSOCUG00000002719	0.107353423	-3.219559904	EPN3	Down
ENSOCUG00000008418	0.077846012	-3.683233049	B4GALNT3	Down
ENSOCUG00000010627	0.162684421	-2.619851992	DENND1C	Down
ENSOCUG00000025593	0.079679166	-3.649653641	-	Down
ENSOCUG00000031181	0.098058321	-3.350216133	TGM1	Down
ENSOCUG00000016366	0.164674084	-2.602314569	DNAJB9	Down
ENSOCUG00000014447	0.092337335	-3.436942088	-	Down
ENSOCUG00000017861	0.043728968	-4.515266884	LRAT	Down
ENSOCUG00000010456	0.075330954	-3.730613399	RASGEF1B	Down
ENSOCUG00000029467	0.225547393	-2.148497482	TMEM154	Down
ENSOCUG00000013779	0.084352761	-3.567420901	FOXN1	Down
ENSOCUG00000037351	0.02035391	-5.618550222	-	Down
ENSOCUG00000022921	0.230170808	-2.119223223	CAMSAP3	Down
ENSOCUG00000005904	0.229263077	-2.124924067	-	Down
ENSOCUG00000025487	0.225310199	-2.150015477	MBP	Down
ENSOCUG00000007938	0.114996564	-3.120337344	-	Down
ENSOCUG00000002296	0.224423024	-2.155707406	-	Down
ENSOCUG00000000181	0.063055746	-3.98722834	GPR39	Down
ENSOCUG00000006779	0.115166235	-3.118210287	-	Down
ENSOCUG00000014482	0.166467757	-2.586685325	PLXDC2	Down
ENSOCUG00000011211	0.017888998	-5.804783606	CKMT2	Down
ENSOCUG00000033424	0	-Inf	-	Down
ENSOCUG00000017035	0.031775293	-4.975950772	-	Down
ENSOCUG00000026002	0.122200239	-3.032680988	-	Down
ENSOCUG00000001548	0.074388991	-3.748767067	RASEF	Down
ENSOCUG00000003581	0.145821278	-2.777726843	WWC1	Down
ENSOCUG00000000356	0.231518657	-2.110799634	GBA	Down
ENSOCUG00000012439	0.217029937	-2.204034031	ACER2	Down
ENSOCUG00000032580	0.091406619	-3.451557547	-	Down
ENSOCUG00000004469	0.138629614	-2.850692614	ZDHHC13	Down
ENSOCUG00000024878	0.005328817	-7.551968887	H2AC6	Down
ENSOCUG00000003500	0.170884457	-2.548906912	LRATD2	Down
ENSOCUG00000012215	0.022931508	-5.446524972	TRPV3	Down
ENSOCUG00000026264	0.237636723	-2.073170294	-	Down
ENSOCUG00000008513	0.248468768	-2.008863573	MYO1D	Down

ENSOCUG00000016556	0.070506364	-3.826102716	GPRC5A	Down
ENSOCUG00000002770	0.178594935	-2.485236925	FAM3D	Down
ENSOCUG00000036170	0.009109355	-6.778435339	-	Down
ENSOCUG00000006829	0.23028364	-2.118516174	ARHGEF37	Down
ENSOCUG00000002887	0.190687003	-2.390721581	FRMD4B	Down
ENSOCUG00000010366	0.250800838	-1.995385924	-	Down
ENSOCUG00000030473	0.131121301	-2.931026017	-	Down
ENSOCUG00000013019	0.182773204	-2.451873519	-	Down
ENSOCUG00000025548	0.11213366	-3.156708693	SLC19A2	Down
ENSOCUG00000003954	0.22184949	-2.172346858	TMEM86A	Down
ENSOCUG00000012460	0.180649277	-2.468736611	PLK3	Down
ENSOCUG00000030506	0.085552271	-3.547050044	-	Down
ENSOCUG00000001234	0.261480933	-1.935222343	NIPAL4	Down
ENSOCUG00000014777	0.046516578	-4.426111211	PADI2	Down
ENSOCUG00000001813	0.003802109	-8.038984359	ARG1	Down
ENSOCUG00000005558	0.052294038	-4.257209708	PXDNL	Down
ENSOCUG00000015820	0.064303675	-3.958955006	MXD1	Down
ENSOCUG00000005502	0.033382049	-4.90478369	PRR9	Down
ENSOCUG00000004720	0.010890852	-6.520739381	TMPRSS11F	Down
ENSOCUG00000009365	0.185847214	-2.427811037	ST14	Down
ENSOCUG00000017926	0.010416613	-6.584969953	ABCA12	Down
ENSOCUG00000013892	0.266013013	-1.910431272	TACSTD2	Down
ENSOCUG00000007002	0.174034935	-2.522551158	-	Down
ENSOCUG00000002358	0.209496492	-2.255002011	ARHGAP30	Down
ENSOCUG00000022624	0.10512567	-3.249813102	SLC26A9	Down
ENSOCUG00000010367	0.094798628	-3.39899001	ZDHHC21	Down
ENSOCUG00000007342	0	-Inf	-	Down
ENSOCUG00000006574	0.007719185	-7.0173357	PLA2G2E	Down
ENSOCUG00000012534	0.041442774	-4.592735601	DSC1	Down
ENSOCUG00000015495	0.151059016	-2.726815799	PRDM1	Down
ENSOCUG00000011294	0.217287679	-2.202321726	-	Down
ENSOCUG00000022883	0.134238059	-2.897134332	GDPD3	Down
ENSOCUG00000001326	0.244312761	-2.033198875	SEMA4A	Down
ENSOCUG00000005054	0.046778849	-4.41799984	RNF183	Down
ENSOCUG00000012016	0.016602473	-5.912457996	SEMA3D	Down
ENSOCUG00000031729	0.036099945	-4.791859548	LCE1C	Down
ENSOCUG00000009796	0.006760934	-7.208561735	AQP9	Down
ENSOCUG00000024652	0.086262721	-3.535118971	THAP5	Down
ENSOCUG00000017211	0.186967822	-2.4191381	HMBOX1	Down
ENSOCUG00000024109	0.054220797	-4.205009867	-	Down
ENSOCUG00000027315	0.051726654	-4.272948311	-	Down
ENSOCUG00000023717	0.201247199	-2.312959389	CNKSR1	Down
ENSOCUG00000033572	0.152672817	-2.711484876	-	Down
ENSOCUG00000010604	0.276584417	-1.854208218	-	Down

ENSOCUG00000010152	0.275503882	-1.859855448	SCNN1A	Down
ENSOCUG00000029487	0.005212826	-7.583718637	C6orf15	Down
ENSOCUG00000003828	0.056241804	-4.152213314	CERS3	Down
ENSOCUG00000029033	0.207865833	-2.266275455	-	Down
ENSOCUG00000016270	0.101290635	-3.303427306	EPGN	Down
ENSOCUG00000022857	0.250821689	-1.995265991	EFNB2	Down
ENSOCUG00000008628	0.230404554	-2.117758861	PNPLA1	Down
ENSOCUG00000015475	0.130941577	-2.933004833	USP43	Down
ENSOCUG00000003423	0.033340758	-4.906569278	SDR9C7	Down
ENSOCUG00000007853	0.27226596	-1.876911474	-	Down
ENSOCUG00000000574	0.183948479	-2.442626349	PLEKHH1	Down
ENSOCUG00000016524	0.259464176	-1.946392735	TIAM1	Down
ENSOCUG00000016867	0.037775613	-4.726401036	SYT16	Down
ENSOCUG00000007759	0.250851677	-1.995093512	USP2	Down
ENSOCUG00000005614	0.271167628	-1.882743135	TTC9	Down
ENSOCUG00000011842	0.050816054	-4.298571838	CRNN	Down
ENSOCUG00000015653	0.124094923	-3.010484007	RNF39	Down
ENSOCUG00000029474	0.262833861	-1.927776943	-	Down
ENSOCUG00000033562	0	-Inf	H2BU1	Down
ENSOCUG00000025569	0.215808255	-2.212178046	CCR7	Down
ENSOCUG00000027145	0	-Inf	-	Down
ENSOCUG00000027528	0.273678865	-1.869444069	PHACTR4	Down
ENSOCUG00000034297	0.263843917	-1.922243375	-	Down
ENSOCUG00000016129	0.225752058	-2.147188953	-	Down
ENSOCUG00000017169	0.029247981	-5.095519167	-	Down
ENSOCUG00000013921	0.189904958	-2.396650526	NXPH3	Down
ENSOCUG00000033658	0.105496199	-3.244737076	PAQR5	Down
ENSOCUG00000026068	0.271161795	-1.882774167	LDLRAD3	Down
ENSOCUG00000038970	0.013676902	-6.19211468	RNF225	Down
ENSOCUG00000037250	0	-Inf	-	Down
ENSOCUG00000005298	0.284639047	-1.812794508	-	Down
ENSOCUG00000009917	0.100201428	-3.319025026	DNAH3	Down
ENSOCUG00000022858	0.034919387	-4.839827965	RAB27B	Down
ENSOCUG00000014669	0.268783249	-1.895484865	TRMT6	Down
ENSOCUG00000004549	0.227594625	-2.135461608	CYP51A1	Down
ENSOCUG00000001049	0.085530797	-3.547412198	SLC5A7	Down
ENSOCUG00000004730	0.10958733	-3.189847086	SYBU	Down
ENSOCUG00000003316	0.052580075	-4.24934	-	Down
ENSOCUG00000007812	0.143265986	-2.80323197	KPNA5	Down
ENSOCUG00000001100	0.266354435	-1.908580792	SOX15	Down
ENSOCUG00000008414	0.269895166	-1.889528956	RDH10	Down
ENSOCUG00000012070	0.042022872	-4.572681428	SPNS2	Down
ENSOCUG00000025069	0.047449723	-4.397456514	-	Down
ENSOCUG00000025273	0.022939835	-5.44600116	-	Down

ENSOCUG00000024702	0.027567559	-5.180884657	-	Down
ENSOCUG00000006863	0.070060257	-3.835259907	SERPINB8	Down
ENSOCUG00000014760	0.187620796	-2.414108346	MEGF11	Down
ENSOCUG00000005334	0.164524519	-2.603625495	ELOVL4	Down
ENSOCUG00000010635	0.201347699	-2.31223911	-	Down
ENSOCUG00000014548	0.304621236	-1.714911577	EVPL	Down
ENSOCUG00000002384	0.287149712	-1.800124979	NPAS2	Down
ENSOCUG00000000085	0.276546333	-1.854406882	TM2D2	Down
ENSOCUG00000023091	0.013130514	-6.25093284	-	Down
ENSOCUG00000004511	0.295895781	-1.75683897	HSPB8	Down
ENSOCUG00000007602	0.055981164	-4.158914705	HS3ST1	Down
ENSOCUG00000011984	0.283732968	-1.817394304	-	Down
ENSOCUG00000006632	0.126332337	-2.98470412	MREG	Down
ENSOCUG00000006562	0.019209185	-5.702059887	LANCL3	Down
ENSOCUG00000004094	0.295957565	-1.756537761	OSER1	Down
ENSOCUG00000016641	0.218871353	-2.191844955	-	Down
ENSOCUG00000007502	0.298056824	-1.746340693	ELF3	Down
ENSOCUG00000014604	0.059827878	-4.063038289	TMPRSS11E	Down
ENSOCUG00000002304	0.291547699	-1.778196157	CLIP1	Down
ENSOCUG00000025659	0	-Inf	-	Down
ENSOCUG00000010693	0.13319328	-2.908406795	CCN3	Down
ENSOCUG00000026551	0.300557242	-1.734288312	LPAR5	Down
ENSOCUG00000005139	0.233875288	-2.096188667	HIVEP2	Down
ENSOCUG00000014425	0.289075897	-1.790479772	FILIP1L	Down
ENSOCUG00000008806	0.04375807	-4.514307066	SPINK6	Down
ENSOCUG00000008599	0.094593825	-3.402110179	DLX5	Down
ENSOCUG00000008893	0.309289636	-1.692969604	EMC7	Down
ENSOCUG00000036368	0	-Inf	-	Down
ENSOCUG00000004595	0.293683732	-1.767664744	LLPH	Down
ENSOCUG00000002306	0.040928512	-4.610749976	PRSS8	Down
ENSOCUG00000008863	0.31072966	-1.686268139	PNRC1	Down
ENSOCUG00000025017	0.27202327	-1.878198022	-	Down
ENSOCUG00000013004	0.039041863	-4.678834283	ADGRF2	Down
ENSOCUG00000010272	0.162044654	-2.625536668	ACP3	Down
ENSOCUG00000023757	0.038757239	-4.689390392	NOS3	Down
ENSOCUG00000015511	0.04391817	-4.509038247	ABHD12B	Down
ENSOCUG00000007272	0.057126233	-4.129702797	CCDC186	Down
ENSOCUG00000017009	0.154181036	-2.697302764	-	Down
ENSOCUG00000007366	0.082799827	-3.594228428	ENDOU	Down
ENSOCUG00000004238	0.290223675	-1.784762881	-	Down
ENSOCUG00000026883	0.211104961	-2.24396761	DNASE1L2	Down
ENSOCUG00000015007	0.141501973	-2.821105923	SPNS3	Down
ENSOCUG00000013854	0.09698506	-3.366093667	CEP85L	Down
ENSOCUG00000001029	0.038641222	-4.693715471	CDH17	Down

ENSOCUG00000016969	0.252395856	-1.986239871	AMMECR1	Down
ENSOCUG00000025279	0.264398524	-1.919213973	C9orf152	Down
ENSOCUG00000015524	0.195270808	-2.356451804	PAPPA2	Down
ENSOCUG00000012518	0.066062589	-3.920022675	DSC2	Down
ENSOCUG00000020975	0.327259904	-1.61149124	CSTB	Down
ENSOCUG00000005200	0	-Inf	-	Down
ENSOCUG00000029247	0.091650994	-3.447705668	-	Down
ENSOCUG00000004924	0.306966849	-1.703845237	NHLRC2	Down
ENSOCUG00000007526	0.317434343	-1.655469874	CTH	Down
ENSOCUG00000024510	0.086028063	-3.539048838	-	Down
ENSOCUG00000004035	0.32595799	-1.617242056	IRF6	Down
ENSOCUG00000014188	0.040439133	-4.628104121	NPY4R	Down
ENSOCUG00000017694	0.21510272	-2.216902328	KRT23	Down
ENSOCUG00000008078	0.320180405	-1.643043078	SPTLC1	Down
ENSOCUG00000023535	0.2898818	-1.786463335	H2BC21	Down
ENSOCUG00000002886	0.236900134	-2.077649078	ZNF329	Down
ENSOCUG00000022674	0.319284836	-1.647084058	CLN8	Down
ENSOCUG00000015182	0.297147908	-1.750746869	-	Down
ENSOCUG00000002830	0.325100118	-1.621044016	TNS4	Down
ENSOCUG00000001575	0.322139755	-1.634241381	SLC9A3R1	Down
ENSOCUG00000014298	0.310954713	-1.685223613	DUOXA1	Down
ENSOCUG00000017147	0.126456111	-2.98329134	CDS1	Down
ENSOCUG00000001073	0.310458383	-1.687528208	GAN	Down
ENSOCUG00000023433	0.093629055	-3.416899894	ITSN2	Down
ENSOCUG00000004324	0.116856083	-3.097195264	ZFP42	Down
ENSOCUG00000024282	0.208544086	-2.261575695	PLEKHB2	Down
ENSOCUG00000001434	0.315183821	-1.665734616	SPTLC2	Down
ENSOCUG00000004887	0.335920702	-1.573807386	RHOV	Down
ENSOCUG00000012640	0.300093127	-1.736517818	CGN	Down
ENSOCUG00000005255	0.093992766	-3.411306463	-	Down
ENSOCUG00000002944	0.109739558	-3.187844431	SLC34A2	Down
ENSOCUG00000002885	0.316989053	-1.657495075	AKTIP	Down
ENSOCUG00000024543	0.06261809	-3.99727668	H2BC18	Down
ENSOCUG00000027895	0.33761006	-1.566570203	-	Down
ENSOCUG00000011835	0.215172473	-2.216434571	CXCL8	Down
ENSOCUG00000002283	0.336826187	-1.569923787	LRATD1	Down
ENSOCUG00000026281	0.322612298	-1.632126659	PAK6	Down
ENSOCUG00000003434	0.318484229	-1.650706161	SLC49A4	Down
ENSOCUG00000005865	0.257801538	-1.955667222	-	Down
ENSOCUG00000001009	0.26851879	-1.896905046	NFE2L2	Down
ENSOCUG00000009371	0.331624302	-1.59237836	RMND5A	Down
ENSOCUG00000008971	0.132058309	-2.920753019	CPEB4	Down
ENSOCUG00000010144	0.131522403	-2.926619527	TTC22	Down
ENSOCUG00000025115	0.262956622	-1.927103268	MPHOSPH10	Down

ENSOCUG00000006847	0.241971224	-2.04709261	CHMP4C	Down
ENSOCUG00000010489	0.042965885	-4.540664594	NLRP10	Down
ENSOCUG00000031994	0.004739583	-7.721024063	TMEM238L	Down
ENSOCUG00000036714	0.282604035	-1.823146033	MPIG6B	Down
ENSOCUG00000008619	0.187735798	-2.413224318	TMCC3	Down
ENSOCUG00000027529	0.133094893	-2.909472884	ELOVL7	Down
ENSOCUG00000014207	0.065472253	-3.932972554	ELF5	Down
ENSOCUG00000009943	0.333272213	-1.585227058	SLC38A2	Down
ENSOCUG00000010159	0.269673785	-1.89071281	FADS6	Down
ENSOCUG00000002199	0.057893331	-4.110459017	GATA4	Down
ENSOCUG00000017863	0.166213109	-2.588893927	RRP15	Down
ENSOCUG00000000368	0.188349684	-2.408514481	THAP6	Down
ENSOCUG00000005942	0.331220065	-1.594138022	KIF13B	Down
ENSOCUG00000035346	0.089073168	-3.488865277	ARL5A	Down
ENSOCUG00000015014	0.335747014	-1.574553527	KIF3C	Down
ENSOCUG00000030000	0.018247241	-5.776177815	-	Down
ENSOCUG00000003383	0.139469133	-2.84198223	MGAT4A	Down
ENSOCUG00000005414	0.08474521	-3.56072436	ESF1	Down
ENSOCUG00000017130	0.33603755	-1.573305643	TMEM33	Down
ENSOCUG00000009573	0.326704331	-1.613942514	C18orf25	Down
ENSOCUG00000017132	0.09261639	-3.432588662	-	Down
ENSOCUG00000023564	0.042756781	-4.547702964	-	Down
ENSOCUG00000016332	0.245796079	-2.024466196	CDC73	Down
ENSOCUG00000007258	0.10407643	-3.264284711	STRN	Down
ENSOCUG00000012416	0.196569315	-2.346889966	STON2	Down
ENSOCUG00000002008	0.285406798	-1.808908397	STK36	Down
ENSOCUG00000008346	0.330724219	-1.596299395	LYPD6B	Down
ENSOCUG00000015909	0.268494326	-1.897036497	HBEGF	Down
ENSOCUG00000013509	0.042060485	-4.571390718	SKIL	Down
ENSOCUG00000011318	0.346802941	-1.527811963	KLF5	Down
ENSOCUG00000007681	0.036509947	-4.775566628	FAM214A	Down
ENSOCUG00000021026	0.179546703	-2.477568937	-	Down
ENSOCUG00000014159	0.338404632	-1.563178777	KDSR	Down
ENSOCUG00000003710	0.338075681	-1.564581852	SPPL2A	Down
ENSOCUG00000010656	0.320763561	-1.640417836	TTC39B	Down
ENSOCUG00000001054	0.288599832	-1.792857636	-	Down
ENSOCUG00000005205	0.169092049	-2.56411927	-	Down
ENSOCUG00000014349	0.089402995	-3.483533022	YOD1	Down
ENSOCUG00000016248	0.292548252	-1.773253497	GRAMD2B	Down
ENSOCUG00000017859	0.027903272	-5.16342186	RBM46	Down
ENSOCUG00000016492	0.331948159	-1.590970146	-	Down
ENSOCUG00000000121	0.300921137	-1.73254265	LRR1	Down
ENSOCUG00000024341	0.33298332	-1.586478182	-	Down
ENSOCUG00000008386	0.027588786	-5.179774219	GPA1	Down

ENSOCUG00000010690	0.086588316	-3.529683832	FAM126B	Down
ENSOCUG00000001456	0.346310518	-1.52986189	-	Down
ENSOCUG00000000068	0.149966431	-2.737288494	FRMPD1	Down
ENSOCUG00000032147	0.346024149	-1.531055366	-	Down
ENSOCUG00000012063	0.355031782	-1.493979917	JUP	Down
ENSOCUG00000010996	0.345047834	-1.535131717	TFAP2C	Down
ENSOCUG00000027657	0.352035216	-1.50620834	GRB7	Down
ENSOCUG00000011082	0.359189343	-1.477183546	EPHA1	Down
ENSOCUG00000025223	0.289421067	-1.788758155	PLCXD1	Down
ENSOCUG00000039679	0.327586882	-1.610050508	-	Down
ENSOCUG00000004644	0.347854887	-1.523442508	CCDC120	Down
ENSOCUG00000029885	0.271074631	-1.88323799	-	Down
ENSOCUG00000005658	0.104426837	-3.25943557	PDZRN4	Down
ENSOCUG00000006552	0.156958512	-2.671544828	DNAJB14	Down
ENSOCUG00000016740	0.162724246	-2.619498867	DPF3	Down
ENSOCUG00000034283	0.295888241	-1.75687573	-	Down
ENSOCUG00000009236	0.076464435	-3.709067304	FAM135A	Down
ENSOCUG00000017700	0.023532264	-5.409216091	PLS1	Down
ENSOCUG00000000831	0.060721949	-4.041638089	DNASE1L3	Down
ENSOCUG00000011674	0.269544429	-1.891405002	BMP2	Down
ENSOCUG00000016088	0.34090713	-1.552549323	ADRB2	Down
ENSOCUG00000010667	0.338204311	-1.564033045	-	Down
ENSOCUG00000007130	0.346469341	-1.529200403	SLC25A25	Down
ENSOCUG00000003140	0.215113691	-2.21682875	NFE2L3	Down
ENSOCUG00000015638	0.013862813	-6.172636133	ZNF518A	Down
ENSOCUG00000015801	0.024159512	-5.371264888	-	Down
ENSOCUG00000016138	0.332983502	-1.586477395	-	Down
ENSOCUG00000010633	0.181014215	-2.465825101	HMOX1	Down
ENSOCUG00000007932	0.153987867	-2.699111409	ZNF655	Down
ENSOCUG00000014443	0.114650519	-3.124685213	SERPINB7	Down
ENSOCUG00000004386	0.108555997	-3.203488666	NFAT5	Down
ENSOCUG00000011658	0.133523256	-2.904837053	-	Down
ENSOCUG00000013646	0.345444458	-1.533474327	-	Down
ENSOCUG00000009614	0.229393917	-2.124100962	PHACTR2	Down
ENSOCUG00000003035	0.09410451	-3.40959232	SLK	Down
ENSOCUG00000007590	0.038440598	-4.701225421	SUCO	Down
ENSOCUG00000011595	0.347788814	-1.523716563	USP14	Down
ENSOCUG00000028058	0.134947355	-2.889531395	CHAC2	Down
ENSOCUG00000008771	0.249366472	-2.003660592	IL1A	Down
ENSOCUG00000012418	0.07474345	-3.741909027	FGF5	Down
ENSOCUG00000007950	0.278514358	-1.844176391	CPEB2	Down
ENSOCUG00000008291	0.21789665	-2.198284079	BIN2	Down
ENSOCUG00000033697	0	-Inf	-	Down
ENSOCUG00000017131	0.028483702	-5.133719506	LMOD2	Down

ENSOCUG00000003283	0.084129383	-3.571246433	FCHO2	Down
ENSOCUG00000014407	0.330510113	-1.59723368	NUP35	Down
ENSOCUG00000007506	0.35189174	-1.506796443	MARCHF3	Down
ENSOCUG00000002771	0.256957021	-1.960401024	TOB1	Down
ENSOCUG00000009672	0.15931641	-2.650033222	-	Down
ENSOCUG00000013448	0.340614428	-1.553788548	CNOT4	Down
ENSOCUG00000024437	0.317791464	-1.653847723	-	Down
ENSOCUG00000022118	0.361712911	-1.467083001	PSPH	Down
ENSOCUG00000009850	0.271759238	-1.879599016	NPC1	Down
ENSOCUG00000003400	0.357023358	-1.485909628	ZFP36	Down
ENSOCUG00000023781	0.200184861	-2.32059522	-	Down
ENSOCUG00000004320	0.065557094	-3.931104293	TMTC3	Down
ENSOCUG00000026176	0.349887141	-1.515038453	LTB4R	Down
ENSOCUG00000015756	0.028961836	-5.109703115	KNL1	Down
ENSOCUG00000015839	0.361045163	-1.46974878	FAM83G	Down
ENSOCUG00000015554	0.34968603	-1.515867936	SELENOI	Down
ENSOCUG00000003854	0.145033456	-2.785542356	TPMT	Down
ENSOCUG00000038545	0	-Inf	LY6G6D	Down
ENSOCUG00000007047	0.354545405	-1.495957696	ZDHHC5	Down
ENSOCUG00000003405	0.230330568	-2.118222208	MFSD6	Down
ENSOCUG00000005652	0.126799163	-2.979382875	NOX1	Down
ENSOCUG00000007348	0.126507593	-2.982704121	-	Down
ENSOCUG00000003729	0.314341387	-1.66959586	NFRKB	Down
ENSOCUG00000013737	0.187805974	-2.41268514	RBM25	Down
ENSOCUG00000003240	0.352116151	-1.505876691	IRX5	Down
ENSOCUG00000003961	0.297941306	-1.746899947	TXNRD1	Down
ENSOCUG00000001336	0.356918682	-1.486332679	ARHGAP27	Down
ENSOCUG00000017282	0.364640646	-1.455452711	LMTK2	Down
ENSOCUG00000017365	0.171677875	-2.542223974	RBMS1	Down
ENSOCUG00000012746	0.094544856	-3.402857229	STAG1	Down
ENSOCUG00000012207	0.3137305	-1.672402304	NEBL	Down
ENSOCUG00000012626	0.029252959	-5.095273609	TMPRSS11A	Down
ENSOCUG00000025993	0.164586943	-2.603078209	GIN1	Down
ENSOCUG00000010003	0.351241045	-1.50946665	UTP3	Down
ENSOCUG00000016211	0.104319553	-3.260918508	SNTB1	Down
ENSOCUG00000017017	0.314601586	-1.668402151	CARNMT1	Down
ENSOCUG00000015478	0.067754928	-3.883530311	CCL22	Down
ENSOCUG00000026452	0.108439473	-3.205038092	-	Down
ENSOCUG00000015577	0.13656757	-2.872313163	-	Down
ENSOCUG00000006922	0.062696207	-3.995478027	JMJD1C	Down
ENSOCUG00000012979	0.159226327	-2.650849203	NPTX1	Down
ENSOCUG00000010085	0	-Inf	-	Down
ENSOCUG00000037766	0.091198906	-3.454839671	-	Down
ENSOCUG00000039627	0.095096368	-3.394465949	-	Down

ENSOCUG00000014405	0.219600648	-2.187045782	TFAM	Down
ENSOCUG00000002929	0.244251002	-2.033563616	CATSPER4	Down
ENSOCUG00000003769	0.369588926	-1.436006564	MSMO1	Down
ENSOCUG00000011578	0.18513324	-2.433364147	BMP2K	Down
ENSOCUG00000010208	0.213229779	-2.229519158	DNAJC2	Down
ENSOCUG00000007730	0.375539447	-1.412963639	LAD1	Down
ENSOCUG00000006225	0.256654391	-1.962101149	-	Down
ENSOCUG00000011072	0.327131978	-1.612055303	CFLAR	Down
ENSOCUG00000030494	0.332582179	-1.588217227	RSRC1	Down
ENSOCUG00000029105	0.169820761	-2.55791525	ND3	Down
ENSOCUG00000003925	0.180227295	-2.472110574	KRR1	Down
ENSOCUG00000026691	0.368552964	-1.440056135	SLC31A2	Down
ENSOCUG00000000383	0.113219829	-3.142801441	PLA2G4E	Down
ENSOCUG00000016771	0.254184862	-1.976049982	PTGS2	Down
ENSOCUG00000008779	0.051863111	-4.269147443	SPINK5	Down
ENSOCUG00000031187	0.082675091	-3.596403455	-	Down
ENSOCUG00000013829	0.046561632	-4.424714567	KCTD4	Down
ENSOCUG00000003129	0.361560661	-1.46769038	WDR36	Down
ENSOCUG00000012071	0.346688009	-1.528290155	GPAT3	Down
ENSOCUG00000002127	0.334691977	-1.579094128	ZNF800	Down
ENSOCUG00000011673	0.298207649	-1.74561083	-	Down
ENSOCUG00000000776	0.304189235	-1.716958995	LEMD3	Down
ENSOCUG00000014986	0.219800953	-2.185730456	ASH1L	Down
ENSOCUG00000005888	0.074822966	-3.740375031	NIPBL	Down
ENSOCUG00000014876	0.093810513	-3.414106585	ARL5B	Down
ENSOCUG00000015173	0.349442326	-1.516873729	ZC3H13	Down
ENSOCUG00000029614	0.358238885	-1.48100615	-	Down
ENSOCUG00000014408	0.068451937	-3.868764817	TASOR2	Down
ENSOCUG00000005142	0.131338308	-2.928640324	NPSR1	Down
ENSOCUG00000003822	0.09999888	-3.321944254	FAM83B	Down
ENSOCUG00000005329	0.008301385	-6.912432163	APOB	Down
ENSOCUG00000003863	0.04010088	-4.640222291	CD74	Down
ENSOCUG00000009046	0.370093146	-1.434039676	GFOD2	Down
ENSOCUG00000009879	0.070560027	-3.825005076	STAG2	Down
ENSOCUG00000009745	0.240312688	-2.057015275	MEGF9	Down
ENSOCUG00000014485	0.370677651	-1.431762959	SPTBN2	Down
ENSOCUG00000011991	0.110894235	-3.172743727	PANK3	Down
ENSOCUG00000007844	0.209027435	-2.258235785	SOS1	Down
ENSOCUG00000012117	0.071591693	-3.804063989	LTN1	Down
ENSOCUG00000022586	0.015351012	-6.025522438	-	Down
ENSOCUG00000004643	0.294894126	-1.761731007	WSB1	Down
ENSOCUG00000013642	0.060634879	-4.043708275	DOCK11	Down
ENSOCUG00000011397	0.107210068	-3.2214877	-	Down
ENSOCUG00000001662	0.100091527	-3.320608244	SLC7A11	Down

ENSOCUG00000024654	0.026477234	-5.239103795	-	Down
ENSOCUG00000026343	0	-Inf	VTCN1	Down
ENSOCUG00000015408	0.28513498	-1.810283058	-	Down
ENSOCUG00000006789	0.331095854	-1.594679149	UCHL3	Down
ENSOCUG00000006495	0.366487993	-1.448162164	ABHD5	Down
ENSOCUG00000016627	0.235792638	-2.084409418	LSR	Down
ENSOCUG00000027642	0.28632882	-1.804255203	DNAJC24	Down
ENSOCUG00000026643	0.17913865	-2.480851453	UGT2B16	Down
ENSOCUG00000016605	0.263431324	-1.924501189	AKR1B1	Down
ENSOCUG00000003698	0.376599827	-1.408895759	KLF3	Down
ENSOCUG00000013557	0.235208982	-2.087984943	WNK1	Down
ENSOCUG00000027838	0.053007713	-4.237653896	-	Down
ENSOCUG00000039636	0.066789538	-3.904234046	-	Down
ENSOCUG00000005466	0	-Inf	LRRC66	Down
ENSOCUG00000017458	0.006909193	-7.177267122	NEB	Down
ENSOCUG00000009402	0.146378447	-2.772224954	-	Down
ENSOCUG00000007233	0.03528092	-4.824967999	-	Down
ENSOCUG00000010548	0.041969589	-4.57451187	ARHGAP5	Down
ENSOCUG00000009623	0.365638286	-1.451510951	-	Down
ENSOCUG00000006660	0.190023708	-2.395748667	IL36G	Down
ENSOCUG00000006776	0.364411974	-1.456357731	ODR4	Down
ENSOCUG00000011218	0.248781363	-2.007049683	CFAP92	Down
ENSOCUG00000007779	0.102533815	-3.285828317	USP16	Down
ENSOCUG00000017789	0.384090508	-1.380481782	-	Down
ENSOCUG00000017765	0.292515383	-1.773415597	PPP4R3A	Down
ENSOCUG00000025320	0.301444296	-1.730036663	-	Down
ENSOCUG00000003985	0.266344628	-1.908633911	CHMP2B	Down
ENSOCUG00000007468	0.263667917	-1.923206062	CCPG1	Down
ENSOCUG00000000186	0.210866051	-2.245601254	-	Down
ENSOCUG00000013639	0.16008504	-2.643089599	ADGRL2	Down
ENSOCUG00000009516	0.008585763	-6.863837894	CHRNA6	Down
ENSOCUG00000004218	0.27349026	-1.870438641	CD14	Down
ENSOCUG00000001540	0.121178297	-3.044796759	DUSP5	Down
ENSOCUG00000007416	0.163121914	-2.61597749	QPCT	Down
ENSOCUG00000000730	0.379025638	-1.399632656	BAIAP2L1	Down
ENSOCUG00000029499	0.200587555	-2.317695997	CWC27	Down
ENSOCUG00000001316	0.227393529	-2.136736897	ESRP1	Down
ENSOCUG00000011358	0.379096183	-1.399364164	CAB39	Down
ENSOCUG00000032815	0.324727712	-1.622697587	C3orf62	Down
ENSOCUG00000017493	0.138869098	-2.848202498	KIF5B	Down
ENSOCUG00000014500	0.095405525	-3.389783375	AHCTF1	Down
ENSOCUG00000003596	0.380029907	-1.395815139	GDE1	Down
ENSOCUG00000006047	0.357601262	-1.483576268	MARVELD2	Down
ENSOCUG00000039408	0.342790221	-1.544602143	-	Down

ENSOCUG00000009461	0.211018243	-2.244560368	ESYT3	Down
ENSOCUG00000016363	0.110372781	-3.179543657	BCLAF1	Down
ENSOCUG00000002577	0.26474424	-1.917328799	LRIF1	Down
ENSOCUG00000027150	0	-Inf	-	Down
ENSOCUG00000007494	0	-Inf	RNASE6	Down
ENSOCUG00000016401	0.095019197	-3.395637168	PHIP	Down
ENSOCUG00000039230	0	-Inf	-	Down
ENSOCUG00000003486	0	-Inf	SLC5A8	Down
ENSOCUG00000032647	0.36639627	-1.448523279	UFM1	Down
ENSOCUG00000006556	0.364728341	-1.455105788	OGFRL1	Down
ENSOCUG00000029107	0.105031624	-3.25110432	ND4L	Down
ENSOCUG00000007833	0.358661162	-1.479306564	DLX3	Down
ENSOCUG00000025092	0.086198327	-3.536196316	-	Down
ENSOCUG00000001806	0.135061704	-2.888309425	MOB1B	Down
ENSOCUG00000003795	0.093178163	-3.423864297	ERBIN	Down
ENSOCUG00000012009	0.08358175	-3.580668217	MPP5	Down
ENSOCUG00000006566	0.091206729	-3.454715927	XK	Down
ENSOCUG00000026362	0.082576439	-3.598125991	-	Down
ENSOCUG00000004404	0.247307765	-2.015620557	MCTP2	Down
ENSOCUG00000000601	0.302063277	-1.727077295	-	Down
ENSOCUG00000004930	0.266689997	-1.906764383	RC3H1	Down
ENSOCUG00000016898	0.280090756	-1.836033727	PLEKHG1	Down
ENSOCUG00000005996	0.116277957	-3.104350467	THOC2	Down
ENSOCUG00000012427	0.284543933	-1.813276677	TMED5	Down
ENSOCUG00000017435	0.064905662	-3.945511863	RIF1	Down
ENSOCUG00000002977	0.297791733	-1.747624391	-	Down
ENSOCUG00000005111	0.086492032	-3.531288951	N4BP2	Down
ENSOCUG00000016453	0.380601401	-1.393647222	TOR1AIP2	Down
ENSOCUG00000009587	0.054661476	-4.193331782	ESCO1	Down
ENSOCUG00000014998	0.075129307	-3.734480403	IL36A	Down
ENSOCUG00000038200	0.111177094	-3.169068511	-	Down
ENSOCUG00000008680	0.072605061	-3.783786079	MAP9	Down
ENSOCUG00000012638	0.330464329	-1.597433542	PSMC6	Down
ENSOCUG00000010248	0.094582909	-3.402276672	GBA2	Down
ENSOCUG00000000155	0.200816891	-2.316047475	NSRP1	Down
ENSOCUG00000003170	0.248112241	-2.010935181	SBDS	Down
ENSOCUG00000007073	0.135351009	-2.885222448	LARP7	Down
ENSOCUG00000021154	0.179486055	-2.478056333	EIF5B	Down
ENSOCUG00000003085	0.174773505	-2.516441601	KCNRG	Down
ENSOCUG00000015371	0.390408157	-1.356944897	SPINT1	Down
ENSOCUG00000017924	0.033808974	-4.886449949	MME	Down
ENSOCUG00000025054	0.068758552	-3.862317033	TREX2	Down
ENSOCUG00000003816	0.083979504	-3.573818917	HECTD1	Down
ENSOCUG00000014431	0.344931577	-1.535617887	FAM13B	Down

ENSOCUG00000014773	0.173241367	-2.529144637	HSPH1	Down
ENSOCUG00000005209	0.089441233	-3.482916111	-	Down
ENSOCUG00000011147	0.312101392	-1.679913302	CLCN1	Down
ENSOCUG00000006605	0.19007687	-2.395345109	ARID4B	Down
ENSOCUG00000008043	0.186666182	-2.421467515	ERC2	Down
ENSOCUG00000008748	0.329349928	-1.60230686	NT5DC4	Down
ENSOCUG00000001879	0.372128134	-1.42612863	SPAG1	Down
ENSOCUG00000010028	0.156416001	-2.676539992	LIN9	Down
ENSOCUG00000002557	0.247154675	-2.016513897	CLSTN2	Down
ENSOCUG00000015407	0.160780177	-2.636838555	GRAMD1C	Down
ENSOCUG00000008834	0.164991692	-2.599534711	COMMD8	Down
ENSOCUG00000007729	0.393477175	-1.345648146	-	Down
ENSOCUG00000034289	0.389678365	-1.35964426	-	Down
ENSOCUG00000006993	0.020117031	-5.635438793	CPA5	Down
ENSOCUG00000031190	0.073092586	-3.774131108	-	Down
ENSOCUG00000004968	0.124595224	-3.004679329	REL	Down
ENSOCUG00000016531	0.080189408	-3.640444495	-	Down
ENSOCUG00000008363	0.237405349	-2.074575654	VPS37A	Down
ENSOCUG00000023887	0.387823403	-1.366528231	-	Down
ENSOCUG00000012516	0.186241738	-2.424751666	LIN54	Down
ENSOCUG00000007899	0.367539035	-1.444030612	NSDHL	Down
ENSOCUG00000008191	0.13137754	-2.928209439	GABRP	Down
ENSOCUG00000004713	0.347102	-1.526568417	WAC	Down
ENSOCUG00000011242	0.38899284	-1.362184496	MFSD5	Down
ENSOCUG00000001328	0.105539762	-3.244141466	VPS13A	Down
ENSOCUG00000015579	0.395216568	-1.339284668	UAP1	Down
ENSOCUG00000028032	0.39720182	-1.332055861	TUBA1C	Down
ENSOCUG00000001309	0.196894852	-2.344502702	ISM1	Down
ENSOCUG00000013733	0.21806893	-2.197143865	USP53	Down
ENSOCUG00000016393	0.098915318	-3.33766224	RB1CC1	Down
ENSOCUG00000002984	0.2138671	-2.225213533	DOCK9	Down
ENSOCUG00000017454	0.29297882	-1.771131723	NMD3	Down
ENSOCUG00000027324	0.364699563	-1.455219625	TAF3	Down
ENSOCUG00000012735	0.299388488	-1.739909348	COQ10B	Down
ENSOCUG00000005026	0.08432378	-3.567916647	-	Down
ENSOCUG00000021508	0.403691654	-1.308674333	INAVA	Down
ENSOCUG00000007550	0.092004772	-3.442147493	MIS18BP1	Down
ENSOCUG00000014569	0.24653155	-2.020155808	OTUD4	Down
ENSOCUG00000002363	0.129137048	-2.953025147	CWC22	Down
ENSOCUG00000029308	0.396472902	-1.334705831	RAB10	Down
ENSOCUG00000014147	0.137385352	-2.863699904	CHORDC1	Down
ENSOCUG00000002674	0	-Inf	KCNG3	Down
ENSOCUG00000028097	0.33952245	-1.558421122	-	Down
ENSOCUG00000015400	0.395221502	-1.339266657	PTPN14	Down

ENSOCUG00000013300	0.110869762	-3.173062144	-	Down
ENSOCUG00000005655	0.054839883	-4.188630685	SMC2	Down
ENSOCUG00000008247	0.255792562	-1.966953779	-	Down
ENSOCUG00000004412	0.364677861	-1.455305477	AP1G1	Down
ENSOCUG00000022951	0.109887492	-3.18590092	-	Down
ENSOCUG00000025674	0.203631954	-2.295964126	SPTY2D1	Down
ENSOCUG00000008212	0.119820953	-3.061047883	DNTTIP2	Down
ENSOCUG00000023957	0.049319461	-4.34169916	-	Down
ENSOCUG00000012358	0.138876021	-2.848130575	ATP11B	Down
ENSOCUG00000015669	0.101467738	-3.300906999	ARAP2	Down
ENSOCUG00000003026	0.292473165	-1.773623833	DHX15	Down
ENSOCUG00000003843	0.238749822	-2.066428436	DDX10	Down
ENSOCUG00000012730	0.124782957	-3.002507187	TAX1BP1	Down
ENSOCUG00000014647	0.100528724	-3.31432031	USP15	Down
ENSOCUG00000003686	0.259511872	-1.946127554	USP8	Down
ENSOCUG00000004403	0.134946475	-2.889540806	-	Down
ENSOCUG00000015368	0.410815252	-1.283438351	FAM83A	Down
ENSOCUG00000017716	0.171045983	-2.547543873	CPS1	Down
ENSOCUG00000013150	0.405441973	-1.302432645	DSP	Down
ENSOCUG00000005226	0.345086033	-1.53497201	ZNF143	Down
ENSOCUG00000008071	0.410224888	-1.285513074	-	Down
ENSOCUG00000004848	0.107834234	-3.213112829	ROCK1	Down
ENSOCUG00000014069	0.103827307	-3.267742172	ZNF507	Down
ENSOCUG00000001751	0.367051384	-1.445946054	RND3	Down
ENSOCUG00000002098	0.400271463	-1.320949331	FCHSD1	Down
ENSOCUG00000008962	0.355503674	-1.492063627	LNPK	Down
ENSOCUG00000011423	0.122274454	-3.031805077	LMBRD2	Down
ENSOCUG00000008720	0.193060685	-2.372873691	SEC62	Down
ENSOCUG00000006220	0.205728001	-2.281189924	CLCN3	Down
ENSOCUG00000000107	0.397028452	-1.332685698	MPPE1	Down
ENSOCUG00000016427	0.162193279	-2.624214056	TRIM33	Down
ENSOCUG00000009784	0.124280276	-3.00833075	FOXN2	Down
ENSOCUG00000010614	0.081837766	-3.611089428	-	Down
ENSOCUG00000003064	0.233378295	-2.099257701	NUP153	Down
ENSOCUG00000004446	0.400188983	-1.321246643	VPS37B	Down
ENSOCUG00000000174	0.206883433	-2.273109975	EXOC6	Down
ENSOCUG00000000218	0.161965277	-2.626243541	OTUD6B	Down
ENSOCUG00000001798	0.152145351	-2.716477845	ACAP2	Down
ENSOCUG00000014398	0.40271878	-1.312155343	RAP2B	Down
ENSOCUG00000006230	0.119156301	-3.06907285	UPF2	Down
ENSOCUG00000038504	0.16972761	-2.558706825	C1orf210	Down
ENSOCUG00000009041	0.186568261	-2.422224519	BOD1L1	Down
ENSOCUG00000011903	0.210710564	-2.246665449	ZC3H15	Down
ENSOCUG00000017272	0.101237	-3.304191433	COBLL1	Down

ENSOCUG00000012546	0.401276764	-1.317330477	ZNF462	Down
ENSOCUG00000029164	0.287854471	-1.796588475	-	Down
ENSOCUG00000011084	0.185006822	-2.434349627	PPIG	Down
ENSOCUG00000003943	0.395356089	-1.338775452	CLCF1	Down
ENSOCUG00000010663	0.07575205	-3.722571253	MAPK10	Down
ENSOCUG00000017567	0.397309708	-1.331664049	-	Down
ENSOCUG00000006049	0.308864627	-1.694953442	VCPKMT	Down
ENSOCUG00000003125	0.08655567	-3.530227862	TRPM7	Down
ENSOCUG00000015953	0.18486989	-2.435417827	STK17A	Down
ENSOCUG00000014034	0.334191997	-1.581250912	C3orf38	Down
ENSOCUG00000008428	0.06935911	-3.849770806	-	Down
ENSOCUG00000013799	0.133152527	-2.908848287	UACA	Down
ENSOCUG00000011514	0.151372607	-2.723823939	MFAP3L	Down
ENSOCUG00000016769	0.347910289	-1.523212748	RIPOR3	Down
ENSOCUG00000002693	0	-Inf	CATSPERB	Down
ENSOCUG00000023926	0.149619297	-2.740631841	ZNF711	Down
ENSOCUG00000005597	0.354037046	-1.498027765	FMN1	Down
ENSOCUG00000006180	0.39923955	-1.324673449	LPCAT4	Down
ENSOCUG00000007324	0.22410461	-2.157755769	ADGRG2	Down
ENSOCUG00000001742	0.093919417	-3.412432742	CASP8AP2	Down
ENSOCUG00000000617	0.386381063	-1.371903707	UTP15	Down
ENSOCUG00000036679	0.016304065	-5.938624491	-	Down
ENSOCUG00000026148	0.07455514	-3.745548372	-	Down
ENSOCUG00000017331	0.118865957	-3.07259251	NAA15	Down
ENSOCUG00000038349	0.09721326	-3.362703083	-	Down
ENSOCUG00000001219	0.14398508	-2.796008768	RASA2	Down
ENSOCUG00000005712	0.282400561	-1.824185143	-	Down
ENSOCUG00000000918	0.180768399	-2.4677856	TLX3	Down
ENSOCUG00000001821	0.148987841	-2.7467335	-	Down
ENSOCUG00000014221	0.401209141	-1.317573617	-	Down
ENSOCUG00000011764	0.348767758	-1.519661421	LPP	Down
ENSOCUG00000015748	0.410232424	-1.285486569	NPEPPS	Down
ENSOCUG00000017819	0.155758787	-2.682614544	USP34	Down
ENSOCUG00000010712	0.319900098	-1.64430666	MFAP1	Down
ENSOCUG00000030530	0.228165654	-2.131846456	-	Down
ENSOCUG00000000144	0.086621305	-3.529134277	-	Down
ENSOCUG00000008982	0.116699233	-3.099133017	BLZF1	Down
ENSOCUG00000007823	0.163099896	-2.61617223	SGPP2	Down
ENSOCUG00000005376	0.394694494	-1.341191703	EIF3J	Down
ENSOCUG00000001076	0.184476117	-2.438494046	SOCS6	Down
ENSOCUG00000029102	0.117194848	-3.093018944	ATP6	Down
ENSOCUG00000005880	0.132848889	-2.912141928	PDS5A	Down
ENSOCUG00000017858	0.37472085	-1.416111841	-	Down
ENSOCUG00000010714	0.166522754	-2.58620877	SENP6	Down

ENSOCUG00000001300	0.357427749	-1.484276452	CCSER1	Down
ENSOCUG00000002784	0.388739861	-1.363123045	GTF2E1	Down
ENSOCUG00000010396	0.176230156	-2.504467281	SSB	Down
ENSOCUG00000001834	0.353235006	-1.501299773	ODF2L	Down
ENSOCUG00000026795	0.399913396	-1.322240488	-	Down
ENSOCUG00000015094	0.210984764	-2.244789274	-	Down
ENSOCUG00000004346	0.325507953	-1.619235302	RBBP6	Down
ENSOCUG00000014821	0.398648238	-1.326811801	CSNK1G3	Down
ENSOCUG00000027050	0.246691739	-2.019218688	LYPD3	Down
ENSOCUG00000016521	0.32991902	-1.599816142	RRAGC	Down
ENSOCUG00000003134	0.151223346	-2.725247209	MIER3	Down
ENSOCUG00000000924	0.381690803	-1.389523669	PIGW	Down
ENSOCUG00000015484	0.382559841	-1.386242658	CRNKL1	Down
ENSOCUG00000012261	0.183433902	-2.446667793	TPR	Down
ENSOCUG00000001418	0.405913167	-1.300756956	PSMD14	Down
ENSOCUG00000002083	0.344730162	-1.536460564	TMC7	Down
ENSOCUG00000016474	0.215056013	-2.217215627	KIAA2026	Down
ENSOCUG00000004852	0.277526307	-1.849303565	MALL	Down
ENSOCUG00000009266	0.112359223	-3.153809539	CAND2	Down
ENSOCUG00000016919	0.149616618	-2.740657672	CEP83	Down
ENSOCUG00000024369	0.370404885	-1.43282497	-	Down
ENSOCUG00000001082	0.413795463	-1.273010267	CLIP4	Down
ENSOCUG00000017086	0.23614894	-2.082231035	ZMYM2	Down
ENSOCUG00000015413	0.283073685	-1.820750456	PDCD10	Down
ENSOCUG00000008605	0.414607118	-1.270183209	PTPN1	Down
ENSOCUG00000001957	0.338127583	-1.564360386	-	Down
ENSOCUG00000015510	0.412979838	-1.275856747	C2CD2	Down
ENSOCUG00000024909	0.223639919	-2.160750367	-	Down
ENSOCUG00000017406	0.146089554	-2.775075073	-	Down
ENSOCUG00000029583	0.226370522	-2.143241995	-	Down
ENSOCUG00000004691	0.429649329	-1.218768453	PROM2	Down
ENSOCUG00000000593	0.401375304	-1.316976241	ZFYVE26	Down
ENSOCUG00000005465	0.394388041	-1.342312289	NOCT	Down
ENSOCUG00000021310	0.239928041	-2.059326318	-	Down
ENSOCUG00000015651	0.408151742	-1.292822481	ATG3	Down
ENSOCUG00000012200	0.223268081	-2.163151079	RAB5A	Down
ENSOCUG00000030742	0.41645801	-1.263757053	PAFAH1B1	Down
ENSOCUG00000006685	0.200049058	-2.321574257	GABPA	Down
ENSOCUG00000017800	0.411750819	-1.280156575	-	Down
ENSOCUG00000012712	0.134966807	-2.889323447	EPC2	Down
ENSOCUG00000002780	0.379534744	-1.397696135	PANK1	Down
ENSOCUG00000012136	0.125360873	-2.995840965	TBK1	Down
ENSOCUG00000013721	0.398136559	-1.32866474	CRY1	Down
ENSOCUG00000000425	0.234082395	-2.094911657	PRPF4B	Down

ENSOCUG00000009451	0.194612338	-2.36132492	PIK3C3	Down
ENSOCUG00000014123	0.385005052	-1.377050717	PPP1R2	Down
ENSOCUG00000029025	0.40929008	-1.288804396	CSTA	Down
ENSOCUG00000027940	0.129592198	-2.947949232	HSPA4L	Down
ENSOCUG00000011524	0.270381551	-1.886931381	USP38	Down
ENSOCUG00000000083	0.166665784	-2.58497014	HTRA4	Down
ENSOCUG00000016464	0.186323003	-2.424122301	-	Down
ENSOCUG00000029101	0.108286743	-3.207071462	ATP8	Down
ENSOCUG00000004646	0.195493084	-2.354810522	RASA1	Down
ENSOCUG00000025362	0.261836834	-1.933260032	RND1	Down
ENSOCUG00000024444	0.369236328	-1.437383593	CRHR2	Down
ENSOCUG00000016503	0.238735057	-2.066517661	WAPL	Down
ENSOCUG00000030482	0.376413884	-1.409608254	-	Down
ENSOCUG00000016181	0.111588323	-3.163742034	-	Down
ENSOCUG00000004434	0.124658476	-3.00394712	CHD1	Down
ENSOCUG00000022150	0.054807211	-4.189490477	-	Down
ENSOCUG00000004454	0.16024617	-2.641638218	DHX29	Down
ENSOCUG00000032317	0	-Inf	-	Down
ENSOCUG00000005642	0.235158262	-2.088296072	MTPN	Down
ENSOCUG00000012972	0.410539161	-1.28440825	CRYAB	Down
ENSOCUG00000016074	0.364334047	-1.456666272	TSC22D2	Down
ENSOCUG00000013765	0.422759058	-1.242092427	SNX9	Down
ENSOCUG00000006455	0.133622876	-2.90376108	COPS2	Down
ENSOCUG00000030393	0.295000675	-1.76120984	-	Down
ENSOCUG00000008165	0.418529813	-1.256597702	MTFR1	Down
ENSOCUG00000032273	0.420407398	-1.250140038	RAP1B	Down
ENSOCUG00000009069	0.419947984	-1.251717451	UBAP1	Down
ENSOCUG00000007193	0.21731179	-2.202161648	AP4E1	Down
ENSOCUG00000029113	0.416767922	-1.262683855	ND6	Down
ENSOCUG00000005896	0.132415553	-2.916855507	INPP4B	Down
ENSOCUG00000010777	0.167424236	-2.578419707	DOP1A	Down
ENSOCUG00000000756	0.139884791	-2.837688982	-	Down
ENSOCUG00000003516	0.298617333	-1.743630188	-	Down
ENSOCUG00000000806	0.098124598	-3.349241351	TTC14	Down
ENSOCUG00000027653	0.059936538	-4.060420427	-	Down
ENSOCUG00000002468	0.05429519	-4.203031797	KIF20B	Down
ENSOCUG00000011272	0.405329889	-1.30283153	ARIH1	Down
ENSOCUG00000008812	0.045550069	-4.456402937	POF1B	Down
ENSOCUG00000027691	0.16160403	-2.629464918	-	Down
ENSOCUG00000029108	0.147268771	-2.763476561	ND4	Down
ENSOCUG00000029143	0.139815987	-2.838398767	-	Down
ENSOCUG00000029099	0.143936957	-2.796491028	COX2	Down
ENSOCUG00000006420	0.411391148	-1.281417344	SMCR8	Down
ENSOCUG00000001857	0.380130435	-1.395433557	ZBTB10	Down

ENSOCUG00000017815	0.130916758	-2.933278316	CWH43	Down
ENSOCUG00000011638	0.415333032	-1.267659478	RBM6	Down
ENSOCUG00000003976	0.409077816	-1.289552793	GOLGA1	Down
ENSOCUG00000004886	0.418785562	-1.25571639	AKIRIN2	Down
ENSOCUG00000006498	0.042358381	-4.561208756	-	Down
ENSOCUG00000024540	0.433382341	-1.206287726	-	Down
ENSOCUG00000001804	0.264305054	-1.919724084	RPAP2	Down
ENSOCUG00000017670	0.161350184	-2.631732869	PTBP3	Down
ENSOCUG00000005742	0.349434942	-1.516904218	KCTD14	Down
ENSOCUG00000012549	0.418670701	-1.256112134	MEX3C	Down
ENSOCUG00000000392	0.364825953	-1.454719731	ARMC1	Down
ENSOCUG00000013277	0.140623115	-2.830094339	CCAR1	Down
ENSOCUG00000017263	0.144474657	-2.791111651	RLF	Down
ENSOCUG00000010527	0.180884104	-2.466862466	OSBPL8	Down
ENSOCUG00000012031	0.426124106	-1.230654428	PLEK2	Down
ENSOCUG00000016120	0.331905363	-1.591156152	-	Down
ENSOCUG00000014750	0.410387385	-1.284941711	TENT4B	Down
ENSOCUG00000034911	0.326799153	-1.613523849	-	Down
ENSOCUG00000033788	0.417300935	-1.260839941	-	Down
ENSOCUG00000021080	0.418161191	-1.25786892	MBD2	Down
ENSOCUG00000029115	0.143531041	-2.800565313	CYTB	Down
ENSOCUG00000016688	0.135210518	-2.886720711	MTM1	Down
ENSOCUG00000013429	0.401697906	-1.315817154	NHSL1	Down
ENSOCUG00000016003	0.297167979	-1.750649427	KCNK1	Down
ENSOCUG00000000127	0.258105932	-1.953964794	-	Down
ENSOCUG00000015028	0.075826863	-3.721147155	SLC6A14	Down
ENSOCUG00000024835	0.357377228	-1.484480386	ALG11	Down
ENSOCUG00000016958	0.292160912	-1.775164921	PCMTD1	Down
ENSOCUG00000000334	0.392525153	-1.34914299	TOX4	Down
ENSOCUG00000009618	0.388525421	-1.3639191	SLAIN2	Down
ENSOCUG00000001165	0.093367518	-3.420935463	-	Down
ENSOCUG00000009433	0.273242184	-1.871747867	LPIN2	Down
ENSOCUG00000010182	0.273576533	-1.86998361	MLH3	Down
ENSOCUG00000004276	0.39984248	-1.322496341	MTERF3	Down
ENSOCUG00000000547	0.09263514	-3.432296623	-	Down
ENSOCUG00000013209	0.261764801	-1.933656979	PTPRZ1	Down
ENSOCUG00000011978	0.083475239	-3.58250787	FAM124B	Down
ENSOCUG00000012511	0.135701536	-2.881491048	PMS1	Down
ENSOCUG00000012582	0.414717912	-1.269797737	RAB21	Down
ENSOCUG00000009484	0.072019677	-3.795465054	ECT2	Down
ENSOCUG00000002839	0.217319568	-2.202110011	-	Down
ENSOCUG00000003294	0.310878885	-1.685575464	WDR26	Down
ENSOCUG00000031226	0.406135554	-1.299966765	-	Down
ENSOCUG00000004184	0.427813404	-1.22494641	SLC39A8	Down

ENSOCUG00000022299	0.06948348	-3.847186174	PI15	Down
ENSOCUG00000008154	0.206257903	-2.277478697	TMF1	Down
ENSOCUG00000003338	0.418296819	-1.257401069	RNF111	Down
ENSOCUG00000000876	0.217773846	-2.199097396	INTS6	Down
ENSOCUG00000003201	0.428201888	-1.223636937	BCL2L1	Down
ENSOCUG00000010932	0.20349242	-2.29695304	GPBP1	Down
ENSOCUG00000009173	0.159241905	-2.650708062	RBM34	Down
ENSOCUG00000004957	0.146208579	-2.773900126	GALNT3	Down
ENSOCUG00000000038	0.093466226	-3.419411054	CREBRF	Down
ENSOCUG00000034094	0.321010619	-1.639307074	EIF4EBP3	Down
ENSOCUG00000012531	0.253116218	-1.982128147	NEU3	Down
ENSOCUG00000008053	0.176965091	-2.498463298	NOL8	Down
ENSOCUG00000006467	0.407029374	-1.296795181	NAA50	Down
ENSOCUG00000004653	0.222830385	-2.165982122	METAP2	Down
ENSOCUG00000012593	0.156575059	-2.675073673	BAZ1A	Down
ENSOCUG00000024612	0.42524135	-1.233646205	FAM102A	Down
ENSOCUG00000017116	0	-Inf	-	Down
ENSOCUG00000039573	0.065080763	-3.941625032	GJB7	Down
ENSOCUG00000015836	0.393038934	-1.347255862	MPZL2	Down
ENSOCUG00000021847	0.136651355	-2.871428335	-	Down
ENSOCUG00000012292	0.222508668	-2.168066557	CASD1	Down
ENSOCUG00000003437	0	-Inf	-	Down
ENSOCUG00000010650	0.148393534	-2.752499865	KTN1	Down
ENSOCUG00000022047	0.154127865	-2.697800383	-	Down
ENSOCUG00000014223	0.437949961	-1.191162055	UVRAG	Down
ENSOCUG00000011923	0.304115495	-1.71730877	RSF1	Down
ENSOCUG00000025190	0.144874226	-2.787127145	LRRC58	Down
ENSOCUG00000008998	0.291484181	-1.778510505	KCNJ14	Down
ENSOCUG00000003095	0.088367893	-3.500333906	CIP2A	Down
ENSOCUG00000033301	0.306962107	-1.70386752	-	Down
ENSOCUG00000024670	0.165819499	-2.592314432	ZNF770	Down
ENSOCUG00000039314	0.314767717	-1.667640512	-	Down
ENSOCUG00000002099	0.415171486	-1.268220731	MADD	Down
ENSOCUG00000001698	0.355627668	-1.491560524	MAP3K7	Down
ENSOCUG00000032320	0.412644051	-1.277030252	-	Down
ENSOCUG00000016381	0.194804789	-2.359898953	BROX	Down
ENSOCUG00000013287	0.204864304	-2.28725947	-	Down
ENSOCUG00000005077	0.241463038	-2.05012573	TOPORS	Down
ENSOCUG00000005701	0.377136988	-1.406839442	-	Down
ENSOCUG00000000819	0.31666338	-1.658978058	ABHD17B	Down
ENSOCUG00000010076	0.425599892	-1.23243031	ABI1	Down
ENSOCUG00000001180	0.119627673	-3.063376932	DHX36	Down
ENSOCUG00000011167	0.088262534	-3.502055026	-	Down
ENSOCUG00000011712	0.446307179	-1.163891081	AFAP1L2	Down

ENSOCUG00000011142	0.163585629	-2.611882083	SMC3	Down
ENSOCUG00000015417	0.387796298	-1.366629064	CSTF3	Down
ENSOCUG00000009401	0.38437212	-1.379424401	-	Down
ENSOCUG00000014007	0.180130411	-2.472886326	HEATR1	Down
ENSOCUG00000001420	0.401994205	-1.314753391	SYNJ1	Down
ENSOCUG00000021705	0.415054813	-1.268626221	-	Down
ENSOCUG00000029290	0.199554162	-2.325147726	-	Down
ENSOCUG00000007579	0.133919262	-2.900564614	ATRX	Down
ENSOCUG00000006390	0.276689571	-1.853659827	AFTPH	Down
ENSOCUG00000012366	0.286134543	-1.80523442	SLC25A46	Down
ENSOCUG00000004742	0.178027882	-2.489824885	USP25	Down
ENSOCUG00000012189	0.155570463	-2.684359927	MAPK6	Down
ENSOCUG00000012819	0.153209079	-2.706426302	CHM	Down
ENSOCUG00000000543	0.306950212	-1.703923427	RNF144B	Down
ENSOCUG00000015134	0.369943701	-1.434622362	ARMC8	Down
ENSOCUG00000016667	0.24843274	-2.009072779	LATS1	Down
ENSOCUG00000015620	0.161864259	-2.627143632	APC	Down
ENSOCUG00000025456	0.440183189	-1.183824047	MAP7	Down
ENSOCUG00000007873	0.414224081	-1.271516668	IKBKG	Down
ENSOCUG00000016701	0.205345688	-2.283873444	PPP1R12A	Down
ENSOCUG00000013051	0.445505603	-1.166484518	-	Down
ENSOCUG00000012814	0.296402791	-1.754369062	RO60	Down
ENSOCUG00000025603	0.180359026	-2.471056468	GPA33	Down
ENSOCUG00000015961	0.432437368	-1.2094369	-	Down
ENSOCUG00000005541	0.119956758	-3.059413655	PIK3C2A	Down
ENSOCUG00000014093	0.042737607	-4.548350047	GNRH1	Down
ENSOCUG00000001390	0.234542874	-2.092076423	GOLGA4	Down
ENSOCUG00000001788	0.103964533	-3.265836649	RASGRP1	Down
ENSOCUG00000007858	0.413586207	-1.273740022	-	Down
ENSOCUG00000000117	0.103927785	-3.266346692	TM4SF20	Down
ENSOCUG00000008221	0.210561107	-2.247689114	TOP1	Down
ENSOCUG00000012547	0.173831206	-2.524240995	USP54	Down
ENSOCUG00000002827	0.264322876	-1.919626804	IDE	Down
ENSOCUG00000004290	0.227307041	-2.137285721	CEP290	Down
ENSOCUG00000006536	0.398662937	-1.326758606	-	Down
ENSOCUG00000001513	0.216131358	-2.210019693	VPS26A	Down
ENSOCUG00000003771	0.19626419	-2.349131133	STAM	Down
ENSOCUG00000006880	0.194969188	-2.358681951	PPP4R3B	Down
ENSOCUG00000017134	0.353816085	-1.498928458	SMAP1	Down
ENSOCUG00000008239	0.248598077	-2.00811296	CAB39L	Down
ENSOCUG00000008195	0.337383754	-1.567537591	ZNF207	Down
ENSOCUG00000037295	0.075806725	-3.721530349	-	Down
ENSOCUG00000003155	0.415476992	-1.267159509	PLAU	Down
ENSOCUG00000017824	0.397637602	-1.330473905	CCDC59	Down

ENSOCUG00000003631	0.213546472	-2.227378034	NOP58	Down
ENSOCUG00000006933	0.29724318	-1.750284385	-	Down
ENSOCUG00000000925	0.307665683	-1.70056456	GGNBP2	Down
ENSOCUG00000027756	0.355001524	-1.494102877	-	Down
ENSOCUG00000001319	0.271430345	-1.881346077	SREK1	Down
ENSOCUG00000017852	0.249851947	-2.000854632	AQP3	Down
ENSOCUG00000005947	0.065240054	-3.938098207	SCEL	Down
ENSOCUG00000007241	0.443636247	-1.172550849	IPPK	Down
ENSOCUG00000029569	0.442686179	-1.175643763	KRT17	Down
ENSOCUG00000016584	0.401273097	-1.317343661	PLEKHA5	Down
ENSOCUG00000017654	0.19890494	-2.329848985	KATNBL1	Down
ENSOCUG00000004479	0.361830256	-1.466615044	SLC38A9	Down
ENSOCUG00000004040	0.418073753	-1.258170621	UTP25	Down
ENSOCUG00000027088	0.270606527	-1.885731459	PPP2R2A	Down
ENSOCUG00000002505	0.429326273	-1.219853633	TXNL1	Down
ENSOCUG00000017283	0.12321796	-3.020715542	SMARCAD1	Down
ENSOCUG00000015438	0.165413099	-2.595854606	MIER1	Down
ENSOCUG00000014442	0.093935976	-3.412178396	H1-4	Down
ENSOCUG00000006944	0.130647996	-2.936243098	SPOPL	Down
ENSOCUG00000000706	0.393652141	-1.345006771	FHOD3	Down
ENSOCUG00000011691	0.176242243	-2.504368337	STK17B	Down
ENSOCUG00000007821	0.43997091	-1.184519956	AVPI1	Down
ENSOCUG00000026872	0.154960447	-2.690028071	-	Down
ENSOCUG00000004950	0.41003308	-1.286187789	-	Down
ENSOCUG00000013399	0	-Inf	-	Down
ENSOCUG00000015493	0.271055482	-1.883339907	ANXA1	Down
ENSOCUG00000015656	0.264877865	-1.916600809	-	Down
ENSOCUG00000015410	0.095872571	-3.382738064	NDC80	Down
ENSOCUG00000001672	0.131967378	-2.921746752	-	Down
ENSOCUG00000003950	0.430339174	-1.216453918	EPG5	Down
ENSOCUG00000036082	0.268738317	-1.895726056	-	Down
ENSOCUG00000021196	0.206006463	-2.279238498	EPB41L3	Down
ENSOCUG00000000916	0.330045861	-1.599261591	PIP4K2C	Down
ENSOCUG00000022792	0.236302773	-2.081291538	-	Down
ENSOCUG00000017508	0.432088714	-1.210600545	ZNF622	Down
ENSOCUG00000003327	0.194874701	-2.359381286	SLTM	Down
ENSOCUG00000017919	0.122556868	-3.028476759	CEP135	Down
ENSOCUG00000004189	0.32323077	-1.629363554	-	Down
ENSOCUG00000009409	0.436149845	-1.197104219	TRAPPC13	Down
ENSOCUG00000003855	0.43459675	-1.202250708	-	Down
ENSOCUG00000031910	0.384253475	-1.379869787	SREK1IP1	Down
ENSOCUG00000016469	0.180331387	-2.471277574	MLANA	Down
ENSOCUG00000004463	0.181968632	-2.45823832	MTREX	Down
ENSOCUG00000015730	0.170310063	-2.553764411	TAOK1	Down

ENSOCUG00000003770	0.426538873	-1.229250865	MED6	Down
ENSOCUG00000029263	0.129303288	-2.951169138	RARRES1	Down
ENSOCUG00000013606	0.158351811	-2.65879473	JAKMIP2	Down
ENSOCUG00000000656	0.317014614	-1.657378746	SLC39A2	Down
ENSOCUG00000027628	0.282026984	-1.826094888	ARL14EP	Down
ENSOCUG00000027203	0	-Inf	AZGP1	Down
ENSOCUG00000007930	0.428422925	-1.222892415	ATF6	Down
ENSOCUG00000015771	0.337009374	-1.569139372	SQLE	Down
ENSOCUG00000023173	0.369744886	-1.435397901	LIPT1	Down
ENSOCUG00000014733	0.043895558	-4.509781252	-	Down
ENSOCUG00000001112	0.07512448	-3.734573088	PCM1	Down
ENSOCUG00000034571	0.098257368	-3.347290589	-	Down
ENSOCUG00000021775	0.05174651	-4.272394616	MMP10	Down
ENSOCUG00000007109	0.399116919	-1.325116657	DIAPH2	Down
ENSOCUG00000008250	0.442153114	-1.177382044	SAR1B	Down
ENSOCUG00000001052	0.270967969	-1.883805775	ARID4A	Down
ENSOCUG00000012752	0.355225042	-1.493194804	SIRPB2	Down
ENSOCUG00000007535	0.428202417	-1.223635155	-	Down
ENSOCUG00000011635	0.439600094	-1.1857364	ACAD9	Down
ENSOCUG00000016726	0.277505319	-1.849412673	TRIP12	Down
ENSOCUG00000010435	0.229825098	-2.121391737	SUSD1	Down
ENSOCUG00000013023	0.316240336	-1.660906701	-	Down
ENSOCUG00000014452	0.264597332	-1.918129582	SERPINB2	Down
ENSOCUG00000025998	0.100421209	-3.315864099	ANKRD12	Down
ENSOCUG00000024804	0.421133585	-1.247650162	BTBD19	Down
ENSOCUG00000013811	0.429718392	-1.218536568	SCYL3	Down
ENSOCUG00000000717	0.158811781	-2.65461016	TOP2B	Down
ENSOCUG00000007836	0.207598219	-2.268134028	TAB3	Down
ENSOCUG00000003325	0.312835348	-1.676524562	SOWAHC	Down
ENSOCUG00000013306	0.412907895	-1.276108091	ARID2	Down
ENSOCUG00000015213	0.173055077	-2.530696824	-	Down
ENSOCUG00000029356	0.181733015	-2.460107561	MYSM1	Down
ENSOCUG00000015565	0.118200234	-3.080695202	KCNJ5	Down
ENSOCUG00000014348	0.157781842	-2.663996914	SHOC2	Down
ENSOCUG00000010433	0.205174455	-2.285076976	BTAF1	Down
ENSOCUG00000005756	0.388448535	-1.364204625	TMEM41B	Down
ENSOCUG00000004826	0.242184305	-2.045822724	SLC40A1	Down
ENSOCUG00000001717	0.215398534	-2.214919663	SKAP1	Down
ENSOCUG00000002497	0.168621488	-2.568139699	CNOT6L	Down
ENSOCUG00000015462	0.423465583	-1.239683374	PRRG1	Down
ENSOCUG00000021069	0.064930353	-3.94496314	-	Down
ENSOCUG00000007066	0.424086083	-1.237570954	USP6NL	Down
ENSOCUG00000024410	0	-Inf	-	Down
ENSOCUG00000015520	0.131267219	-2.929421411	PHF3	Down

ENSOCUG00000006889	0.200360569	-2.319329481	PNPT1	Down
ENSOCUG00000026115	0.246182858	-2.022197786	-	Down
ENSOCUG00000002107	0.375452503	-1.413297686	CALM2	Down
ENSOCUG00000026311	0.229516964	-2.123327306	-	Down
ENSOCUG00000004591	0.116962724	-3.09587928	ZFC3H1	Down
ENSOCUG00000012328	0.177759981	-2.491997531	RNF128	Down
ENSOCUG00000000220	0.184851531	-2.435561103	MBNL2	Down
ENSOCUG00000010475	0.165767258	-2.592769014	UTP20	Down
ENSOCUG00000005265	0.132330088	-2.91778697	NRIP1	Down
ENSOCUG00000029298	0.353812253	-1.498944082	CIR1	Down
ENSOCUG00000006100	0.440261103	-1.183568707	PNO1	Down
ENSOCUG00000003618	0.166303927	-2.588105856	MACC1	Down
ENSOCUG00000004485	0.252495927	-1.985667977	E2F8	Down
ENSOCUG00000034611	0.417351899	-1.260663759	CEP76	Down
ENSOCUG00000017384	0.182148271	-2.456814796	NEMF	Down
ENSOCUG00000002143	0.143940804	-2.796452477	VPS54	Down
ENSOCUG00000000197	0.437540745	-1.192510725	HK2	Down
ENSOCUG00000005932	0.223851619	-2.159385345	CCDC93	Down
ENSOCUG00000015832	0.129724386	-2.946478386	OPA1	Down
ENSOCUG00000031862	0.253218932	-1.981542825	ZNF146	Down
ENSOCUG00000006781	0.183703307	-2.444550498	KPNA3	Down
ENSOCUG00000004899	0.258803418	-1.950071423	PIK3CB	Down
ENSOCUG00000009919	0.329832068	-1.600196421	SIRT1	Down
ENSOCUG00000014516	0.049930242	-4.323942305	CCDC68	Down
ENSOCUG00000023933	0.1181864	-3.080864063	CKAP2	Down
ENSOCUG00000004211	0.428339104	-1.223174704	TMEM102	Down
ENSOCUG00000002730	0.386123192	-1.372866884	LRRC1	Down
ENSOCUG00000007835	0.241402205	-2.050489238	SCFD1	Down
ENSOCUG00000009725	0.116833563	-3.097473311	AWAT1	Down
ENSOCUG00000035839	0.391941875	-1.351288377	-	Down
ENSOCUG00000003106	0.330333726	-1.598003824	-	Down
ENSOCUG00000015242	0.441352434	-1.17999694	PIP5K1A	Down
ENSOCUG00000004898	0.301830957	-1.728187311	CDC6	Down
ENSOCUG00000006014	0.156693189	-2.673985628	PDE6H	Down
ENSOCUG00000010631	0.139264008	-2.844105648	PIK3CA	Down
ENSOCUG00000015886	0.318443894	-1.650888885	YTHDC1	Down
ENSOCUG00000010261	0.370189991	-1.433662205	EHF	Down
ENSOCUG00000029112	0.439693131	-1.1854311	ND5	Down
ENSOCUG00000011645	0.172155591	-2.538215058	AHI1	Down
ENSOCUG00000013326	0.284659473	-1.812690985	EIF4G2	Down
ENSOCUG00000035451	0.190985917	-2.388461837	-	Down
ENSOCUG00000014338	0.191927941	-2.381363342	ABCA5	Down
ENSOCUG00000035488	0.420313372	-1.25046274	-	Down
ENSOCUG00000017849	0.198106932	-2.335648731	GCC2	Down

ENSOCUG00000014099	0.192442376	-2.377501577	KCTD9	Down
ENSOCUG00000011383	0.282705542	-1.822627932	FAM241A	Down
ENSOCUG00000012039	0.262470716	-1.929771626	EPRS1	Down
ENSOCUG00000013239	0.216267721	-2.209109739	-	Down
ENSOCUG00000027318	0.429949526	-1.217760791	WDR53	Down
ENSOCUG00000007643	0.142685724	-2.809087095	NIPAL1	Down
ENSOCUG00000000681	0.307883195	-1.69954497	TXLNG	Down
ENSOCUG00000011554	0.158258167	-2.659648146	-	Down
ENSOCUG00000024606	0.200533861	-2.31808223	MYO6	Down
ENSOCUG00000004967	0.126339998	-2.984616635	ZNF654	Down
ENSOCUG00000011224	0.415503777	-1.267066503	RNF24	Down
ENSOCUG00000015532	0.421155009	-1.247576771	CACHD1	Down
ENSOCUG00000011027	0.239741695	-2.060447254	CHIT1	Down
ENSOCUG00000000587	0.156779461	-2.67319152	DNAJC13	Down
ENSOCUG00000014307	0.447792611	-1.159097371	DUOX1	Down
ENSOCUG00000021166	0.153504517	-2.703646986	-	Down
ENSOCUG00000005257	0.318448273	-1.650869045	DNAJC21	Down
ENSOCUG00000013416	0	-Inf	-	Down
ENSOCUG00000004049	0.44553745	-1.166381391	TARDBP	Down
ENSOCUG00000001142	0.227064577	-2.138825435	SNX13	Down
ENSOCUG00000006693	0.29183277	-1.776786203	INTS2	Down
ENSOCUG00000002926	0.113525044	-3.138917505	DLGAP5	Down
ENSOCUG00000004753	0.452952153	-1.142569433	KANK1	Down
ENSOCUG00000013117	0.184768174	-2.436211821	-	Down
ENSOCUG00000009172	0.44120545	-1.180477482	IRX2	Down
ENSOCUG00000016277	0.020106861	-5.636168316	EREG	Down
ENSOCUG00000002494	0.374986071	-1.415091087	FBXO33	Down
ENSOCUG00000007124	0.081930528	-3.609455076	TTK	Down
ENSOCUG00000002225	0.349275908	-1.517560963	-	Down
ENSOCUG00000034985	0.418793083	-1.25569048	FAM133B	Down
ENSOCUG00000013490	0.443198872	-1.173973886	RRS1	Down
ENSOCUG00000008387	0.446412284	-1.163551367	VSIG10	Down
ENSOCUG00000008319	0	-Inf	-	Down
ENSOCUG00000032016	0.338099461	-1.564480379	-	Down
ENSOCUG00000010253	0.43613239	-1.197161957	TMEM209	Down
ENSOCUG00000004462	0.283946125	-1.816310869	PCF11	Down
ENSOCUG00000009036	0.084229684	-3.569527428	C16orf86	Down
ENSOCUG00000002767	0.181912181	-2.458685941	CEP162	Down
ENSOCUG00000005389	0.433258647	-1.206699552	MTF1	Down
ENSOCUG00000004008	0.29120829	-1.77987667	CNDP1	Down
ENSOCUG00000009414	0.248951244	-2.006064871	C17orf64	Down
ENSOCUG00000012900	0.417764518	-1.259238128	RORA	Down
ENSOCUG00000015739	0.405126289	-1.30355639	ABHD13	Down
ENSOCUG00000021983	0.173191464	-2.529560267	-	Down

ENSOCUG00000007230	0.198404431	-2.333483846	CEBPZ	Down
ENSOCUG00000010609	0.1896311	-2.398732507	DUSP16	Down
ENSOCUG00000014608	0.101507942	-3.300335479	FABP3	Down
ENSOCUG00000009241	0.427688302	-1.225368348	RAB11FIP2	Down
ENSOCUG00000001175	0.447366805	-1.160469884	HACD2	Down
ENSOCUG00000025278	0.449864713	-1.152436887	ARHGAP32	Down
ENSOCUG00000015354	0.233947253	-2.095744805	ITCH	Down
ENSOCUG00000010045	0.178668474	-2.484643001	ZNF638	Down
ENSOCUG00000002632	0.403846284	-1.308121829	KRT7	Down
ENSOCUG00000015252	0.336196726	-1.57262242	BBS12	Down
ENSOCUG00000016614	0.449148754	-1.154734763	NDEL1	Down
ENSOCUG00000023368	0.27910578	-1.841116092	LNX1	Down
ENSOCUG00000012942	0.195402835	-2.355476695	USP1	Down
ENSOCUG00000002536	0.176882356	-2.499137951	SUZ12	Down
ENSOCUG00000003284	0.418918794	-1.255257486	CLDN12	Down
ENSOCUG00000030969	0	-Inf	-	Down
ENSOCUG00000031899	0.39748325	-1.331034027	TAF13	Down
ENSOCUG00000008373	0.433310601	-1.206526564	WASL	Down
ENSOCUG00000024818	0.28792252	-1.796247458	-	Down
ENSOCUG00000002763	0.445553148	-1.16633056	KIF1B	Down
ENSOCUG00000010517	0.383538235	-1.382557687	SLC30A7	Down
ENSOCUG00000016507	0.176703078	-2.500600924	ATP2B1	Down
ENSOCUG00000001172	0.189173401	-2.402218844	CCDC138	Down
ENSOCUG00000013619	0.375177272	-1.414355662	WDR44	Down
ENSOCUG00000029691	0.162718762	-2.619547489	-	Down
ENSOCUG00000005836	0.461685998	-1.115016117	NRG1	Down
ENSOCUG00000028116	0.24834211	-2.009599184	OBI1	Down
ENSOCUG00000016143	0.459237938	-1.122686265	GALNT6	Down
ENSOCUG00000006705	0.287827271	-1.796724803	GPR156	Down
ENSOCUG00000003847	0.187038081	-2.418596061	-	Down
ENSOCUG00000003006	0.43546015	-1.199387396	LRP6	Down
ENSOCUG00000035705	0.206233334	-2.277650557	-	Down
ENSOCUG00000001097	0.316707117	-1.658778807	CGRRF1	Down
ENSOCUG00000039030	0	-Inf	-	Down
ENSOCUG00000006235	0.442694845	-1.17561552	PPP4R2	Down
ENSOCUG00000007297	0.455386872	-1.134835395	AFDN	Down
ENSOCUG00000027557	0.451917861	-1.145867519	-	Down
ENSOCUG00000006052	0.216387	-2.208314265	SOS2	Down
ENSOCUG00000004003	0.439095429	-1.187393578	MAPK1IP1L	Down
ENSOCUG00000024578	0.401682362	-1.315872981	C9orf85	Down
ENSOCUG00000004603	0	-Inf	-	Down
ENSOCUG00000010899	0.45478392	-1.136746851	-	Down
ENSOCUG00000026433	0.381362811	-1.390763931	-	Down
ENSOCUG00000004256	0.268502426	-1.896992974	WBP4	Down

ENSOCUG00000011619	0.285927204	-1.806280205	-	Down
ENSOCUG00000012805	0.25029943	-1.99827309	GCLC	Down
ENSOCUG00000015046	0.242424806	-2.044390765	LRPPRC	Down
ENSOCUG00000021934	0.436099547	-1.197270604	TRMT1	Down
ENSOCUG00000003708	0.103737145	-3.268995529	CFAP299	Down
ENSOCUG00000008783	0.3522194	-1.505453719	UBXN2A	Down
ENSOCUG00000004524	0.19170158	-2.383065865	AKAP9	Down
ENSOCUG00000010468	0.450783026	-1.149494901	CSTF2	Down
ENSOCUG00000025549	0.260746111	-1.939282358	-	Down
ENSOCUG00000004936	0.324866958	-1.622079077	NAV3	Down
ENSOCUG00000008197	0.042261568	-4.56450989	PCDH8	Down
ENSOCUG00000011450	0.28892724	-1.791221869	WDR75	Down
ENSOCUG00000010675	0.272378832	-1.876313505	ORC2	Down
ENSOCUG00000006050	0.165602016	-2.594207857	SGO1	Down
ENSOCUG00000001016	0.198547552	-2.332443522	-	Down
ENSOCUG00000029460	0.235971302	-2.083316681	PNLIPRP2	Down
ENSOCUG00000010660	0.251016093	-1.994148236	CUL3	Down
ENSOCUG00000012331	0.177774341	-2.491880985	UBR1	Down
ENSOCUG00000002152	0.390609654	-1.356200489	-	Down
ENSOCUG00000004915	0	-Inf	PLEKHS1	Down
ENSOCUG00000007121	0.422840713	-1.241813802	ATP6V0C	Down
ENSOCUG00000002315	0.463631516	-1.108949455	LIMK2	Down
ENSOCUG00000001320	0.284301601	-1.81450587	IFT74	Down
ENSOCUG00000022457	0.302682406	-1.724123275	MSANTD4	Down
ENSOCUG00000007397	0.105894037	-3.239306742	H1-1	Down
ENSOCUG00000037993	0	-Inf	-	Down
ENSOCUG00000000128	0.194947088	-2.358845486	C21orf91	Down
ENSOCUG00000001096	0.336452902	-1.57152353	-	Down
ENSOCUG00000017649	0.253357312	-1.980754628	ZBTB11	Down
ENSOCUG00000016539	0.301932054	-1.727704169	TNFRSF21	Down
ENSOCUG00000000141	0.264277721	-1.919873286	ACTR6	Down
ENSOCUG00000007182	0.116063003	-3.10701993	GAS2L3	Down
ENSOCUG00000013302	0.445950696	-1.165043878	CTR9	Down
ENSOCUG00000022455	0.264004874	-1.921363531	NRAS	Down
ENSOCUG00000017056	0.179011818	-2.481873264	PKN2	Down
ENSOCUG00000002154	0.227289382	-2.137397805	IBTK	Down
ENSOCUG00000005952	0.444239268	-1.17059117	BTBD10	Down
ENSOCUG00000012065	0	-Inf	PTPRR	Down
ENSOCUG00000001266	0.454002992	-1.139226288	TOMM70	Down
ENSOCUG00000017804	0.303824684	-1.718689008	-	Down
ENSOCUG00000004832	0.182114564	-2.457081793	USP47	Down
ENSOCUG00000027275	0.340237663	-1.555385244	TM4SF1	Down
ENSOCUG00000016016	0.257002863	-1.960143662	AKAP11	Down
ENSOCUG00000012874	0.135369348	-2.885026994	CRYBG3	Down

ENSOCUG00000010907	0.114755558	-3.12336406	CUL5	Down
ENSOCUG00000006411	0.434624933	-1.202157155	GPATCH3	Down
ENSOCUG00000004640	0.29615396	-1.755580717	ZRANB2	Down
ENSOCUG00000001161	0.446031291	-1.16478317	HYPK	Down
ENSOCUG00000005163	0.116464061	-3.102043267	MAP2	Down
ENSOCUG00000004983	0.20599291	-2.27933341	R3HCC1L	Down
ENSOCUG00000000727	0.201037753	-2.314461646	NECTIN3	Down
ENSOCUG00000017394	0.264593039	-1.918152987	SCNN1G	Down
ENSOCUG00000012736	0.269206068	-1.893217165	HSPD1	Down
ENSOCUG00000010785	0.038067414	-4.715299603	-	Down
ENSOCUG00000009547	0.443804192	-1.1720048	-	Down
ENSOCUG00000020931	0.109084913	-3.196476515	SAMD9	Down
ENSOCUG00000013616	0.313854935	-1.6718302	-	Down
ENSOCUG00000027060	0.32596096	-1.617228909	-	Down
ENSOCUG00000007273	0.429013717	-1.220904318	NUFIP1	Down
ENSOCUG00000001861	0.225681012	-2.147643058	XRN1	Down
ENSOCUG00000006994	0.462449425	-1.112632499	F11R	Down
ENSOCUG00000002167	0.233411788	-2.099050673	ZZZ3	Down
ENSOCUG00000037790	0.147990398	-2.756424526	-	Down
ENSOCUG00000004466	0.352093925	-1.505967759	WDR37	Down
ENSOCUG00000013950	0.103026919	-3.278906765	KIF18A	Down
ENSOCUG00000013477	0.459036477	-1.123319295	CEP20	Down
ENSOCUG00000000699	0.103747802	-3.268847327	CENPE	Down
ENSOCUG00000017448	0.3269767	-1.612740259	VBP1	Down
ENSOCUG00000013922	0.43437435	-1.20298918	PSMD12	Down
ENSOCUG00000023847	0.199566357	-2.325059566	CEP57L1	Down
ENSOCUG00000009829	0.3342979	-1.580793804	PBRM1	Down
ENSOCUG00000012421	0.24734496	-2.015403592	PTPN13	Down
ENSOCUG00000002798	0.46875095	-1.09310648	ABHD12	Down
ENSOCUG00000011083	0.347315846	-1.525679862	CNOT6	Down
ENSOCUG00000004833	0.45786706	-1.126999316	-	Down
ENSOCUG00000016661	0.238329145	-2.06897271	DRC7	Down
ENSOCUG00000008840	0.442616505	-1.175870846	ATP10D	Down
ENSOCUG00000015294	0.282528365	-1.823532376	BCLAF3	Down
ENSOCUG00000030940	0.11395497	-3.133464251	FA2H	Down
ENSOCUG00000007831	0.427626885	-1.225575537	MALT1	Down
ENSOCUG00000015121	0.198042985	-2.336114497	LRRC4	Down
ENSOCUG00000012457	0.192128461	-2.379856843	GOLGB1	Down
ENSOCUG00000009878	0.244236998	-2.033646332	-	Down
ENSOCUG00000017317	0.290771882	-1.782040332	CDC42BPA	Down
ENSOCUG00000012121	0.199728527	-2.323887691	U2SURP	Down
ENSOCUG00000022768	0.399545148	-1.323569562	-	Down
ENSOCUG00000015612	0.4275656	-1.22578231	NOL11	Down
ENSOCUG00000001124	0.286053691	-1.805642136	ZC2HC1A	Down

ENSOCUG00000012519	0.40064301	-1.319610788	TIRAP	Down
ENSOCUG00000014552	0.434504677	-1.202556389	TIPRL	Down
ENSOCUG00000016892	0.452856987	-1.14287258	NOL10	Down
ENSOCUG00000013308	0.315169265	-1.665801244	-	Down
ENSOCUG00000008790	0.447372462	-1.160451642	KMT2E	Down
ENSOCUG00000014545	0.454348479	-1.138128845	PSEN1	Down
ENSOCUG00000001217	0.244815356	-2.03023404	MARCHF7	Down
ENSOCUG00000014557	0.25549413	-1.968637949	CEP57	Down
ENSOCUG00000013503	0.251125242	-1.993521043	FAM189A2	Down
ENSOCUG00000005313	0.277388425	-1.850020508	HAT1	Down
ENSOCUG00000013133	0.19528813	-2.356323831	NCKAP1	Down
ENSOCUG00000003433	0.051380792	-4.282627047	CSF2	Down
ENSOCUG00000005291	0.446100688	-1.164558722	RELCH	Down
ENSOCUG00000013471	0.142256008	-2.813438512	LIPG	Down
ENSOCUG00000004600	0.356562816	-1.487771835	RPS6KB1	Down
ENSOCUG00000010213	0.257974623	-1.954698938	UPF3B	Down
ENSOCUG00000017170	0.367556077	-1.44396372	-	Down
ENSOCUG00000003590	0.141429703	-2.821842947	TMC5	Down
ENSOCUG00000012398	0.277786205	-1.847953139	SLC30A1	Down
ENSOCUG00000026070	0.463461858	-1.109477481	FAM110A	Down
ENSOCUG00000010058	0.414087907	-1.271991024	ZBTB44	Down
ENSOCUG00000024778	0.086533831	-3.530591912	-	Down
ENSOCUG00000027456	0.457159575	-1.129230258	DSTN	Down
ENSOCUG00000005746	0.257345024	-1.958224209	KLK4	Down
ENSOCUG00000014835	0.177037728	-2.497871251	SMC4	Down
ENSOCUG00000008843	0.265603643	-1.912653161	RBM26	Down
ENSOCUG00000003829	0.474327794	-1.076043686	EZR	Down
ENSOCUG00000011103	0.321398973	-1.637562774	TRMT10C	Down
ENSOCUG00000004665	0.213170189	-2.2299224	FGD6	Down
ENSOCUG00000013781	0.265343524	-1.914066755	RSRC2	Down
ENSOCUG00000014267	0.260380056	-1.941309144	RDX	Down
ENSOCUG00000034440	0.349793786	-1.515423435	-	Down
ENSOCUG00000004962	0.180710812	-2.468245266	PAPOLG	Down
ENSOCUG00000016483	0.274829375	-1.86339188	NUFIP2	Down
ENSOCUG00000025879	0.475361707	-1.072902403	ARHGEF5	Down
ENSOCUG00000015427	0.195760151	-2.352840979	-	Down
ENSOCUG00000007333	0.197695726	-2.338646414	RBBP8	Down
ENSOCUG00000006287	0.073192063	-3.772168972	-	Down
ENSOCUG00000024795	0.229734637	-2.121959705	MNDA	Down
ENSOCUG00000001732	0.457889641	-1.126928169	TMEM184C	Down
ENSOCUG00000024334	0.475078603	-1.073761864	AP1M2	Down
ENSOCUG00000010109	0.205664858	-2.281632792	BIRC3	Down
ENSOCUG00000006793	0.38636227	-1.371973879	LMO7	Down
ENSOCUG00000022370	0.379758889	-1.39684436	PAIP2B	Down

ENSOCUG00000016021	0.434902274	-1.201236843	LRRC20	Down
ENSOCUG00000012181	0.449314288	-1.154203156	LEO1	Down
ENSOCUG00000007723	0.360306728	-1.472702505	NCK1	Down
ENSOCUG00000007383	0.36673138	-1.447204379	CCNT1	Down
ENSOCUG00000016052	0.380004238	-1.395912585	RAD17	Down
ENSOCUG00000009882	0.463713985	-1.108692859	NCAPH	Down
ENSOCUG00000036144	0.455793957	-1.133546296	-	Down
ENSOCUG00000012338	0.326853595	-1.613283531	DDX3X	Down
ENSOCUG00000017847	0.36247145	-1.464060729	TMTC2	Down
ENSOCUG00000007150	0.272046135	-1.878076761	VPS4B	Down
ENSOCUG00000002961	0.466857657	-1.098945349	WDR12	Down
ENSOCUG00000002224	0.300909963	-1.732596219	UBLCP1	Down
ENSOCUG00000038979	0.24069641	-2.054713471	-	Down
ENSOCUG00000009283	0.458804369	-1.124048964	-	Down
ENSOCUG00000016649	0.406780335	-1.297678157	SDR42E1	Down
ENSOCUG00000002047	0.212178001	-2.236653012	TRPM8	Down
ENSOCUG00000007656	0.377973761	-1.403642009	TEC	Down
ENSOCUG00000033989	0.24676693	-2.018779025	-	Down
ENSOCUG00000016600	0.470930217	-1.086414801	GABARAPL2	Down
ENSOCUG00000003815	0.184829763	-2.435731	ZFX	Down
ENSOCUG00000013583	0.37854397	-1.401467207	CDK17	Down
ENSOCUG00000011464	0.381233334	-1.391253825	CLDN1	Down
ENSOCUG00000016049	0.445756738	-1.16567149	COA7	Down
ENSOCUG00000014549	0.321030776	-1.639216487	FAM76B	Down
ENSOCUG00000007001	0	-Inf	CPA1	Down
ENSOCUG00000011339	0.256188808	-1.964720645	SC5D	Down
ENSOCUG00000036551	0.074799358	-3.740830293	-	Down
ENSOCUG00000011878	0.281128013	-1.830700875	SLC35F2	Down
ENSOCUG00000027961	0.232242144	-2.106298297	PELI1	Down
ENSOCUG00000001215	0.209745126	-2.253290811	HIPK3	Down
ENSOCUG00000005617	0.405861782	-1.300939602	-	Down
ENSOCUG00000029362	0.19460214	-2.361400519	ZNF148	Down
ENSOCUG00000007575	0.131754512	-2.924075729	DDIAS	Down
ENSOCUG00000010789	0.228705561	-2.128436649	TMEM168	Down
ENSOCUG00000029103	0.251691302	-1.990272734	COX3	Down
ENSOCUG00000000821	0.470579789	-1.087488734	-	Down
ENSOCUG00000000652	0.304142063	-1.717182741	-	Down
ENSOCUG00000015633	0.236305075	-2.081277481	CUL4B	Down
ENSOCUG00000010430	0.463933161	-1.108011124	KHDC4	Down
ENSOCUG00000004077	0.422125664	-1.244255552	SLC4A11	Down
ENSOCUG00000003534	0.455768866	-1.13362572	PPP2R5E	Down
ENSOCUG00000000020	0.174736731	-2.516745191	SMCHD1	Down
ENSOCUG00000007754	0.114795724	-3.122859192	CAMSAP2	Down
ENSOCUG00000016532	0.1900452	-2.395585511	ATP13A3	Down

ENSOCUG00000012323	0.443150093	-1.17413268	FBXO45	Down
ENSOCUG00000009190	0.465009027	-1.104669371	-	Down
ENSOCUG00000012386	0.258146044	-1.953740605	DCUN1D1	Down
ENSOCUG00000015583	0.203503624	-2.29687361	RNF6	Down
ENSOCUG00000004308	0.399698932	-1.323014376	-	Down
ENSOCUG00000023952	0.321663722	-1.636374861	NTNG1	Down
ENSOCUG00000006510	0.281994589	-1.826260614	C9orf72	Down
ENSOCUG00000023666	0.114457387	-3.127117513	SNX16	Down
ENSOCUG00000005170	0.195456627	-2.355079591	BRWD3	Down
ENSOCUG00000002765	0.257651352	-1.956507934	LUC7L3	Down
ENSOCUG00000006833	0.050202251	-4.316104131	DNAH12	Down
ENSOCUG00000013378	0.464620193	-1.105876239	PAK1IP1	Down
ENSOCUG00000011743	0.22902373	-2.126431008	SLU7	Down
ENSOCUG00000007055	0.491974523	-1.023344488	PLEKHA6	Down
ENSOCUG00000015866	0.455729257	-1.133751102	BPTF	Down
ENSOCUG00000008145	0.461311459	-1.116186966	SMNDC1	Down
ENSOCUG00000007043	0.331115571	-1.594593239	-	Down
ENSOCUG00000036167	0.42311372	-1.240882628	-	Down
ENSOCUG00000017748	0.237907786	-2.071525608	SPART	Down
ENSOCUG00000016630	0.143229829	-2.803596113	KIF21A	Down
ENSOCUG00000001874	0.216260055	-2.209160882	ATR	Down
ENSOCUG00000025297	0.153981079	-2.699175006	-	Down
ENSOCUG00000003239	0.063577664	-3.975336181	TMEM236	Down
ENSOCUG00000015734	0.295771059	-1.7574472	LIG4	Down
ENSOCUG00000014040	0.302134462	-1.726737346	ENDOD1	Down
ENSOCUG00000012668	0.170011585	-2.556295035	RICTOR	Down
ENSOCUG00000003617	0.350493065	-1.512542194	MTIF2	Down
ENSOCUG00000001975	0.433749325	-1.205066581	-	Down
ENSOCUG00000029249	0.102960367	-3.279838994	-	Down
ENSOCUG00000000534	0	-Inf	LCP2	Down
ENSOCUG00000015528	0.444995712	-1.168136661	C16orf72	Down
ENSOCUG00000003110	0.31468582	-1.668015924	PRMT9	Down
ENSOCUG00000001785	0.165726418	-2.593124498	STX19	Down
ENSOCUG00000006171	0.16813096	-2.572342684	MINDY2	Down
ENSOCUG00000010912	0.446054102	-1.164709389	KRTDAP	Down
ENSOCUG00000005480	0.111662984	-3.162777085	TAF4B	Down
ENSOCUG00000002975	0.244352046	-2.032966907	STXBP3	Down
ENSOCUG00000000896	0.429505148	-1.219252672	TTC33	Down
ENSOCUG00000007050	0.329895699	-1.599918126	RBM39	Down
ENSOCUG00000017253	0.479171751	-1.061385237	-	Down
ENSOCUG00000016737	0.222617296	-2.167362406	ZFR	Down
ENSOCUG00000001818	0.265678296	-1.91224772	KPNA4	Down
ENSOCUG00000023811	0	-Inf	H3C13	Down
ENSOCUG00000002814	0.123341571	-3.019268973	TOP2A	Down

ENSOCUG00000009931	0.369847123	-1.43499904	OSGIN2	Down
ENSOCUG00000009466	0.111300707	-3.167465343	HEPHL1	Down
ENSOCUG00000000868	0.006613329	-7.240407516	CCL20	Down
ENSOCUG00000001772	0.312859824	-1.676411687	SLC20A1	Down
ENSOCUG00000015198	0.321528116	-1.636983195	VCPIP1	Down
ENSOCUG00000037416	0.131587943	-2.925900788	-	Down
ENSOCUG00000007695	0.35760842	-1.483547389	ATG4C	Down
ENSOCUG00000011276	0.457990844	-1.126609338	TMA16	Down
ENSOCUG00000027593	0.093632449	-3.416847597	PSORS1C2	Down
ENSOCUG00000006096	0.310061454	-1.68937391	ANKRD50	Down
ENSOCUG00000004878	0.215112072	-2.216839604	ORC3	Down
ENSOCUG00000012019	0.318261684	-1.651714614	RNF20	Down
ENSOCUG00000004006	0.346800517	-1.527822044	SOCS4	Down
ENSOCUG00000004075	0.323675325	-1.627380708	LIN7C	Down
ENSOCUG00000004921	0.230956004	-2.114310043	DCLRE1A	Down
ENSOCUG00000006910	0.483086836	-1.049645556	CDCP1	Down
ENSOCUG00000000170	0.32654718	-1.614636644	NUP155	Down
ENSOCUG00000012211	0.248772116	-2.007103307	-	Down
ENSOCUG00000011601	0.228080473	-2.132385157	THOC1	Down
ENSOCUG00000014899	0.312108537	-1.679880273	MRE11	Down
ENSOCUG00000010928	0.470236958	-1.088540164	-	Down
ENSOCUG00000008342	0.373974312	-1.418988918	FGFR1OP2	Down
ENSOCUG00000017003	0.384268516	-1.379813315	RAB1A	Down
ENSOCUG00000017333	0.460522093	-1.118657724	MRS2	Down
ENSOCUG00000032694	0.25616157	-1.964874041	-	Down
ENSOCUG00000015531	0.388480171	-1.364087134	CEP350	Down
ENSOCUG00000024056	0.357908334	-1.482337957	PPWD1	Down
ENSOCUG00000007732	0.482941544	-1.050079521	PPARD	Down
ENSOCUG00000013274	0	-Inf	FAM71F2	Down
ENSOCUG00000015867	0.247985061	-2.011674883	ARFGEF1	Down
ENSOCUG00000008716	0.410580465	-1.284263107	NBEAL1	Down
ENSOCUG00000017950	0.209107048	-2.257686408	TAF2	Down
ENSOCUG00000012765	0.166611815	-2.585437384	-	Down
ENSOCUG00000022143	0.475107831	-1.073673107	-	Down
ENSOCUG00000015766	0.327953773	-1.608435623	PATJ	Down
ENSOCUG00000024977	0.285796393	-1.806940388	ACSBG1	Down
ENSOCUG00000002397	0.442696589	-1.175609838	UBE4A	Down
ENSOCUG00000008350	0.308555757	-1.69639688	ZMYM1	Down
ENSOCUG00000016028	0.473535514	-1.078455468	RPF2	Down
ENSOCUG00000031561	0.469870606	-1.089664575	TARS2	Down
ENSOCUG00000007323	0.454830963	-1.136597623	-	Down
ENSOCUG00000016741	0.389615066	-1.359878629	NKRF	Down
ENSOCUG00000012080	0.274032225	-1.867582538	SLC18A2	Down
ENSOCUG00000007344	0.351967225	-1.506487002	-	Down

ENSOCUG00000013211	0.313631871	-1.672855925	SPRTN	Down
ENSOCUG00000015381	0.253802892	-1.978219587	QSER1	Down
ENSOCUG00000002103	0.145286965	-2.783022821	STPG4	Down
ENSOCUG00000015247	0.455725108	-1.133764238	ZDHHC14	Down
ENSOCUG00000028155	0.153462714	-2.704039923	-	Down
ENSOCUG00000004016	0.344490368	-1.537464449	RNPC3	Down
ENSOCUG00000002249	0.339764719	-1.557392045	DHRS7	Down
ENSOCUG00000025834	0.404695957	-1.305089659	YAP1	Down
ENSOCUG00000008381	0.443564893	-1.17278291	EPB41L5	Down
ENSOCUG00000011265	0.344400091	-1.537842569	ZSCAN29	Down
ENSOCUG00000009146	0.187448114	-2.415436783	SCYL2	Down
ENSOCUG00000015383	0.341190105	-1.551352288	PLEKHM3	Down
ENSOCUG00000005723	0.259460874	-1.946411097	ATAD2B	Down
ENSOCUG00000000817	0.291839778	-1.776751557	SPAG9	Down
ENSOCUG00000021120	0.019572244	-5.675046995	MUC15	Down
ENSOCUG00000025398	0.276609969	-1.854074941	CWF19L2	Down
ENSOCUG00000017759	0.260261762	-1.941964729	PIGA	Down
ENSOCUG00000009295	0.164869843	-2.60060056	EEA1	Down
ENSOCUG00000016177	0.465186165	-1.104119905	EPB41L4B	Down
ENSOCUG00000013382	0.289449217	-1.78861784	STX3	Down
ENSOCUG00000006204	0	-Inf	UBASH3A	Down
ENSOCUG00000013635	0.293901964	-1.766593096	AFF4	Down
ENSOCUG00000006056	0.163574787	-2.611977703	MAP3K8	Down
ENSOCUG00000007493	0.166430121	-2.587011531	-	Down
ENSOCUG00000012809	0.282018107	-1.826140303	SLC25A24	Down
ENSOCUG00000004637	0.392378714	-1.349681316	KIN	Down
ENSOCUG00000012236	0.464331535	-1.106772829	TMEM30B	Down
ENSOCUG00000006432	0.195608416	-2.35395965	IPMK	Down
ENSOCUG00000009367	0.470205391	-1.088637015	SETD7	Down
ENSOCUG00000000429	0.423850248	-1.238373465	MPHOSPH8	Down
ENSOCUG00000033412	0.21369735	-2.226359079	-	Down
ENSOCUG00000001171	0.110881251	-3.172912659	NUF2	Down
ENSOCUG00000012023	0.455811712	-1.133490099	EIF2S1	Down
ENSOCUG00000023043	0.476758288	-1.068670075	TLE3	Down
ENSOCUG00000027568	0.152324028	-2.71478456	-	Down
ENSOCUG00000011573	0.283135693	-1.820434461	-	Down
ENSOCUG00000014244	0.261261296	-1.936434677	-	Down
ENSOCUG00000024536	0.14864247	-2.750081715	-	Down
ENSOCUG00000000724	0.453677437	-1.140261183	SLC7A7	Down
ENSOCUG00000009234	0.169938915	-2.556911833	USP45	Down
ENSOCUG00000012801	0.037662299	-4.730735119	CRISP3	Down
ENSOCUG00000007014	0.279771766	-1.837677717	SH3D19	Down
ENSOCUG00000029610	0.471930003	-1.083355201	B4GALT3	Down
ENSOCUG00000029486	0.280759091	-1.832595355	GSTA5	Down

ENSOCUG00000017918	0.260630608	-1.939921575	GNPDA2	Down
ENSOCUG00000010426	0.376216016	-1.410366826	AGFG1	Down
ENSOCUG00000001224	0.252804236	-1.98390746	-	Down
ENSOCUG00000013727	0.37893814	-1.399965743	-	Down
ENSOCUG00000005315	0.478951003	-1.06205002	TFAP2A	Down
ENSOCUG00000003867	0.376025275	-1.411098458	SON	Down
ENSOCUG00000015471	0.469906707	-1.089553735	PLAA	Down
ENSOCUG00000006715	0.269863211	-1.889699782	-	Down
ENSOCUG00000027759	0.254832674	-1.972377828	-	Down
ENSOCUG00000009908	0.241178034	-2.051829582	ERI2	Down
ENSOCUG00000003640	0.334744669	-1.578867016	SLC6A4	Down
ENSOCUG00000021896	0.271660545	-1.880123048	-	Down
ENSOCUG00000022009	0.312687736	-1.677205458	-	Down
ENSOCUG00000009628	0.213813576	-2.225574637	FRYL	Down
ENSOCUG00000001306	0.329242865	-1.602775918	SPTLC3	Down
ENSOCUG00000017796	0.17590389	-2.507140706	-	Down
ENSOCUG00000020917	0.441165438	-1.180608322	PIK3AP1	Down
ENSOCUG00000006646	0.242839917	-2.041922511	UBR3	Down
ENSOCUG00000012438	0.465800279	-1.102216591	NBEAL2	Down
ENSOCUG00000014031	0.066406515	-3.912531405	TGM6	Down
ENSOCUG00000026994	0.493514406	-1.018835897	TUBA4A	Down
ENSOCUG00000005685	0.468150369	-1.0949561	TRIP11	Down
ENSOCUG00000010337	0.281712895	-1.827702491	DOCK4	Down
ENSOCUG00000015847	0.28890388	-1.791338514	CSPP1	Down
ENSOCUG00000002706	0.463280338	-1.11004264	MYCBPAP	Down
ENSOCUG00000025164	0.481012347	-1.055854168	-	Down
ENSOCUG00000029385	0.189685857	-2.398315979	-	Down
ENSOCUG00000007093	0.192577995	-2.376485234	IPO7	Down
ENSOCUG00000014956	0.234466379	-2.092547029	-	Down
ENSOCUG00000006600	0.307824123	-1.699821799	BIRC6	Down
ENSOCUG00000004657	0.380799185	-1.392897705	NR2C1	Down
ENSOCUG00000002993	0.148445723	-2.751992572	WDR47	Down
ENSOCUG00000024669	0.080080705	-3.642401508	-	Down
ENSOCUG00000021294	0.220565392	-2.180721651	TMEM95	Down
ENSOCUG00000002888	0.474573313	-1.075297121	DDX21	Down
ENSOCUG00000030260	0.115090944	-3.119153782	-	Down
ENSOCUG00000016430	0.33909807	-1.560225524	SEC63	Down
ENSOCUG00000011869	0.020047629	-5.640424582	ELMOD1	Down
ENSOCUG00000004217	0.26053994	-1.940423542	STX17	Down
ENSOCUG00000006650	0.333373326	-1.584789421	ETF1	Down
ENSOCUG00000035129	0.158842523	-2.654330916	-	Down
ENSOCUG00000004659	0.470204957	-1.088638347	CCNH	Down
ENSOCUG00000026252	0.486348714	-1.039936992	-	Down
ENSOCUG00000029827	0.089873537	-3.475959814	-	Down

ENSOCUG00000005068	0.228791112	-2.127897087	KLF8	Down
ENSOCUG00000003016	0.333344474	-1.584914283	-	Down
ENSOCUG00000004486	0.332776245	-1.587375643	CHL1	Down
ENSOCUG00000002461	0.311807036	-1.681274612	EMC2	Down
ENSOCUG00000017407	0.483073185	-1.049686324	-	Down
ENSOCUG00000000750	0.468398488	-1.094191677	PRMT3	Down
ENSOCUG00000008096	0.467551648	-1.096802354	RPRD1A	Down
ENSOCUG00000002159	0.268033419	-1.899515206	PTPN12	Down
ENSOCUG00000012987	0.396043581	-1.336268901	-	Down
ENSOCUG00000015184	0.149196518	-2.74471423	MYBL1	Down
ENSOCUG00000002470	0.388293108	-1.364781996	KLF6	Down
ENSOCUG00000001622	0.456480675	-1.131374309	-	Down
ENSOCUG00000000407	0.060228776	-4.053403256	DCT	Down
ENSOCUG00000007436	0.258006907	-1.954518405	ZW10	Down
ENSOCUG00000004780	0.466269564	-1.100763833	ABRAXAS2	Down
ENSOCUG00000024272	0.412204603	-1.278567478	NEXN	Down
ENSOCUG00000014767	0.23381989	-2.096530435	PAG1	Down
ENSOCUG00000007539	0.102760839	-3.28263752	FANCM	Down
ENSOCUG00000003608	0.201196151	-2.313325389	DAAM1	Down
ENSOCUG00000011629	0.475357077	-1.072916457	ANKRD17	Down
ENSOCUG00000014589	0.439244709	-1.186903186	-	Down
ENSOCUG00000016980	0.47624938	-1.070210881	UTP6	Down
ENSOCUG00000017240	0.219077155	-2.190489045	CDC5L	Down
ENSOCUG00000012997	0.42567028	-1.232191728	EPHX3	Down
ENSOCUG00000025948	0.194646137	-2.361074378	RPS6KA3	Down
ENSOCUG00000009397	0.314806114	-1.667464534	STRBP	Down
ENSOCUG00000013561	0.477466634	-1.066528176	GFM1	Down
ENSOCUG00000015943	0.32121191	-1.638402708	-	Down
ENSOCUG00000013930	0.343789313	-1.540403395	NOC3L	Down
ENSOCUG00000012206	0.249329655	-2.003873609	DDX46	Down
ENSOCUG00000009465	0.150872711	-2.728596212	AGL	Down
ENSOCUG00000031526	0.070662587	-3.822909615	PMAIP1	Down
ENSOCUG00000010647	0.293803719	-1.767075437	-	Down
ENSOCUG00000010700	0.476054998	-1.070799838	URB2	Down
ENSOCUG00000016428	0.432487655	-1.209269141	RCBTB2	Down
ENSOCUG00000017576	0.491128616	-1.025827209	ARRDC1	Down
ENSOCUG00000014717	0.40349297	-1.309384557	-	Down
ENSOCUG00000030320	0.464541703	-1.106119979	RAP1A	Down
ENSOCUG00000014438	0.481104089	-1.055579034	-	Down
ENSOCUG00000015445	0.487303542	-1.037107385	SQSTM1	Down
ENSOCUG00000002100	0.389945307	-1.358656305	PLEKHA1	Down
ENSOCUG00000016808	0.483171111	-1.049393897	HSDL2	Down
ENSOCUG00000006663	0.263098551	-1.926324792	DIS3	Down
ENSOCUG00000006709	0.258716423	-1.950556459	MED13	Down

ENSOCUG00000003507	0.163937497	-2.608782217	HAUS6	Down
ENSOCUG00000009764	0.248816586	-2.006845438	GPD2	Down
ENSOCUG00000008401	0.287673048	-1.797498031	SCNN1B	Down
ENSOCUG00000025781	0.476915627	-1.068194039	GPATCH4	Down
ENSOCUG00000016298	0.382793715	-1.385360953	ATP6V1H	Down
ENSOCUG00000008269	0.209900732	-2.252220899	ANKRD26	Down
ENSOCUG00000015333	0.34356693	-1.541336917	ZKSCAN1	Down
ENSOCUG00000012082	0.354099568	-1.497773011	ATAD1	Down
ENSOCUG00000034682	0.073823088	-3.759784098	-	Down
ENSOCUG00000005647	0.430299665	-1.216586379	RP2	Down
ENSOCUG00000000338	0.495347671	-1.013486627	ATP1B3	Down
ENSOCUG00000008774	0.252367455	-1.986402218	PTEN	Down
ENSOCUG00000009771	0.391138969	-1.354246815	-	Down
ENSOCUG00000003807	0.467844483	-1.095899054	SUV39H1	Down
ENSOCUG00000000713	0.422180638	-1.244067679	TMEM135	Down
ENSOCUG00000003320	0.253498579	-1.979950435	DENND4C	Down
ENSOCUG00000014088	0.363239834	-1.461005675	ETNK1	Down
ENSOCUG00000011055	0.413518028	-1.273977869	SLC9A3	Down
ENSOCUG00000033491	0	-Inf	-	Down
ENSOCUG00000015457	0.48116259	-1.055403616	FAM98A	Down
ENSOCUG00000012587	0.483672287	-1.047898217	-	Down
ENSOCUG00000026707	0.340577191	-1.553946276	TRIM23	Down
ENSOCUG00000005019	0.272837861	-1.87388424	CAND1	Down
ENSOCUG00000009730	0.359651517	-1.475328404	BRCC3	Down
ENSOCUG00000015503	0.462168805	-1.11350821	ZC3H14	Down
ENSOCUG00000015626	0.22043475	-2.181576423	-	Down
ENSOCUG00000006378	0.226938913	-2.139624085	TOGARAM1	Down
ENSOCUG00000005133	0.14680595	-2.768017657	-	Down
ENSOCUG00000023214	0	-Inf	-	Down
ENSOCUG00000007137	0.317696797	-1.654277551	CYB5R4	Down
ENSOCUG00000013380	0.321130019	-1.638770562	ADAM9	Down
ENSOCUG00000009776	0.244972089	-2.029310713	ZHX1	Down
ENSOCUG00000004700	0.321598056	-1.636669407	-	Down
ENSOCUG00000007824	0.215575056	-2.213737842	G2E3	Down
ENSOCUG00000008723	0.335951632	-1.573674555	YTHDF3	Down
ENSOCUG00000027066	0.318120643	-1.652354103	-	Down
ENSOCUG00000008870	0.273008447	-1.872982506	BRWD1	Down
ENSOCUG00000000661	0.289498673	-1.788371357	CCNT2	Down
ENSOCUG00000020986	0.478341977	-1.063885695	RNF25	Down
ENSOCUG00000026859	0.482452576	-1.05154096	-	Down
ENSOCUG00000029090	0.337268171	-1.568031921	ND2	Down
ENSOCUG00000016888	0.443338605	-1.173519101	IARS1	Down
ENSOCUG00000030959	0.06320832	-3.98374171	IDI2	Down
ENSOCUG00000003533	0	-Inf	RASGRF2	Down

ENSOCUG00000029523	0.180457161	-2.470271703	-	Down
ENSOCUG00000029251	0.258328784	-1.95271969	-	Down
ENSOCUG00000004413	0.245792375	-2.024487936	AP1AR	Down
ENSOCUG00000025191	0.143206719	-2.803828917	-	Down
ENSOCUG00000004295	0.451867486	-1.146028344	ZRSR2	Down
ENSOCUG00000005515	0.461022771	-1.117090084	HIBCH	Down
ENSOCUG00000012992	0.231293891	-2.112200936	MTMR6	Down
ENSOCUG00000029177	0.287090394	-1.800423036	-	Down
ENSOCUG00000004284	0.391441439	-1.353131603	C12orf29	Down
ENSOCUG00000010580	0.310420088	-1.687706175	ELF1	Down
ENSOCUG00000033220	0.148653055	-2.749978982	-	Down
ENSOCUG00000016010	0.281084176	-1.830925856	LMBRD1	Down
ENSOCUG00000021010	0.495656177	-1.012588384	-	Down
ENSOCUG00000024997	0.494331136	-1.016450316	TUBB4A	Down
ENSOCUG00000006338	0.028390074	-5.138469582	-	Down
ENSOCUG00000011117	0.426166939	-1.230509419	-	Down
ENSOCUG00000021362	0.24801433	-2.011504616	HAUS3	Down
ENSOCUG00000012930	0.471971391	-1.083228682	PPP2R1B	Down
ENSOCUG00000007174	0.393445238	-1.345765247	SATB1	Down
ENSOCUG00000015229	0.164958125	-2.599828257	ATAD5	Down
ENSOCUG00000012575	0.450560951	-1.15020581	PTAR1	Down
ENSOCUG00000004221	0.097911384	-3.352379574	TYRP1	Down
ENSOCUG00000029407	0.241139501	-2.052060097	-	Down
ENSOCUG00000016515	0.240360583	-2.056727771	GJA9	Down
ENSOCUG00000000462	0.285755431	-1.807147177	OXR1	Down
ENSOCUG00000000516	0.494397712	-1.016256028	-	Down
ENSOCUG00000004136	0.239650793	-2.060994382	-	Down
ENSOCUG00000011906	0.486297846	-1.040087895	ALDH3A2	Down
ENSOCUG00000016197	0.492589098	-1.021543397	RHBDL2	Down
ENSOCUG00000011900	0.403132634	-1.310673519	ZNF420	Down
ENSOCUG00000022126	0.156799879	-2.673003648	HMMR	Down
ENSOCUG00000000422	0.327014086	-1.612575313	ZACN	Down
ENSOCUG00000011295	0.487941911	-1.035218687	EVA1A	Down
ENSOCUG00000003634	0.493344855	-1.019331631	PCBP1	Down
ENSOCUG00000006282	0.191975206	-2.381008098	GCNT3	Down
ENSOCUG00000022694	0.271606254	-1.880411397	-	Down
ENSOCUG00000015263	0.486940601	-1.038182298	-	Down
ENSOCUG00000000353	0.477575318	-1.066199817	MTX1	Down
ENSOCUG00000016986	0.453259801	-1.141589878	-	Down
ENSOCUG00000027918	0.182117367	-2.45705959	-	Down
ENSOCUG00000001758	0.460781918	-1.117843991	PTPN2	Down
ENSOCUG00000005048	0.488847951	-1.03254229	SENP2	Down
ENSOCUG00000005741	0.485345883	-1.042914842	MARCHF6	Down
ENSOCUG00000006428	0.20293698	-2.300896314	-	Down

ENSOCUG00000002847	0.223771219	-2.159903605	RLIM	Down
ENSOCUG00000007235	0.403456497	-1.309514974	TRDMT1	Down
ENSOCUG00000005009	0.137693606	-2.860466522	PLK4	Down
ENSOCUG00000009575	0.49298038	-1.020397865	CIAO2A	Down
ENSOCUG00000017522	0.372854421	-1.423315647	TGS1	Down
ENSOCUG00000002694	0.445702327	-1.165847602	KPNA1	Down
ENSOCUG00000017517	0.336521461	-1.571229584	TMEM68	Down
ENSOCUG00000006900	0.481266333	-1.055092592	PIAS2	Down
ENSOCUG00000013715	0.305595336	-1.710305569	C5orf34	Down
ENSOCUG00000002192	0.135201875	-2.886812933	PGAP1	Down
ENSOCUG00000022283	0.484252824	-1.046167632	DCUN1D3	Down
ENSOCUG00000008509	0.367036708	-1.446003739	PAN3	Down
ENSOCUG00000015473	0.483705745	-1.047798423	XKR6	Down
ENSOCUG00000013330	0.411746068	-1.28017322	LARP1B	Down
ENSOCUG00000006721	0.290204319	-1.784859106	USP33	Down
ENSOCUG00000006277	0.480166894	-1.058392156	BNIP2	Down
ENSOCUG00000009419	0.377354893	-1.406006116	APPBP2	Down
ENSOCUG00000005763	0.420258137	-1.250652341	FAM177A1	Down
ENSOCUG00000034962	0.321185366	-1.638521933	-	Down
ENSOCUG00000024067	0.383579907	-1.382400944	-	Down
ENSOCUG00000006426	0.495571662	-1.012834402	-	Down
ENSOCUG00000012361	0.315348789	-1.664979703	ATF7IP	Down
ENSOCUG00000012315	0.385691367	-1.37448124	RNF168	Down
ENSOCUG00000012322	0.4972956	-1.007824429	MDFI	Down
ENSOCUG00000005680	0.275180284	-1.861550989	-	Down
ENSOCUG00000037401	0.189600204	-2.398967581	CNTD1	Down
ENSOCUG00000009932	0.14771978	-2.759065078	ESCO2	Down
ENSOCUG00000011400	0.421032471	-1.247996593	-	Down
ENSOCUG00000008969	0.159993575	-2.643914127	TIE1	Down
ENSOCUG00000007135	0	-Inf	FLACC1	Down
ENSOCUG00000009999	0.497259672	-1.007928661	-	Down
ENSOCUG00000026751	0.492047397	-1.023130805	PLBD1	Down
ENSOCUG00000013493	0.486536332	-1.039380554	SLC7A1	Down
ENSOCUG00000016756	0.141927802	-2.816770874	NEK1	Down
ENSOCUG00000037473	0.120496722	-3.052934192	ZNF300	Down
ENSOCUG00000008565	0.439883473	-1.184806695	PSMD1	Down
ENSOCUG00000007198	0.253678662	-1.978925923	USP9X	Down
ENSOCUG00000017459	0.120507471	-3.052805506	EPB42	Down
ENSOCUG00000010612	0.49027307	-1.028342576	YTHDF2	Down
ENSOCUG00000004759	0.494529635	-1.015871117	GSPT1	Down
ENSOCUG00000014568	0.323739373	-1.627095257	MTMR2	Down
ENSOCUG00000005030	0.306145064	-1.707712671	WASHC4	Down
ENSOCUG00000013064	0.490152416	-1.028697661	TLN2	Down
ENSOCUG00000022536	0.071563986	-3.804622437	SPP1	Down

ENSOCUG00000011896	0.328147144	-1.60758522	FBXL3	Down
ENSOCUG00000015158	0.458827775	-1.123975366	COG6	Down
ENSOCUG00000007351	0.123215613	-3.020743015	SCML2	Down
ENSOCUG00000002137	0.351671454	-1.507699862	-	Down
ENSOCUG00000005016	0.426732477	-1.228596182	NT5C3A	Down
ENSOCUG00000014811	0.177870133	-2.491103814	KIAA1109	Down
ENSOCUG00000026731	0.182010468	-2.457906665	HELLS	Down
ENSOCUG00000012469	0.366614337	-1.447664888	DYNLT4	Down
ENSOCUG00000016413	0.235063241	-2.088879147	CLTC	Down
ENSOCUG00000001881	0.441152702	-1.180649975	ING3	Down
ENSOCUG00000016878	0.13038911	-2.939104715	BRCA2	Down
ENSOCUG00000024179	0.46715202	-1.098035988	-	Down
ENSOCUG00000004220	0.477817062	-1.065469726	ERP44	Down
ENSOCUG00000012505	0.34270386	-1.544965655	LIPM	Down
ENSOCUG00000005559	0.179690306	-2.476415514	TBC1D9	Down
ENSOCUG00000022350	0.486157227	-1.040505128	CLOCK	Down
ENSOCUG00000004996	0.489272162	-1.031290894	EIF3A	Down
ENSOCUG00000010815	0.283234022	-1.819933522	CDC27	Down
ENSOCUG00000003868	0.481486549	-1.0544326	SLC38A7	Down
ENSOCUG00000013853	0.495152544	-1.014055043	NCBP1	Down
ENSOCUG00000011824	0.492389851	-1.022127069	RIOK1	Down
ENSOCUG00000032312	0.3214841	-1.637180709	-	Down
ENSOCUG00000025395	0.461343611	-1.116086416	-	Down
ENSOCUG00000012301	0.479027197	-1.061820527	-	Down
ENSOCUG00000006616	0.492420467	-1.022037368	EFCAB14	Down
ENSOCUG00000016788	0.45748584	-1.128201005	FAM114A2	Down
ENSOCUG00000022888	0.32840158	-1.606467028	-	Down
ENSOCUG00000010661	0.474513739	-1.075478235	PPIL3	Down
ENSOCUG00000000194	0.466765029	-1.099231618	EGR3	Down
ENSOCUG00000012495	0.054133269	-4.207340672	LIPN	Down
ENSOCUG00000017307	0.236071552	-2.082703898	-	Down
ENSOCUG00000002084	0.268293189	-1.898117663	UFL1	Down
ENSOCUG00000010286	0.410979313	-1.282862317	DOCK3	Down
ENSOCUG00000014366	0.216927036	-2.204718225	UGCG	Down
ENSOCUG00000002718	0.307735936	-1.70023517	-	Down
ENSOCUG00000007601	0.279024822	-1.841534626	MBNL1	Down
ENSOCUG00000021559	0.479592949	-1.060117648	ACER3	Down
ENSOCUG00000017633	0.350680356	-1.511771478	-	Down
ENSOCUG00000013020	0.353078006	-1.50194114	ADGRF4	Down
ENSOCUG00000022333	0.304878317	-1.713694546	-	Down
ENSOCUG00000021618	0.018988085	-5.718761804	SERPINB12	Down
ENSOCUG00000029442	0.44331034	-1.173611082	STYX	Down
ENSOCUG00000022702	0.326344537	-1.615532205	-	Down
ENSOCUG00000003204	0.493769944	-1.018089075	MAP3K9	Down

ENSOCUG00000003228	0.393968222	-1.343848831	CLK4	Down
ENSOCUG00000007696	0.425908952	-1.231383041	-	Down
ENSOCUG00000015970	0.215863623	-2.211807953	DENND1B	Down
ENSOCUG00000003465	0.441188386	-1.180533283	PLPP6	Down
ENSOCUG00000023139	0.471494166	-1.084688175	-	Down
ENSOCUG00000006039	0.176263779	-2.504192056	-	Down
ENSOCUG00000016098	0.379118529	-1.399279127	FGFBP1	Down
ENSOCUG00000017070	0.232725994	-2.103295736	P2RY4	Down
ENSOCUG00000027632	0.425389386	-1.23314406	-	Down
ENSOCUG00000000707	0.418474848	-1.256787183	-	Down
ENSOCUG00000027424	0.374055653	-1.41867516	PRPF38B	Down
ENSOCUG00000006624	0.264155435	-1.920541002	PDS5B	Down
ENSOCUG00000016615	0.279179452	-1.840735332	SMG1	Down
ENSOCUG00000029392	0.476471034	-1.069539582	-	Down
ENSOCUG00000013099	0.412148951	-1.278762271	SLC35F5	Down
ENSOCUG00000000649	0.297048049	-1.75123178	SLC36A4	Down
ENSOCUG00000014679	0.405620041	-1.301799158	FNDC3A	Down
ENSOCUG00000007566	0.174472619	-2.51892745	ACTA1	Down
ENSOCUG00000000486	0.473748728	-1.077806026	SPATA5	Down
ENSOCUG00000014903	0.318906964	-1.648792493	-	Down
ENSOCUG00000017023	0.491855031	-1.023694936	ATF1	Down
ENSOCUG00000009417	0.174374125	-2.519742114	PDE5A	Down
ENSOCUG00000030837	0.271036766	-1.883439529	-	Down
ENSOCUG00000004750	0.415765192	-1.266159113	GRHL2	Down
ENSOCUG00000002334	0.363585014	-1.459635359	-	Down
ENSOCUG00000013069	0.377033485	-1.407235437	ELOVL6	Down
ENSOCUG00000002603	0.477446939	-1.066587687	QTRT2	Down
ENSOCUG00000006698	0.217401902	-2.20156353	TNF	Down
ENSOCUG00000000575	0.489696688	-1.030039655	-	Down
ENSOCUG00000007335	0	-Inf	HS3ST2	Down
ENSOCUG00000009935	0.243348832	-2.038902244	NBN	Down
ENSOCUG00000013902	0	-Inf	SPACA4	Down
ENSOCUG00000011293	0.434340651	-1.203101112	YME1L1	Down
ENSOCUG00000015423	0.111558625	-3.164126042	ZDHHC23	Down
ENSOCUG00000000481	0.477268615	-1.067126628	EIF2S2	Down
ENSOCUG00000009927	0.297123408	-1.750865826	HERC4	Down
ENSOCUG00000014232	0.427847315	-1.224832059	CLINT1	Down
ENSOCUG00000009180	0.213191553	-2.229777815	FZD6	Down
ENSOCUG00000003910	0.478181157	-1.064370813	ZNHIT6	Down
ENSOCUG00000007398	0.491938118	-1.023451248	DR1	Down
ENSOCUG00000004242	0.489637533	-1.030213943	TEX10	Down
ENSOCUG00000001152	0.49510409	-1.014196227	LAMTOR3	Down
ENSOCUG00000011143	0.492243377	-1.0225563	FOSL1	Down
ENSOCUG00000012407	0.480240201	-1.058171917	MID2	Down

ENSOCUG00000023746	0.233899331	-2.096040363	-	Down
ENSOCUG00000039186	0.298475509	-1.744315535	-	Down
ENSOCUG00000000370	0.234593582	-2.091764549	ADAMTS8	Down
ENSOCUG00000008388	0.399723288	-1.322926467	CCNG2	Down
ENSOCUG00000000286	0.348405595	-1.521160303	EFCAB7	Down
ENSOCUG00000004696	0.247516723	-2.014402096	-	Down
ENSOCUG00000009154	0.35424282	-1.497189483	-	Down
ENSOCUG00000009347	0	-Inf	SSTR2	Down
ENSOCUG00000000625	0.200920262	-2.315305037	VPS50	Down
ENSOCUG00000010513	0.171007228	-2.547870789	CEP55	Down

Table S2. Data filtering statistics

Sample	Clean Reads No.	Clean Data (bp)	Clean Reads %	Clean Data %
Control_1	48450006	7315950906	95.46	95.46
Control_2	43679334	6595579434	95.1	95.1
Control_3	49556618	7483049318	95.16	95.16
Exos_1	43524030	6572128530	95.45	95.45
Exos_2	45633074	6890594174	95.38	95.38
Exos_3	52977250	7999564750	95.55	95.55

Note:

Clean Reads No: The number of high-quality sequence reads;

Clean Data (bp): the number of high-quality sequence bases;

Clean Reads %: The percentage of high-quality sequence reads in sequencing reads;

Clean Data %: The percentage of high-quality sequence bases to sequenced bases.

Table S3. RNASeq Map Statistics.

Sample	Clean_Reads	Total_Mapped	Multiple_Mapped	Uniquely_Mapped
Control_1	48450006	42153959 (87.01%)	2242156 (5.32%)	39911803 (94.68%)
Control _2	43679334	39425995 (90.26%)	2142961 (5.44%)	37283034 (94.56%)
Control _3	49556618	44534361 (89.87%)	2792734 (6.27%)	41741627 (93.73%)
Exos_1	43524030	37950001 (87.19%)	1817977 (4.79%)	36132024 (95.21%)
Exos_2	45633074	39979433 (87.61%)	1892914 (4.73%)	38086519 (95.27%)
Exos_3	52977250	45766728 (86.39%)	2058756 (4.50%)	43707972 (95.50%)

Note:

Clean Reads: The total number of sequences used for alignment;

Total Mapped: The total number of sequences in the reference genome in the alignment, the percentage is Total Mapped / Clean Reads;

Multiple Mapped: The total number of sequences aligned to multiple positions, the percentage is Multiple Mapped / Total Mapped;

Uniquely Mapped: The total number of sequences aligned to only one position, the percentage is Uniquely Mapped / Total Mapped.

Table S4. Information of primers used for qRT-PCR.

Primer	Sequence（5’→3’）

BMP4	F: TGTCAACTCCAGCATCCCCAA<br>R: ACCCACACCCCTCTACTACCAT
LEF1	F: CATCTCGGGTGGATTCAGG<br>R: AAACTCCCGTGACACCATCC
IGF1R	F: ACGAGTGGAGAAATCTGCGG<br>R: GTAGTTGCGGTAGTCCTCGG
TGFβ3	F: TCTCTGTCCACTTGCACCAC<br>R: CCGACTCGGTGTTTTCCTGA
TGFα	F: ACGTGTGTGCTGATACACTGC<br>R: AAGGCCTTTCTTCTGCAAGC
FGFR1	F: TAGCGAGTTTATTCCTGGCTCT<br>R: CTCAGGCTGGAACTCCTCGAA
FOSL1	F: GCCCAGCGAACAGATCAGCC<br>R: TCGTCCTCCAGCTTGTCCGTCT
KRT17	F: AGCTGCTACAGCTTCGGCTCG<br>R: CAGGCGGTCGTTGAGGTTCT
BCL2	F: ACATCGCCCTGTGGATGACTG<br>R: CGAGGGTGATGCAAGCTCCTAT
BMP2	F: GACTTCAACAGTGCCACC<br>R: TGCTGTAGCCAAATTCGT
CCND1	F: GAACGCTACCTTCCCCAGTGCTC<br>R: CCTCACAGACCTCCAGCATCCAG
CTNNB1	F: ACATTCTCACAGAGCCCGACCC<br>R: AGCAATCACACTCTGCATAGCGTTC
EGF	F: GTGCGATGCCGAGAAGT<br>R: AGCCCAATCTGAGAACCAC
GAPDH	F: CACCAGGGCTGCTTTTAACTCT<br>R: CTTCCCGTTCTCAGCCTTGACC

Table S5. Information of primers used for qRT-PCR.

Primer	Sequence（5'→3'）
pcDNA3.1-LEF 1	F: tagtccagtgtggtggaattcATGCCCCAGCTCTCCGGG<br>R: ggtttaaacgggccctctagaTCAGATGTAGGCAGCTGTCATTCT
siRNA-1915	sense：GCAUCCCUCAUCCAGCUAUTT<br>antisense：AUAGCUGGAUGAGGGAUGCTT
siRNA-2088	sense：GCAAAUGUCGUAGCUGAGUTT<br>antisense：ACUCAGCUACGACAUUUGCTT
siRNA-2216	sense：GCUACAUAUGCAGCUUUAUTT<br>antisense：AUAAAGCUGCAUAUGUAGCTT
siRNA-NC	sense：GTGCGATGCCGAGAAGT<br>antisense：AGCCCAATCTGAGAACCAC
